# Interventions that address institutional child maltreatment: An evidence and gap map

**DOI:** 10.1002/cl2.1139

**Published:** 2021-03-09

**Authors:** Meghan Finch, Rebecca Featherston, Sangita Chakraborty, Ludvig Bjørndal, Robyn Mildon, Bianca Albers, Caroline Fiennes, David J. A. Taylor, Rebecca Schachtman, Taoran Yang, Aron Shlonsky

**Affiliations:** ^1^ National Centre for Implementation Science Newcastle University Wallsend New South Wales Australia; ^2^ Department of Social Work, School of Primary and Allied Health Care, Faculty of Medicine Nursing & Health Sciences Monash University Caulfield Victoria Australia; ^3^ Centre for Evidence and Implementation Melbourne Victoria Australia; ^4^ Giving Evidence Cornwall UK

## Abstract

**Background:**

Child maltreatment has serious short and long‐term negative impacts for those experiencing it. Child maltreatment occurring in institutional settings has recently received substantial attention. However, evidence about the effectiveness of interventions that prevent, disclose, respond to, or treat maltreatment that has occurred in these environments is fragmented and can be difficult to access. This evidence and gap map (EGM) collates this research evidence. It was developed as a resource for stakeholders operating in the child health, welfare and protection sectors, including practitioners, organisational leaders, policy developers and researchers, wanting to access high quality evidence on interventions addressing institutional child maltreatment.

**Objectives:**

The objectives of this EGM were twofold: (a) To provide a structured and accessible collection of existing evidence from finalised and ongoing overviews of systematic reviews, systematic reviews and effectiveness studies of interventions addressing institutional child maltreatment—for those who work to fund, develop, implement and evaluate interventions aimed at ensuring children's safety in institutional settings; (b) to identify gaps in the available evidence on interventions addressing institutional child maltreatment—thereby helping to inform the research agendas of funders and other organisations.

**Search Methods:**

A comprehensive search strategy identified relevant studies from published and grey literature, comprising: (1) 10 electronic academic databases; (2) five trial and systematic review registries; (3) nine organisational websites; (4) websites and reference lists of inquiry reports associated with seven international inquiries into child abuse and (4) the lists of included studies within systematic reviews identified by the search strategy. Members of this EGM's Subject Matter Experts group were also invited to forward relevant unpublished studies or grey literature.

**Selection Criteria:**

The selection criteria were developed to identify finalised and ongoing overviews of reviews, systematic reviews and primary studies that reported on the effectiveness of interventions addressing child maltreatment (including sexual abuse, physical abuse, neglect and emotional abuse) within institutional settings. Eligible effectiveness study designs included: randomised controlled trials (RCTs), nonrandomised trials, controlled before‐and‐after studies and quasi‐experimental studies. Reviews were eligible if they reported a systematic literature search strategy.

**Data Collection and Analysis:**

All screening, data extraction, coding and critical appraisals were undertaken by two or more reviewers working independently, with discrepancies resolved via consensus or by a third reviewer. The titles and abstracts of studies identified by the search strategy were screened, and each full text of potentially relevant studies was further assessed for inclusion. Key data were extracted from all included studies and reviews. This included information about: publication details (e.g., year, author, country), inclusion/exclusion criteria (for reviews), study design, institutional setting, target population, type of maltreatment, intervention type and outcomes. Critical appraisal of included systematic reviews was achieved using the AMSTAR 2 tool, and completed RCTs were assessed using the updated Cochrane Risk of Bias 2.0 tool.

**Main Results:**

**Number of studies**

The electronic database search yielded 6318 citations, and a further 2375 records were identified from additional sources. Following deduplication and title/abstract screening, 256 studies remained for full text review. A total of 73 eligible studies (reported across 84 publications) met the inclusion criteria, including: 11 systematic reviews (plus, one update); 62 primary studies (including, three protocols for primary studies).

**Study characteristics**

The studies were conducted across 18 countries, however more than half (52%) were undertaken in the United States. Overall, most studies evaluated curriculum‐based interventions delivered in educational settings, primarily aimed at the prevention of sexual abuse. Institutional setting: Most studies evaluated interventions in school or early learning environments (*n* = 8 systematic reviews; *n* = 58 primary studies). Far fewer studies examined other organisational settings. Out of home care (including foster care, residential care and orphanages), and social service organisations servicing children were minimally represented. No studies were identified where the primary setting was sports clubs, churches/religious organisations, summer/vacation camps, detention centres/juvenile justice settings, or primary/secondary health care facilities. Target population: Most interventions targeted children rather than adults (*n* = 7 systematic reviews; *n* = 47 primary studies) from the general population. Fewer studies included populations known to be at an increased risk, or those already exposed to maltreatment. Just over a third of the primary studies conducted an analysis to ascertain differences in the effect of an intervention between the genders. Intervention type: Prevention interventions were the most studied (*n* = 5 systematic reviews; *n* = 57 primary studies), with additional studies including prevention approaches alongside other intervention types. Fewer studies evaluated interventions targeting disclosure, institutional responses, or treatment interventions. Type of maltreatment: The vast majority of the studies assessed interventions solely addressing the sexual abuse of children (*n* = 8 systematic reviews; *n* = 45 primary studies). The remaining studies addressed other forms of maltreatment, including physical and emotional abuse, or neglect, either in combination or as a sole focus. Outcomes: Primary reported outcomes reflected the bias toward child targeted interventions. Outcome measures captured child wellbeing and knowledge outcomes, including measures of mental health, children's knowledge retention and/or self‐protective skills. Measures of maltreatment disclosure or maltreatment occurrence/reoccurrence were less common, and all other outcome categories included in the EGM were minimally or not reported. A third of studies reported on some measure of implementation.

**Study quality**

The overall quality of the studies was low to moderate. Most systematic reviews were low‐quality (*n* = 10), with only one high quality review (and update) identified. Most completed RCTs had some concerns relating to the risk of bias (*n* = 30), and the remainder were considered to be at a high risk of bias (*n* = 19).

**Authors' Conclusions:**

This EGM has highlighted a substantial need for more high quality studies that evaluate interventions across a broader range of institutional contexts and maltreatment types. The current evidence base does not represent countries with large populations and the greatest incidence of child maltreatment. Few studies focussed on perpetrators or the organisational environment. Further evidence gaps were identified for interventions relating to disclosure, organisational responses and treatment, and few studies assessed interventions targeting perpetrators' maltreatment behaviours, recidivism or desistence. Future studies should also include measure of programme implementation.

## 1 PLAIN LANGUAGE SUMMARY

### 1.1 There is a lack of evidence on interventions addressing institutional child maltreatment

Child maltreatment affects millions of children, adults and communities globally. Research on institutional maltreatment is spread across multiple sources and can be difficult for stakeholders to locate.

This EGM provides a “go to” resource that presents existing evidence evaluating the effectiveness of interventions targeting the prevention, disclosure, response to and treatment of institutional child maltreatment. The map indicates that evidence supporting interventions addressing institutional child maltreatment is limited.

#### 1.1.1 What is this EGM about?

Child maltreatment, including sexual, physical or emotional abuse and neglect, negatively impacts the physical, mental, spiritual and interpersonal wellbeing of those experiencing and surviving it, in both the short term and the long term.

Child maltreatment occurring in institutional settings has recently gained substantial public and policy recognition through government inquiries. Institutional settings can include places of education, foster care, residential care or juvenile justice or health care settings.

**What is the aim of this evidence and gap map (EGM)?**

This EGM provides a “go to” resource presenting the existing evidence on the effectiveness of interventions addressing child maltreatment within institutional settings.

The relevant research can be difficult for stakeholders, such as policy makers, researchers, practitioners and others, to access and use because it is spread out across multiple sources.

#### 1.1.2 What studies are included?

Eligible studies were systematic reviews and primary studies that reported on the effectiveness of interventions addressing child maltreatment within institutional settings.

Seventy‐three eligible studies met the selection criteria, including: 11 systematic reviews, 59 primary studies and three protocols. The studies were conducted across 18 countries, with over half within the United States.

Most studies evaluated curriculum‐based interventions delivered in educational settings, aimed at preventing sexual abuse. Fewer studies examined other organisational settings, such as out‐of‐home care settings (including, foster care, residential care and orphanages). No studies explicitly assessed sports clubs, religious organisations, juvenile justice or health care settings.

Most interventions targeted children, rather than adults. Few studies included populations known to be at risk, or those already exposed to maltreatment. Prevention interventions were most studied, with few studies evaluating disclosure, institutional responses or treatment interventions. The majority of studies assessed interventions addressing sexual abuse, and far fewer addressed physical and emotional abuse, or neglect.

The reported outcomes reflected the bias toward child‐targeted interventions, and primarily captured child wellbeing and protective skills/knowledge outcomes. Measures of maltreatment disclosure or maltreatment occurrence/reoccurrence were less common, and all other outcome categories included in the EGM were minimally represented.

Only a third of studies reported measures of implementation. These included measures representing the feasibility, adoption, fidelity, acceptability and penetration of the interventions being evaluated.

#### 1.1.3 What are the main findings of this map?

This EGM indicates that evidence supporting interventions addressing institutional child maltreatment is limited. The map highlights a substantial need for more high‐quality studies that evaluate interventions across a broader range of institutional contexts and maltreatment types.

The evidence does not currently cover countries with large populations and those with the greatest incidence of child maltreatment. Few studies focussed on perpetrators or the organisational environment. There are evidence gaps for disclosure, organisational responses and treatment interventions, and few studies assessed interventions targeting perpetrators' behaviours, recidivism or desistence.

#### 1.1.4 What do the findings of the map mean?

More research is needed to address the gaps described above. Furthermore, future studies should include measures of programme implementation.

## 2 BACKGROUND

### 2.1 The problem, condition or issue

Child maltreatment is a widespread phenomenon affecting millions of children, adults and communities around the globe. Child maltreatment includes sexual abuse, physical abuse, neglect and emotional abuse. It is a major social issue that has a negative impact on the physical, mental, spiritual, educational and interpersonal wellbeing of those experiencing and surviving it—both in the short term and in the form of long‐term consequences that reduce the quality of life into adulthood (Fang & Corso, 2007; Fang et al., 2012; Felitti et al., 1998; Jaffee et al., 2018; Lueger‐Schuster et al., 2018; Maniglio, 2009; Moore et al., 2015; Teicher & Samson, 2016). In recent years, child maltreatment in institutional settings has received high public and policy recognition, and there is increasing interest in targeting this form of maltreatment.

Determining the prevalence of child maltreatment is considered difficult due to inconsistencies in measurement and suspected under‐reporting (Finkelhor et al., 2014), resulting in considerable variability among estimates. Even so, estimates of the overall prevalence of child maltreatment are alarmingly high, and these may give some indication of the extent of this issue. A synthesis of existing meta‐analyses from across the globe estimated overall prevalence at 127/1000 for sexual abuse, 226/1000 physical abuse, 363/1000 emotional abuse, 163/1000 for child neglect and 184/1000 for emotional neglect (Stoltenborgh et al., 2015). Prevalence rates are sensitive to a number of factors. There are both geographical and gender differences. For example, the Global Status report published by the World Health Organisation (WHO) reported the prevalence of child physical abuse in Swaziland to be 22%, whereas in countries including Kenya, Tanzania and Zimbabwe prevalence ranged between 53% and 76% with higher rates of abuse experienced by boys than girls (WHO, 2014). Rates can vary depending on whether incidences of maltreatment are self‐reported or based on informants (Greger et al., 2015; Moody et al., 2018), and can also vary with the identity of the perpetrator/s. The nature of the acts (how widely or narrowly different subtypes of maltreatment are defined and operationalised in studies) or how many items are used to measure prevalence, can also impact on rate estimates. While there is some variability across estimates, it is clear that the occurrence of child maltreatment is unacceptably high. The overall economic cost of child maltreatment is also high, with average lifetime costs in the US upward of $200,000 per child, resulting in billions in cost burden from new cases each year (Fang et al., 2012; Letourneau et al., 2018). In short, child maltreatment is harmful, highly prevalent and costly.

Even less is known about the prevalence of child maltreatment that occurs in institutional contexts, such as schools, out‐of‐home care, youth/juvenile detention, sport clubs, recreational settings, religious organisations, or other comparable child and youth serving organisations in which children live or spend time. In these settings, child maltreatment can encompass adults abusing children, children abusing other children, or institutions enabling child maltreatment. Children may be more or less vulnerable, or at risk, for reasons ranging from a lack of proper safeguarding in institutions (e.g., failing to respond to disclosures) (Australian Government, 2017; Lemaigre et al., 2017; Wurtele, 2012), to the characteristics of children (e.g., age, developmental or other disabilities) (Devries et al., 2018; Sullivan et al., 1992). Institutional child maltreatment as a field of empirical research is at an early stage (Blakemore et al., 2017; Proeve et al., 2016; Timmerman & Schreuder, 2014). It is not common for studies to differentiate between maltreatment occurring in institutional settings versus other maltreatment settings, and disentangling the impact of institutional maltreatment versus maltreatment that takes place in other contexts has not been routine. The empirical research to date has focussed primarily on sexual abuse within especially religious and out‐of‐home care institutions, whereas other types of maltreatment and settings have been less examined (Proeve et al., 2016). Recent studies conducted in Germany (Allroggen et al., 2018) and Norway (Greger et al., 2015) confirm that children placed in institutional care are at significantly higher risk of experiencing maltreatment, but less is known about maltreatment taking place in areas such as sports and exercise settings (Bjørnseth & Szabo, 2018). Regardless, it is clear that child maltreatment taking place in these settings affects the lives of both victims, their families and their communities—at times for generations.

Child maltreatment occurring in institutional settings has received substantial attention in recent years, both at the policy level, among practitioners and service agencies working with children in different capacities and roles, and also as part of the public discourse. The shift in attention and prioritisation of child maltreatment as a key concern of society is reflected in a broad range of official inquiries and associated reports conducted in recent years in especially high‐income countries—of which the following is a selected sample:

- Law Commission of Canada. Restoring dignity: Responding to child abuse in Canadian institutions [Canada]: Law Commission of Canada; 2012.
- Daniel B., Burgess C., Scott J. Review of child neglect in Scotland [Scotland]: Scottish Government; 2012.
- New Zealand House of Representatives. Inquiry into improving child health outcomes and preventing child abuse with a focus from preconception until three years of age [New Zealand]: New Zealand House of Representatives; 2014
- Australian Government. Royal commission into institutional responses to child sexual abuse [Australia]: Australian Government Royal Commission; 2018. Report No.: Vols 1–17.
- Northern Ireland Historical Institutional Abuse Inquiry 1922–1995 [Northern Ireland]: Historical Institutional Abuse Inquiry; 2017
- Pennsylvania Attorney General. Pennsylvania diocese victims report [Pennsylvania]: Attorney General; 2018
- Unabhängige Kommission zur Aufarbeitung sexuellen Kindesmissbrauchs [Germany]: Aufklärung von Ausmaß, Art, Umständen, Ursachen und Folgen von sexueller Gewalt gegen Kinder und Jugendliche in Deutschland seit dem Jahr 1945; 2016–2023
- Jay A., Evans M., Frank I., Sharpling D. Sexual abuse of children in custodial institutions: 2009–2017 Investigation report [United Kingdom]. Independent Inquiry Child Sexual Abuse; Crown copyright 2019

These inquiries have led to a prioritisation of child maltreatment within institutional settings, as both a specific and serious issue among policy‐makers, practitioners and service agencies working with children (Blakemore et al., 2017; Proeve et al., 2016). Indeed, the problem has now rightly gained much wider recognition, being under the purview of the United Nations and WHO, and gaining attention from parliaments, legislators, institutional governance and leadership, as well as the corporate and philanthropic sectors. The inquiries have led to the production of multiple research reports examining the specific characteristics and consequences of institutional child maltreatment (Blakemore et al., 2017), how it can be prevented (Pitts, 2015; South et al., 2014, 2015), victims supported (SHlonsky et al., 2017), perpetrators and institutions held accountable, and suitable responses implemented and maintained over time (Albers & Mildon, 2016; Parenting Research Centre, 2015).

Evidence about the effectiveness of interventions aimed at preventing, disclosing, responding to, or treating institutional child maltreatment is spread across multiple sources, and generally exists in the form of academic or grey literature. For institutions that wish to improve their practices and services in this area, it can be difficult and time consuming to find, access and interpret existing evidence. Therefore, there is still considerable confusion among sector stakeholders about what evidence exists for interventions developed to address institutional child maltreatment. Evidence synthesis is a powerful tool that can bring together, integrate and interpret diverse knowledge sources using methods that are comprehensive, transparent and replicable (Littell & Shlonsky, 2010; Straus et al., 2013). This EGM aims to provide a “go to” knowledge base for stakeholders wanting to access high level evidence on interventions addressing child maltreatment within institutions or organisations.

### 2.2 Scope of this EGM

The EGM is vertically structured into interventions aimed at institutional child maltreatment prevention, disclosure, institutional responses and treatment. The EGM's horizontal structure is formed by outcomes that relate to institutional safeguarding practices, maltreatment occurrence/reoccurence, children's health and wellbeing, parent/caregiver behaviour, knowledge and attitudes, and adult perpetrators of child maltreatment or child/youth offenders. These dimensions of the EGM are outlined in greater detail below.

The EGM includes effectiveness studies of different designs, including overviews of systematic reviews, systematic reviews, (cluster) RCTs and studies using quasi‐experimental designs. The EGM inclusion criteria were international in scope, and covered low‐, middle‐ and high‐income countries. These and other characteristics are described in greater detail below.

### 2.3 Conceptual framework of this EGM

Child maltreatment in institutional settings is a complex problem that may encompass (Australian Government, 2017):

- Adults abusing children,
- Children abusing other children,
- Institutions enabling child maltreatment and
- Child characteristics enhancing their vulnerability to maltreatment.

In addressing institutional child maltreatment, interventions may be aimed at:

- **Preventing the occurrence and reoccurrence of child maltreatment**. This may be based on universal services available to an entire target population and aimed at promoting positive behaviours and functioning and/or at decreasing risk factors and the likelihood of problems and challenges in a person's life. Or, targeted services available to selected members of a target population who are at risk of developing or experiencing particular problems—with the intervention aimed at reducing these risks.
- **Disclosing child maltreatment**. A key factor in stopping, responding to and treating the consequences of child maltreatment is its disclosure—especially in cases of child sexual abuse (Lemaigre et al., 2017; Paine & Hansen, 2002). Recent inquiries have documented the substantial barriers existing in institutional settings to facilitate such disclosure (Australian Government, 2017; Lemaigre et al., 2017), pointing to the importance of including disclosure interventions in this EGM.
- **Responding to the occurrence of child maltreatment**. Institutions have strong legal and ethical obligations to respond appropriately when child maltreatment has been detected or disclosed. This includes reporting the maltreatment, supporting the victim and/or family, working with child protection agencies, and providing training and crisis support to staff.
- **Treating the consequences of child maltreatment**. Providing services or referring children and families to agencies that provide therapeutic care for one or more of the many known problems associated with experiencing child maltreatment (e.g., posttraumatic stress disorder).

Based on this understanding, the EGM covered studies examining interventions aimed at preventing the occurrence and reoccurrence of child maltreatment, disclosing child maltreatment, responding to the occurrence of child maltreatment and/or treating its consequences. These interventions could be placed at all levels of the service spectrum and target either children or adults within the institutional setting, child offenders, adult perpetrators, or the institutional setting itself.

With regard to institutional settings, different organisational factors have been identified that purportedly increase or decrease the likelihood of institutional child maltreatment (Australian Government, 2017), including institutional:

- Cultural factors (e.g., leadership, organisational culture),
- Operational factors (e.g., governance, day‐to‐day work routines and practices) and
- Environmental factors (e.g., physical spaces)

Studies examining interventions addressing any of these organisational factors were therefore included in this EGM.

A more detailed outline of how this overarching framework was operationalised in the development of the full EGM has been presented in Section 4.

### 2.4 Why was it important to develop this EGM?

Given the lack of a “go to” global knowledge base presenting high quality evidence on the effectiveness of interventions that aim to protect children from harm occurring in institutional settings, the production of this EGM is timely. The knowledge generated has the potential to support numerous stakeholders.

- Institutions to identify potentially effective interventions and/or key characteristics of potentially effective interventions—knowledge that may be used to inform the selection and design of interventions to be used locally.
- Funding bodies and policymakers to make informed decisions related to the safeguarding of children in institutional settings, or around priority setting in research and development (e.g., targeting gaps in the current research base).
- Research organisations to assess the current evidence on child maltreatment in institutional settings and use this knowledge to inform the development of research agendas and priorities.
- The identification of existing topics for which there are sufficient primary studies to warrant the undertaking of separate systematic reviews (with or without meta‐analyses), where none currently exist.

### 2.5 Existing EGMs and/or relevant systematic reviews

To our knowledge, there are only three other EGMs that—in different ways—relate to issues of child maltreatment—all of which are registered with the Campbell Collaboration:

1. Kornør et al. (2017) is in development. It will focus on:

- Child maltreatment
- Children aged prenatal‐12 years
- Studies conducted in high‐income countries only.

The subject of this EGM is child abuse and neglect in general. It will identify evidence on interventions that prevent or reduce the harm of child maltreatment in at‐risk or exposed populations of children. It is not specifically focussed on institutional settings.

2. Saran and White (2018) has been developed in full and is available in the public domain. It focuses on:

- Child welfare,
- Children aged under 18 and
- Studies conducted in low‐ and middle‐income countries.

This EGM includes 302 systematic reviews on a broad range of child welfare interventions and outcomes, including child health and nutrition, and education. Interventions addressing child abuse make up a small component of this EGM. There is no particular focus on institutional settings, and studies conducted in high‐income countries were not included.

3. Pundir et al. (2019) is in development. It will focus on:

- Violence against children,
- Children under 18 years and
- studies conducted in low‐ and middle‐income countries.

This EGM will include evidence on the effectiveness of interventions aimed at reducing violence against children, including female genital mutilation, child marriage, bullying and child labour. There is no particular focus on institutional settings, and studies conducted in high‐income countries are not included.

Taken together, this means that the EGM described here is a genuine and much needed contribution to the evidence base on child maltreatment for two key reasons.

- It specifically focuses on institutional settings—which are not the key focus of any of the other EGMs and, therefore, may be at risk of disappearing in large amounts of other evidence regarding child maltreatment occurring in other contexts.
- It includes existing evidence from low‐, middle‐ and high‐income countries.

As such, it will be an important resource for a wide range of stakeholders operating in child and youth serving organisations, such as kindergartens, schools, charities, churches, sports clubs, scouting associations, out‐of‐home care providers and the many other organisations that associate with children. Given the scale of interest in this issue, it is also expected to be an important resource more broadly.

## 3 OBJECTIVES

The objectives of this EGM were twofold:

- To provide a structured and accessible collection of existing evidence from finalised and ongoing overviews of systematic reviews, systematic reviews and effectiveness studies of interventions addressing institutional child maltreatment—for those who work to fund, develop, implement and evaluate interventions aimed at ensuring children's safety in institutional settings.
- To identify gaps in the available evidence on interventions addressing institutional child maltreatment—thereby helping to inform the research agendas of funders and other organisations.

## 4 METHODS

### 4.1 Defining EGMs

Mapping the evidence in an existing area is a relatively new approach that has been used since the early 2000s (Saran et al., 2018). EGMs are “evidence collections” (Snilstveit et al., 2013, p. 3) that provide a visual overview of the availability of evidence for a particular sector—in this case, interventions addressing institutional child maltreatment. They belong to a group of evidence synthesis products that aim to “configure information” (Littell, 2018, p. 10). They do this by mapping out existing and ongoing systematic reviews and effectiveness studies, and by providing a graphical display of areas with strong, weak or nonexistent evidence on the effect of interventions or initiatives. EGMs therefore help to consolidate what evidence exists and what evidence does not currently exist about the effectiveness of interventions in a given area.

Studies included in an EGM are identified through a comprehensive search of published and unpublished literature, as well as trial registries, targeting both completed and ongoing studies. Ongoing studies help to identify research in development which might help fill existing evidence gaps in the future.

The methods for conducting EGMs draw on the principles and methodologies adopted in existing evidence mapping and synthesis products. Typically, six steps are taken when conducting an EGM:

#### 4.1.1 Step 1. Defining scope

The first step in producing an EGM is to set the scope by developing a framework, typically presented in a tabular format, which represents the universe of interventions and outcomes in the field to be covered. The rows of the framework represent all interventions relevant to the area covered, while columns include all relevant intervention outcomes.

#### 4.1.2 Step 2. Setting study inclusion criteria

As part of this step, the types of evidence to be included in the EGM are determined. EGMs often rely on two types of studies: (1) systematic reviews that critically appraise and synthesise all available evidence in a particular area and (2) primary studies that test effectiveness using rigorous experimental and quasi‐experimental designs.

#### 4.1.3 Step 3. Searching for studies and assessing inclusion

Next, a strategy for populating the EGM framework with studies meeting the study inclusion criteria is developed. The methods for doing so draw on approaches to systematic searching commonly used for systematic reviews and overviews of reviews.

#### 4.1.4 Step 4. Coding and critical appraisal

This step involves the systematic coding and extraction of data using a structured and standardised format. Studies are coded according to relevant intervention and outcome categories. Depending on the purpose of the EGM and the needs of stakeholders, other coding categories may also be relevant, including, for example, geographical scope of the evidence, demographic characteristic of target populations, study settings and so forth. The quality of the included systematic reviews and primary effectiveness studies is also appraised using established methods germane to systematic reviewing.

#### 4.1.5 Step 5. Producing user‐friendly summaries, presentations and analysis

A common feature of an EGM is that it provides direct access to user‐friendly plain language summaries. The method for this—and the final functionality of the map—will often depend on the resources available to produce the EGM.

#### 4.1.6 Step 6. Further disseminating knowledge derived from the EGM

Finally, the map itself and information about its key findings, will be disseminated to its key users and other stakeholders. For example, through presentations, webinars, research briefs and other means.

How these steps were undertaken for this EGM has been outlined in the following sections.

### 4.2 The EGM framework

The complete protocol for this EGM was published with Campbell Systematic Reviews (Albers et al., 2019).

#### 4.2.1 Target population

This EGM focused on the universe of interventions and outcomes for children:

- Aged under 18 years at the point of baseline measurement and
- Living in and/or engaging in activities in institutional settings.

Although children were the key target population, study participants could also be adults (see Section 4.3.1). This EGM aimed to include evidence on interventions that targeted perpetrators of institutional child maltreatment, as well as interventions aimed at improving the professional practice of staff and organisational standards of child and youth serving organisations.

#### 4.2.2 Intervention categories

This EGM was focused on four intervention categories: prevention, disclosure, response and treatment. Within each intervention category, intervention targets were specified as: victim, perpetrator and institution. Table 1 presents this EGM structure alongside relevant intervention examples. Systematic reviews in which only a subset of studies covered interventions eligible for inclusion, were included in the map, provided that the outcomes measures reported for these interventions were of relevance to this EGM.

**Table 1:** The four intervention categories within scope of this EGM, with examples

Intervention	Target	Examples
Prevention	Victim	– Universal/primary interventions (e.g., educational interventions used in school settings, maternal‐child health screening) – Indicated/tertiary interventions (e.g., advocacy, social supports)
	Perpetrator	– Universal/primary interventions (e.g., traditional or social media campaigns) – Targeted/secondary therapeutic interventions (e.g., CBT group therapy, education interventions) – Indicated/tertiary interventions (e.g., criminal justice, pre‐employment screening/criminal background checks)
	Institution	– Legal or regulatory mechanisms aimed at introducing new procedures for institutions to follow (e.g., response framework) – Particular institutions aimed at enhancing safeguarding practices of other institutions and outcomes in institutional settings (e.g., Child Advocacy Centres) – Organisational guidelines and/or practices – Staff education or training programs/initiatives
Disclosure	Victim	– Universal/primary interventions (e.g., Traditional or social media campaigns, abuse helplines) – Targeted/secondary therapeutic interventions (e.g., play therapy)
	Perpetrator	– Universal/primary interventions (e.g., Traditional or social media campaigns) – Legal or regulatory mechanisms aimed at introducing new procedures for institutions to follow (e.g., mandatory reporting)
	Institution	– Legal or regulatory mechanisms aimed at introducing new procedures for institutions to follow (e.g., response framework) – Particular institutions aimed at enhancing safeguarding practices of other institutions and outcomes in institutional settings (e.g., Child Advocacy Centres) – Organisational guidelines and/or practices (e.g., guidelines for reporting abuse) – Staff education or training programs/initiatives
Response	Victim	– Indicated/tertiary interventions (e.g., Legal avenues for criminal redress, advocacy, social supports)
	Perpetrator	– Indicated/tertiary interventions (e.g., criminal justice, arrest, removal of credentials, imprisonment)
	Institution	– Legal or regulatory mechanisms aimed at introducing new procedures for institutions to follow – Organisational guidelines and/or practices (e.g., response framework, perpetrator accountability) – Particular institutions aimed at enhancing safeguarding practices of other institutions and outcomes in institutional settings (e.g., Child Advocacy Centres) – Staff education or training programs/initiatives
Treatment	Victim	– Targeted/secondary therapeutic interventions (e.g., trauma‐focussed interventions)
	Perpetrator	– Indicated/tertiary interventions (e.g., criminal justice, arrest, removal of credentials, imprisonment)
	Institution	– Legal or regulatory mechanisms aimed at introducing new procedures for institutions to follow – Organisational guidelines and/or practices (e.g., response framework, perpetrator accountability)

#### 4.2.3 Outcome categories

This EGM was focused on six different outcome categories, outlined in Table 2. These categories were: institutional safeguarding practice; disclosure; child maltreatment occurrence/reoccurrence (child safety); child wellbeing, adult perpetrator/child or youth offender outcomes; and parent/caregiver outcomes.

**Table 2:** The six outcome categories included in the EGM

Outcome category	Subcategory	Examples
Institutional safeguarding practice	Culture	– Leadership behaviour (e.g., role modelling of safeguarding behaviour) – Staff perceptions or views associated with safeguarding practices or risk awareness/minimisation
	Operations	– Outcomes related to staff recruitment policy/practice – Outcomes related to the implementation of child safeguarding policies and practices – Outcomes related to adult institutional caregiver competencies, including: Knowledge and skills relating to institutional policies and practices required to safeguard children Knowledge about child maltreatment and its impact on children Knowledge about risk factors for child maltreatment, observation and interview skills related to identifying child maltreatment Ability to handle child maltreatment disclosure including listening, supporting, documenting and actioning a response Competencies associated with supporting and working with children who have been maltreatment
	Environment	– Outcomes associated with the design of, or modifications to, the institution's physical environment
Child maltreatment disclosure	Disclosure rates	– The disclosure of maltreatment through the victim, caregivers, institutional staff or others involved in the child's life
Child maltreatment occurrence or reoccurrence (child safety)	Maltreatment type (i.e., physical abuse, sexual abuse, neglect, emotional abuse)	– The occurrence or reoccurrence of child maltreatment within the institutional setting, for study participants—measured through, for example, self‐reports, informant‐reports
Child health and wellbeing	Knowledge/awareness	– Knowledge about child maltreatment and potential responses to offending behaviour – Risk‐aware/risk‐targeting behaviour
	Physical health	– Normative standards for growth and development – Gross motor and fine motor skills – Overall health – Body mass index – Risk‐avoidance behaviour related to health
	Mental health	– Self‐control, emotional management and expression – Internalising and externalising behaviours – Trauma symptoms – Self‐esteem – Emotional intelligence – Self‐efficacy – Motivation – Prosocial behaviour – Positive outlook – Coping
	Socioemotional functioning	– Social competencies and skills – Attachment and caregiver relationships – Adaptive behaviour – Social connections and relationships
	Cognitive functioning	– Language development – Pre‐academic skills (e.g., literacy/numeracy) – Approaches to learning – Problem‐solving skills – Academic achievement – School engagement/school attachment
Adult perpetrator/child or youth offender	Desistance	– The degree of cessation of the maltreating behaviour
	Recidivism	– The occurrence of relapse into maltreating behaviour
	Maltreatment behaviours	– Harmful coercive behaviours – Problem sexual behaviour (children under 10) – Harmful sexual behaviour (children aged from 10 up to 18‐) – Sexually offending behaviour (children aged between 10 and 18 years receiving treatment through a juvenile justice intervention)
Parent/caregiver	Behaviour/knowledge/attitudes	– Parental normative beliefs relating to institutional maltreatment related policies and practices – Parent perceptions about their child's understanding of protective behaviour concepts – Behavioural responsiveness to lack of institutional standards

#### 4.2.4 Adverse outcomes

This EGM included any measure of adverse outcomes relating to the included interventions and outcome categories. All adverse outcomes explicitly described as such in the eligible studies were included in the EGM synthesis. Unintended adverse effects may include a range of outcomes affecting victims, perpetrators or institutions.

#### 4.2.5 Visual EGM framework

Table 3 provides the EGM framework that forms the basis of the final EGM map, which incorporates the four intervention categories and six outcome categories.

**Table 3:** EGM framework

Outcome domain	Institutional safeguarding practice			Disclosure	Child safety: Maltreatment occurrence or reoccurrence				Child health and wellbeing					Adult perpetrator/child or youth offender			Parent/caregiver
Intervention category	Culture	Operations	Environment	Disclosure rates	Neglect	Emotional abuse	Physical abuse	Sexual abuse	Knowledge/awareness	Physical health	Mental health	Social‐emotional functioning	Cognitive functioning	Desistance	Recidivism	Maltreatment behaviour	Behaviour/knowledge/attitudes
Disclosure																	
Prevention																	
Response																	
Treatment																	

### 4.3 Criteria for inclusion and exclusion of studies in the EGM

#### 4.3.1 Types of participants

As outlined by the EGM scope/framework, we included studies where the study participants were:

- Children aged under 18 years at the point of baseline measurement, either living in and/or engaging in activities within institutional settings;
- Child/youth offenders or adult perpetrators of institutional child maltreatment and/or
- Adults participating in interventions that improved the professional practice of staff and organisational standards of institutions engaging with children and families.

#### 4.3.2 Types of study designs

This EGM included studies that used the following study designs: finalised and ongoing overviews of systematic reviews, systematic reviews (including scoping reviews), and primary effectiveness studies. Systematic reviews and overviews of reviews were included where they reported replicable methods to synthesise and summarise available research evidence to answer a well‐defined research question. Systematic reviews with and without meta‐analyses were included.

Given potential limitations in being able to measure institutional changes, as well as that some types of studies may not be conducive to randomisation, we included a number of study designs that meet the inclusion criteria for the Cochrane Effective Practice and Organisation of Care (EPOC, 2017).

These included:

- Randomised trials: An experimental study in which people are allocated to different interventions using methods that are random. Including head‐to‐head studies and studies with control groups not receiving the intervention. Participants may be assigned to interventions individually or by group (cluster‐randomised trials).
- Nonrandomised trial: An experimental study in which people are allocated to different interventions using methods that are not random. As per Cochrane Effective Practice and Organisation of Care recommendations, we accepted nonrandomised trials with at least two intervention sites and two control sites.
- Controlled before‐and‐after studies: A study in which observations are made before and after the implementation of an intervention, both in a group that receives the intervention and in a control group that does not. Allocation is usually determined by other factors outside the control of the investigators.

The following quasi‐experimental designs were included:

- Interrupted time series study: A study that uses observations at multiple time points before and after an intervention (the “interruption”). The design attempts to detect whether the intervention has had an effect significantly greater than any underlying trend over time. Where an interrupted time series study includes measurements made in the same individuals at each time point it is called a repeated measures study. As per Cochrane Effective Practice and Organisation of Care recommendations, accepted interrupted time series include at least three data points before and three after the intervention. We also excluded studies without a clearly defined point in time at which the intervention occurred.
- Regression discontinuity designs: A quasi‐experimental, pretest‐posttest control group design that is characterised by its unique method of assignment to intervention. Participants are assigned to either the intervention group or control group solely on the basis of a cut‐off score on a pre‐test measure. The design is so named because a regression line is plotted to relate the assignment and outcome variables. If the treatment is effective, a discontinuity in the regression line should occur at the cut‐off point. By comparison, the absence of a discontinuity is interpreted as a null effect.
- Difference of difference or other econometric designs: A quasi‐experimental design that makes use of longitudinal data from treatment and control groups to obtain an appropriate counterfactual to estimate a causal effect. It is typically used to estimate the effect of a specific intervention or treatment (such as a passage of law, enactment of policy, or largescale programme implementation) by comparing the changes in outcomes over time between a population that is enroled in a programme (the intervention group) and a population that is not (the control group).
- Propensity score matching and other matching designs: Propensity score matching creates sets of participants for treatment and control groups. A matched set consists of at least one participant in the treatment group and one in the control group with similar propensity scores. The technique attempts to estimate the effect of a treatment, policy, or other intervention by accounting for the covariates that predict receiving the treatment.

The above implies that the following study designs and methodologies were excluded from this EGM:

- Noncontrolled pre‐post evaluations
- Case studies
- Cross‐sectional studies
- Observational studies
- Opinion pieces, editorials
- Studies solely employing qualitative methods

#### 4.3.3 Study report status

This EGM included both finalised and ongoing studies. Ongoing studies were registered protocols identified from searches of electronic databases, trial registries and grey literature. No limitations were placed on the year of publication.

Studies written in the following languages were included:

- English
- German
- French
- Spanish
- Italian
- Portuguese
- Dutch
- Danish
- Swedish
- Norwegian

This restriction was due to a lack of available resources to translate studies reported in other languages.

#### 4.3.4 Types of settings

The EGM included studies conducted in low, middle and high‐income countries.

The EGM was not limited to populations at a greater risk of child maltreatment, or to populations already exposed to institutional child maltreatment. It took a whole‐of‐population approach, thereby including universal, targeted and indicated interventions (i.e., primary, secondary and tertiary approaches).

For this EGM, “institutional setting” referred to any public or private body, agency, association, club, institution, organisation or other entity or group of entities of any kind (whether incorporated or unincorporated), that also provides, or has at any time provided, activities, facilities, programs or services of any kind that provide the means through which adults have contact with children, including through their families (adapted from Australian Government, 2017).

The following is a list of examples of eligible institutional settings:

- Kindergarten/preschool/centre based early childhood education and care settings;
- Schools/before and after‐school care settings;
- Sports clubs, sport and recreation settings;
- Dance, drama and music studios/schools;
- Churches/religious institutions;
- Summer/vacation camps;
- Out‐of‐home care settings (including foster care, residential care, orphanages);
- Detention centres/juvenile justice settings;
- Rescue centres;
- Primary and secondary health care facilities and/or
- Any other type of organisation/institutional setting that met the definition above.

#### 4.3.5 Types of interventions

Interventions described within the identified studies were assessed against the EGM's intervention categories. The EGM framework included four intervention categories: prevention, disclosure, response and treatment. Table 1 presents examples of possible interventions under each category. Systematic reviews in which only a subset of studies covered interventions eligible for inclusion, were included in the map, provided that the outcome measures reported for these interventions were of relevance to this EGM.

#### 4.3.6 Outcomes of interest

This EGM included studies that reported outcome measures that could be categorised under the EGM's six outcome categories: institutional safeguarding practice, disclosure, child maltreatment occurrence/reoccurrence (child safety), child wellbeing, adult perpetrator/child or youth offender outcomes, and parent/caregiver outcomes. These have been further outlined in Table 2.

#### 4.3.7 Role of outcomes

Studies were only included if they measured outcomes within scope of the EGM framework.

### 4.4 Search methods for identification of studies

#### 4.4.1 Search sources

The full EGM search strategy is outlined in this section. No search restrictions were placed on the database searches, including year of publication, publication format or language (however, see Section 4.3.3).

##### Academic databases

The following 10 electronic databases were searched for eligible studies:

- Medline
- PsycInfo
- CINAHL
- ERIC
- Informit Families and Society Collection (Australian)
- Sociology Source Ultimate
- Sociological Abstracts
- Scopus
- The Campbell Collaboration Library
- Proquest‐Dissertations and Theses

The database search strategy and the date of the last search for each of these databases, can be found at Appendix 1.

##### Trial and systematic review registries

- PROSPERO
- ClinicalTrials.gov (US)
- ISRCTN registry (UK)
- EU Clinical Trials Register
- Australia and New Zealand clinical trial registry (ANZCTR)

##### Grey literature

Table 4 lists the grey literature sources for the EGM.

**Table 4:** Grey literature sources

Organisational websites	Grey literature databases	Inquiry reports
US Child Welfare Services	Proquest‐Dissertations & Theses	Australian Government. Royal commission into institutional responses to child sexual abuse [Australia]: Australian Government Royal Commission; 2018. Report No.: Vols 1–17. https://www.childabuseroyalcommission.gov.au
World Health Organisation		Pennsylvania Attorney General. Pennsylvania diocese victims report [Pennsylvania]: Attorney General; 2018. https://www.attorneygeneral.gov/report/
World Bank		Dressing, H., Salize, J., Dölling, D., Hermann, D., Kruse, A., Schmitt, E., Bannenberg, B., Hell, A., Voss, E., Collong, A., Horten, B., Hinner, J. (2018). Sexueller Missbrauch an Minderjährigen durch katholische Priester, Diakone und männliche Ordensangehörige im Bereich der Deutschen Bischofskonferenz—Projektbericht. Zentralinstitut für Seelische Gesundheit; Universität Heidelberg; Justus‐Liebig‐Universität Giessen. Retrieved on October 2, 2020 from: https://www.dbk.de/fileadmin/redaktion/diverse_downloads/dossiers_2018/MHG-Studie-gesamt.pdf
UNICEF		Law Commission of Canada. Restoring dignity—Responding to child abuse in Canadian Institutions [Canada]: Law Commission of Canada; 012. https://www.attorneygeneral.jus.gov.on.ca/inquiries/cornwall/en/hearings/exhibits/Peter_Jaffe/pdf/Restoring_Dignity.pdf
Australian Institute for Family Studies		Daniel B, Burgess C, Scott J. Review of child neglect in Scotland [Scotland]: Scottish Government; 2012. https://www.gov.scot/publications/review-child-neglect-scotland/
London School of Hygiene and Tropical Medicine		New Zealand House of Representatives. Inquiry into improving child health outcomes and preventing child abuse with a focus from preconception until three years of age [New Zealand]: New Zealand House of Representatives; 2014 https://www.parliament.nz/resource/en-NZ/50DBSCH_SCR6007_1/3fe7522067fdab6c601fb31fe0fd24eb6befae4a
National for Health and Care Excellence		Northern Ireland Historical Institutional Abuse Inquiry 1922–1995 [Northern Ireland]: Historical Institutional Abuse Inquiry; 2017 https://www.hiainquiry.org/historical-institutional-abuse-inquiry-report-chapters
National Society for the Prevention of Cruelty to Children		Unabhängige Kommission zur Aufarbeitung sexuellen Kindesmissbrauchs [Germany]: Aufklärung von Ausmaß, Art, Umständen, Ursachen und Folgen von sexueller Gewalt g gen Kinder und Jugendliche in Deutschland seit dem Jahr 1945; 2016–2023 https://www.aufarbeitungskommission.de
Better Care Network		

The search for grey literature was expanded based on input from multiple stakeholders (see Asking Experts). Our research team also collaborated with the team behind the Pundir et al. (2019) EGM focused on violence against children in low‐ and middle‐income countries to exchange grey literature potentially relevant to both of the EGMs.

##### Asking experts

The members of our Subject Matter Experts group (Appendix 2) were invited to (a) forward studies of potential relevance to this EGM, and (b) make their networks aware of the project and invite them to forward potentially relevant studies.

##### Systematic review searches

The included studies of all included systematic reviews and overviews of reviews underwent title/abstract and full text screening, as per other studies identified by the search strategy.

### 4.5 Data collection and analysis

#### 4.5.1 Screening and study selection

Seven reviewers took part in the whole screening and study selection process. Each title/abstract identified by the search strategy was screened against the selection criteria, by at least two reviewers working independently. The full text of studies that were deemed potentially relevant at the title/abstract screening stage were further assessed by two reviewers working independently. Any discrepancies in the decisions made by reviewers were resolved by an additional reviewer, or by discussion/consensus. Authors who were involved in any of the identified studies did not take part in the screening and selection of those studies. The Covidence platform (Covidence, 2020) was used for literature screening. No automation or text‐mining was used to identify studies.

#### 4.5.2 Data extraction, coding and data management

Five reviewers took part in data extraction, coding and data management. Information within each of the included reviews/primary studies was extracted and coded by two coders working independently. Any discrepancies in the decisions made by the first two coders were resolved by an additional reviewer or by discussion/consensus. Where information was not available from the published reports, study authors were contacted to obtain missing information. Multiple reports of the same study were collated to ensure that each study, rather than each report, was the primary unit of interest in the review (with some exceptions, see Sections 4.6.1 and 5; Table 5). Authors who were involved in any of the identified studies did not take part in data extraction/coding/critical appraisal of those studies. Before data extraction commenced, all reviewers extracted data from the same subset of articles, and this data extraction was compared. Inter‐reviewer agreement, consistency of comprehension and application were assessed, and additional training initiated where necessary. Following this, ongoing spot checks were completed on data extracted from a random sample (at least 10% in total) of studies.

**Table 5:** Studies with multiple publications included in the EGM

Study	Type of study	Associated publications
Good School Toolkit (GST)	Primary study	Devries et al. (2015)
		Devries et al. (2017)
		Devries et al. (2018)
		Knight et al. (2018)
		Merrill et al. (2018)
Bucharest Early Intervention Project (BEIP)	Primary study	Bick et al. (2015)
		Humphreys et al. (2015)
		Johnson et al. (2010)
		Smyke et al. (2010)
		Troller‐Renfree et al. (2015)
		Wade et al. (2018)
Children Need to Know: Personal Safety Training Program	Primary study	Kraizer et al. (1988)
		Fryer et al. (1987)
School‐based education programs for the prevention of child sexual abuse	Systematic review	Zwi et al. (2007)
		Walsh et al. (2015)

Data extracted from the studies included information on: the publication/study (e.g., year, first author, country undertaken), study design (e.g., randomisation, comparator groups), institutional setting, target population, type of maltreatment, intervention type and outcomes. The final version of the coding scheme, with all data items, can be found in Appendix 3. The original coding scheme was pretested with a select sample of included studies/reviews representing the range of eligible study designs. It was further refined and adjusted based on this testing, resulting in the final coding scheme (Appendix 3).

All interventions described in the primary studies were further coded following the WHO's INSPIRE categories (WHO, 2016). Including this step was a posthoc decision, added after the publication of the protocol (see Differences between protocol and review). The WHO‐INSPIRE framework identifies seven evidence‐based strategies to prevent violence against children and adolescents across health, social welfare, education, finance and justice settings. The strategies are intended to reinforce each other and work best in combination. They include (spelling INSPIRE): implementation and enforcement of laws, norms and values, safe environments, parent and caregiver support, income and economic strengthening, response and support services, and education and life skills (WHO, 2016).

#### 4.5.3 Quality appraisal

RCTs and systematic reviews were assessed for quality (i.e., the confidence we can have in the study's reported findings) using the following tools:

- The Cochrane Risk of Bias 2.0 tool for RCTs (Sterne et al., 2019)
- The AMSTAR 2 tool for systematic reviews (Shea et al., 2017)

The Cochrane Risk of Bias 2.0 tool for RCTs is designed for primary effectiveness studies using randomised study designs. It is structured into a fixed set of domains that focus on different aspects of trial design, conduct and reporting. These domains include an assessment of the potential risk of bias relating to: the process of randomisation, deviations from the intended intervention/s, missing outcome data, outcome measurement and reported results. Each domain includes a series of questions designed to gather information that allows for an assessment of the features of the trial that may contribute to the risk of bias. A judgement about the risk of bias relating to each domain is generated by an algorithm, based on answers to the questions. An overall judgement is also generated. Judgements about the potential risk of bias are grouped as: Low risk; Some Concerns, or; High risk. For this EGM, risk of bias was explored and reported for each domain, as well as for overall risk, as outlined by the tool.

The AMSTAR 2 tool is a comprehensive critical appraisal instrument for systematic reviews (Shea et al., 2017). AMSTAR 2 includes a set of questions about features of the systematic review that help to determine confidence in the reported results. An overall assessment is made based on the responses to these questions relating to the critical domains outlined by the tool. AMSTAR 2 scores are coded as critically low, low, moderate or high quality as outlined within the tool's guidelines (Shea et al., 2017). In order to present the AMSTAR2 categorisations alongside the primary study assessments in the visual EGM, studies that received a “critically low” or “low” assessment, were combined into a single “low” category. Therefore, for this EGM, the overall assessments were:

- High: The systematic review provides an accurate and comprehensive summary of the results of the available studies that address the question of interest.
- Moderate—The systematic review has more than one weakness, but no critical flaws. It may provide an accurate summary of the results of the available studies that were included in the review.
- Low/Critically Low—The review has one or more critical flaw and may not provide an accurate and comprehensive summary of the available studies that address the question of interest.

Five reviewers were involved in assessing the quality of the included studies. Two reviewers worked independently to assess each study, and any discrepancies were cleared via consensus or by an additional member of the review team working independently. Only RCTs were assessed for their risk of bias; neither protocols, nor primary studies which were not RCTs, were assessed.

### 4.6 Analysis and presentation

#### 4.6.1 Unit of analysis

Each entry in this EGM is either an overview of systematic reviews, a systematic review or a primary study. Where a single study is associated with multiple reports/publications, these have been presented as a single study when the reported characteristics are the same (e.g., participants, maltreatment type, institutional setting), and presented separately within the EGM when the reported characteristics differ (e.g., outcome measures) (see Table 5). Each publication was critically appraised separately.

#### 4.6.2 Planned synthesis

The visual EGM has been supplemented by a narrative synthesis of the included studies, which encompasses a descriptive summary of the number of studies included in the EGM, and their distribution across different coding categories such as study type, geography, maltreatment type, target populations, interventions and outcomes. This narrative synthesis also discusses the potential use of the EGM and highlights its boundaries and limitations.

#### 4.6.3 Visual mapping of the EGM

The visual EGM was developed using the R Project for Statistical Computing (R Core Team, 2019). Bespoke code was developed by the Centre for Evidence and Implementation that utilised the ggplot2 package for visualisation (Wickham, 2016). As per the EGM framework, the included studies are mapped in a table in which the rows are the intervention categories, and the columns the outcome categories. A single study can appear in several cells on the map if it reported on more than one intervention category and/or more than one outcome domain. Study quality was highlighted by colour, using the traffic light system (i.e., low risk of bias/high‐quality = green, some concerns of bias/moderate‐quality = yellow, high risk of bias/low‐quality = red).

### 4.7 Stakeholder engagement

This EGM was developed in close collaboration between:

- Porticus, who funded the study, represented by: Jane Leek, Regional Director, Porticus UK and Dr. Joachim Krapels, Senior Analyst, Porticus Effective Philanthropy Group.
- Giving Evidence, represented by its CEO Caroline Fiennes.
- The Centre for Evidence and Implementation, represented by: Executive Director, Dr. Robyn Mildon; Director, Dr. Bianca Albers; and Senior Advisor, Dr. Meghan Finch (past).
- Monash University, represented by: Prof. Aron Shlonsky and Research Fellow, Dr. Rebecca Featherston

All stakeholder representatives are included as coauthors on the published EGM Campbell Collaboration Protocol. More information about each authors' expertise relevant to this review can be found in Contributions of Authors.

In addition, subject matter experts representing 16 different organisations concerned with safeguarding practice and research were convened for the production of this EGM to ensure that all relevant aspects of child maltreatment within institutional settings were sufficiently captured. The group was initially gathered for a general information and engagement meeting. Each member of this group was then invited to submit relevant publications to be considered for the EGM, which were reviewed as per all other studies identified by the search strategy. The subject matter experts will be further involved in disseminating the final EGM among relevant organisations, institutions and networks around the world. The composition of this group can be found in Appendix 2.

## 5 RESULTS

### 5.1 Included studies

**Figure 1:**
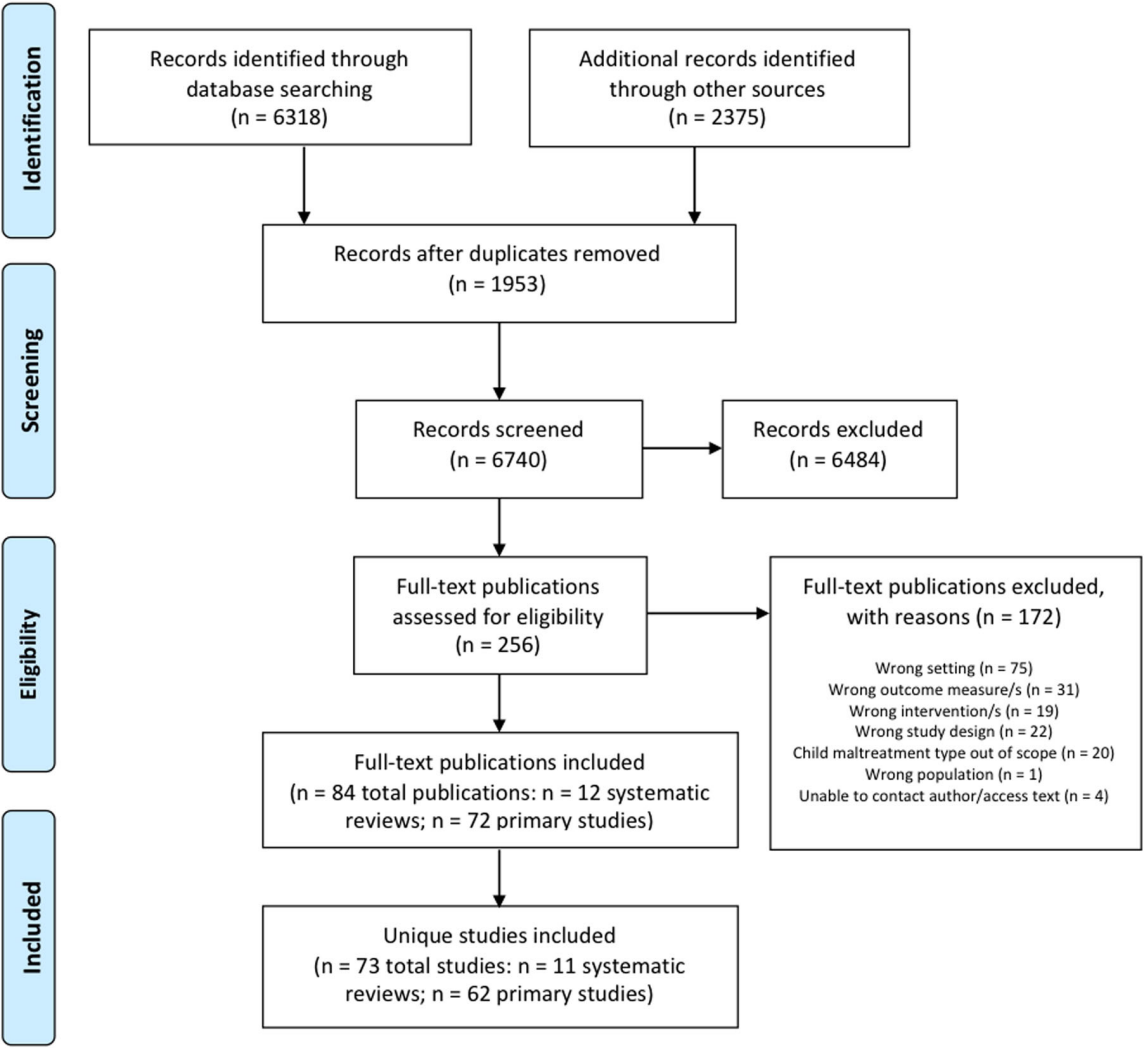
PRISMA flow diagram presenting the flow of studies identified by the search strategy, screened and included in the EGM. PRISMA, Preferred Reporting Items for Systematic Reviews and Meta‐Analyses

The search strategy identified 73 studies (across 84 publications): 62 primary effectiveness studies and 11 systematic reviews. Figure 1 shows the flow of studies that were identified from the search strategy, screened and finally included in the EGM. Appendix 4 provides a list of excluded studies and their primary reason for being excluded.

The academic electronic search strategy yielded 6318 citations, and an additional 2375 records from other sources were identified. After removing duplicates and screening titles and abstracts, 256 studies remained for full‐text review. A total of 84 eligible publications of studies were identified after full‐text review (including systematic reviews, publications describing primary studies and protocols for primary studies). Appendix 5 provides details of the source (where the publication was found) of each included publication.

Of the total 84 included publications, 12 were completed systematic reviews. Three of these were scoping reviews that met our systematic review criteria. Two reviews were related: Walsh et al. (2015) was an update of an earlier Cochrane review published in 2007 (Zwi et al., 2007). While both are included in the EGM, where the reported characteristics are identical for each (e.g., maltreatment type, institutional setting, target population), they have been represented collectively (i.e., counted as a single study). Where the reported characteristics differ (e.g., included age groups), they have been represented separately (i.e., counted as two separate studies). Table 5 provides a summary.

Seventy‐two publications of primary effectiveness studies (hereafter referred to as “primary studies”) were identified: 69 were completed studies, and three were ongoing (described in a published protocol where results had not yet been generated). Among these, five publications related to a study evaluating the Good School Toolkit (GST), and reported different outcomes from the same sample or a subset of the same sample (Devries et al., 2015, 2017, 2018; Knight et al., 2018; Merrill et al., 2018). Six publications reported results of the Bucharest Early Intervention Project (BEIP; Bick et al., 2015; Humphreys et al., 2015; Johnson et al., 2010; Smyke et al., 2010; Troller‐Renfree et al., 2015; Wade et al., 2018). These publications reported the same or different outcomes at various follow‐up points from the same sample of children originally randomised for the BEIP. Two further publications reported results from a school‐based prevention programme (Fryer et al., 1987; Kraizer et al., 1988). Though all of the publications reporting on these three studies are included in the EGM, where the reported characteristics are the same for more than one study (i.e., study design, maltreatment type, institutional setting, target population, country), they have been represented as a single study (i.e., the multiple publications are counted as a single study). Where the outcomes reported across the papers were different, the individual publications have been reported separately (i.e., counted as two separate studies). Table 5 provides a summary.

### 5.2 Visual EGM

**Figure 2:**
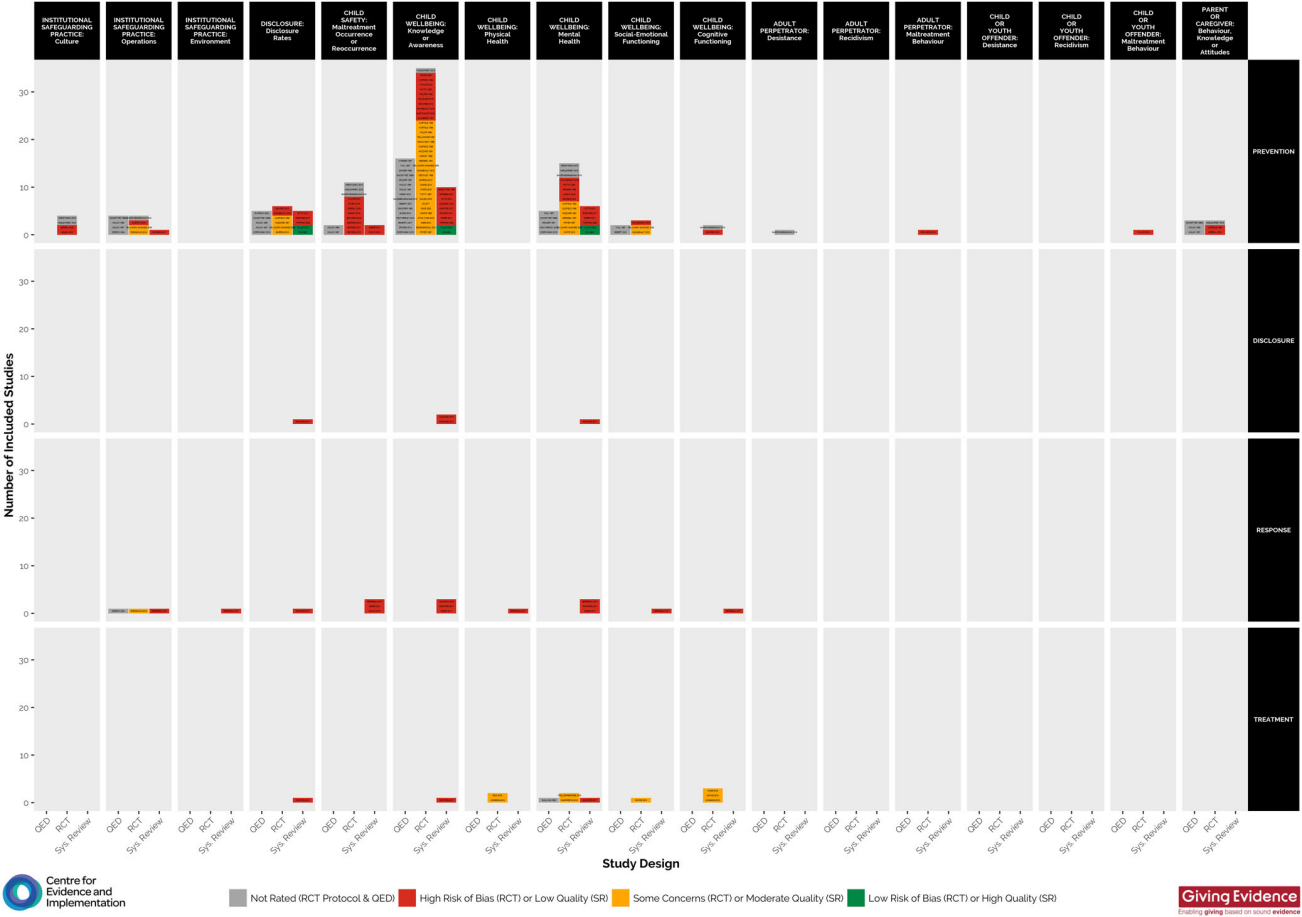
Evidence and gap map of included studies, presenting key intervention categories, outcome categories and study quality/risk of bias

Figure 2 shows the visual EGM based on the EGM framework. The cells within the map show the number of studies for each study type (RCT, quasi‐experimental and systematic review). First author name and publication year are shown for each study. Study quality is highlighted by colour, using the traffic light system (i.e., low risk of bias/high‐quality = green; some concerns of bias/moderate‐quality = yellow; high risk of bias/low‐quality = red).

**Figure 3:**
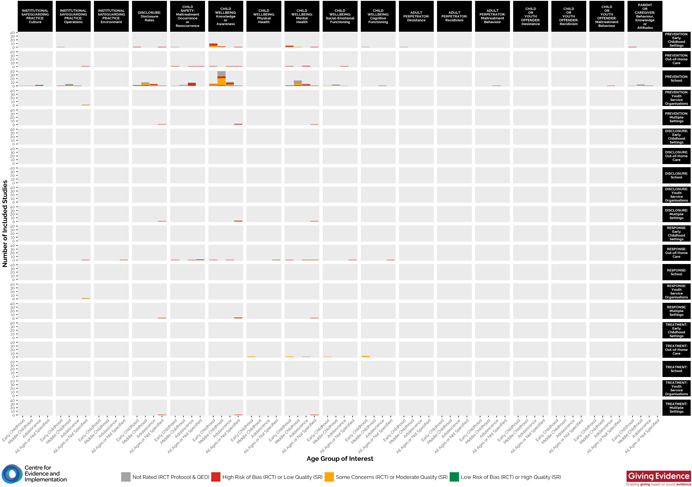
Evidence and gap map of included studies, presenting institutional settings, key intervention categories, outcome categories and study quality/risk of bias

Figure 3 shows a second visual EGM, which has been included to further highlight the institutional settings addressed by the identified studies. The cells within the map show studies for each child age group (early childhood, middle childhood, adolescence, all ages/not specified) and institutional settings represented by the studies (early childhood settings, out‐of‐home care, school, youth services organisations, multiple settings). Study quality is also highlighted in this map using the traffic light system (i.e., low risk of bias/high‐quality = green; some concerns of bias/moderate‐quality = yellow; high risk of bias/low‐quality = red).

### 5.3 Characteristics of the included studies

Appendix 6 details the characteristics of the included primary studies. Appendix 7 details the characteristics of the included systematic reviews.

#### 5.3.1 Study status

The vast majority of the 62 primary studies (*n* = 59) were completed. Three were described in published protocols and coded as ongoing (Baker‐Henningham et al., 2016; McElearney et al., 2018; Ssenyonga et al., 2018). No ongoing systematic reviews were identified.

#### 5.3.2 Study design

Of the 62 completed and ongoing primary studies, 42 were RCTs and 20 used a quasi‐experimental design.

#### 5.3.3 Study language

One primary study was published in German (Feldmann et al., 2018) and one primary study was in Spanish (del Campo Sánchez & Sánchez, 2006). The remaining studies were published in English.

#### 5.3.4 Publication year

**Figure 4:**
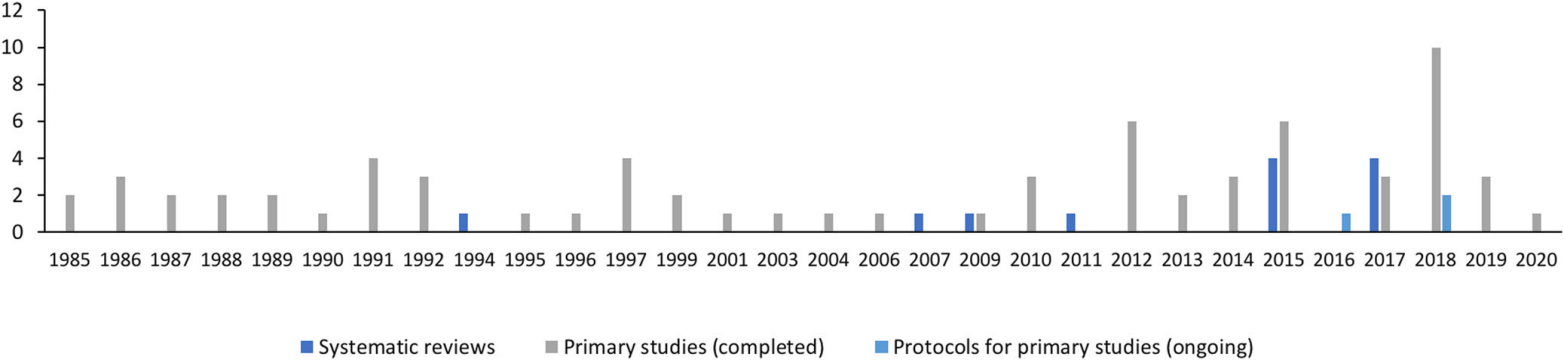
Included publications by publication year (*N* = 84)

Figure 4 details the number of included studies (including each published report) published each year. The earliest primary study included in the EGM was published in 1985. No more than four studies (range: 1–4 studies) were published per year before or during 2011. There is then a marked increase in the amount of activity. Of the total studies, 54% were published between 2012 and 2020, with the peak number of completed primary studies published in 2018 (*n* = 10). The first systematic review was published in 1994, nine years after the first primary study was published, with the four most recent reviews published in 2017.

#### 5.3.5 Geographical distribution

##### Country

**Figure 5:**
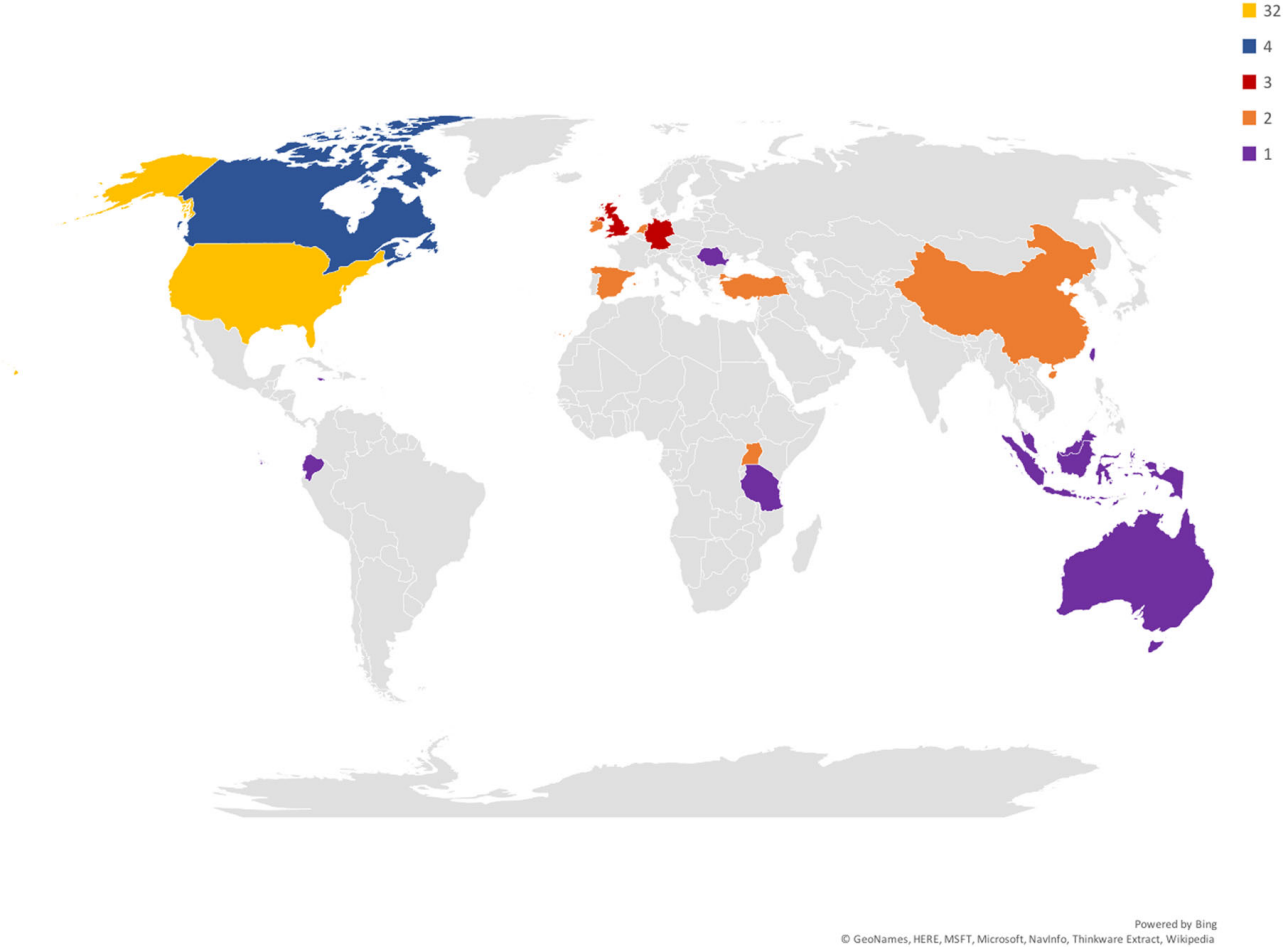
Geographical distribution of studies. Colours represent the number of studies produced by a country. For example, all countries that produced three studies have been shaded in red

Figure 5 shows the geographical distribution by country of the included primary studies (i.e., where the study actually took place). Just over half of the primary studies were conducted in the United States (*n* = 32). Canada produced four studies, three studies each came from Germany and the UK (one from Northern Ireland, two from Scotland), six countries produced two studies each (Turkey, Ireland, China, Spain, The Netherlands, Uganda) and a further eight countries contributed one study each (Australia, Ecuador, Indonesia, Jamaica, Malaysia, Romania,Taiwan, Tanzania).

##### WHO regions

Regionally, the overwhelming proportion of primary studies were conducted in the Americas (61%) and Europe (24%). The remainder were conducted in South East Asia (8%), Africa (4.5%) and the Western Pacific (1.5%). No studies were conducted in the Eastern Mediterranean region.

#### 5.3.6 Types of institutional setting

**Figure 6:**
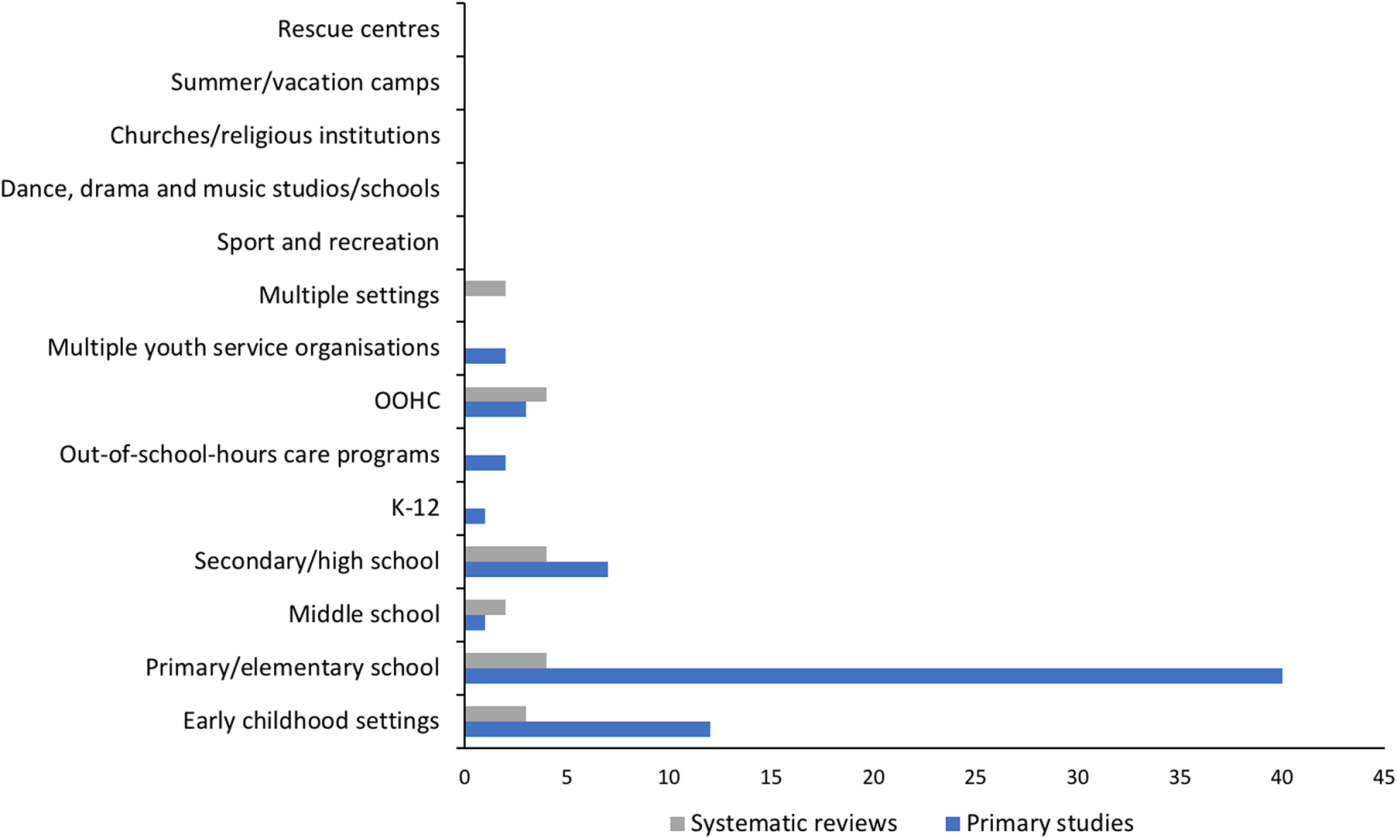
Number of included studies reporting each type of institutional setting (*N* = 73)

Figure 6 shows the number of included studies that reported on each institutional setting type.

##### Primary studies

Most primary studies (*n* = 48) were conducted in school settings, including: primary/elementary school (*n* = 40), middle school (*n* = 1), secondary/high school (*n* = 7), kindergarten to year 12 (K‐12) educational settings (i.e., schools inclusive of all years) (*n* = 1) and out‐of‐school‐hours care programmes (*n* = 2). Two studies included more than one of these school settings (Barron & Topping, 2013; Ssenyonga et al., 2018). Eleven primary studies were conducted in early childhood settings (e.g., kindergarten, preschool, day‐care), and three of these also included primary/elementary school settings (Fryer et al., 1987; Kraizer et al., 1988; Kraizer, 1991; Wurtele et al., 1986). Two primary studies were conducted across multiple settings, which included: health, school and social services agencies who respond to child maltreatment (Cerezo & Pons‐Salvador, 2004), and organisations delivering services that children access or attend (e.g., schools, day‐care, church organisations) (Rheingold et al., 2014). Three were conducted in out‐of‐home care, including foster care and orphanages (the BEIP study, and associated publications), a residential school for the deaf (Sullivan et al., 1992), and group homes (Van Lieshout et al., 2019). No studies were identified where the primary setting was sports clubs, religious organisations, summer camps, detention centres, rescue centres or primary and secondary health care facilities.

##### Systematic reviews

Most of the 11 unique systematic reviews reported on studies conducted exclusively in school and/or early childhood settings (i.e., kindergartens, preschool, day‐care) (*n* = 5). Of these, one systematic review (plus, one update) included studies reporting both primary/elementary and secondary/high school settings (Walsh et al., 2015; Zwi et al., 2007), one systematic review included early childhood and primary/elementary settings (Heidotting, 1996), one included middle and secondary/high school settings (Ricardo et al., 2011), one systematic review included several settings (across early childhood, primary/elementary and secondary/high school) (Topping & Barron, 2009), and one included only early childhood settings (Pitts, 2015). Of the remaining five systematic reviews, four focused on residential care (e.g., orphanages, out‐of‐home care) (Hermenau et al., 2017; McKibbin, 2017; Sherr et al., 2017; South et al., 2015). Two systematic reviews included studies conducted across various settings (Quadara et al., 2015; Radford et al. 2017), including school and early childhood settings, voluntary and faith‐based organisations, and sports clubs (coded as “multiple settings”).

#### 5.3.7 Target population

##### Primary studies

Among the completed and ongoing primary studies, most evaluated interventions for children in organisations (*n* = 45). Six studies assessed interventions solely for institutional staff and/or adult care providers (e.g., teachers, after‐school‐hours care staff, daycare staff, youth service organisation staff and health and social services agency staff) (Baker‐Henningham et al., 2016; Cerezo & Pons‐Salvador, 2004; Gushwa et al., 2018; Nkuba et al., 2018; Rheingold et al., 2014; Ssenyonga et al., 2018). Nine studies assessed interventions for both children and institutional care staff and/or adult care providers (GST study; Baker et al., 2012; del Campo Sánchez & Sánchez, 2006; Edwards et al., 2019; Kolko et al., 1987, 1989; Kraizer, 1991; MacIntyre & Carr, 1999a; Taal & Edelaar, 1997).

##### Systematic reviews

Most reviews (*n* = 7) examined interventions solely for children, one included interventions targeting only institutional staff and/or adult care providers (e.g., teachers) (Hermenau et al., 2017), and four reviews included studies assessing interventions for either or both of these populations (Quadara et al., 2015; Radford et al. 2017; Sherr et al., 2017; South et al., 2015).

#### 5.3.8 Child age groups

**Figure 7:**
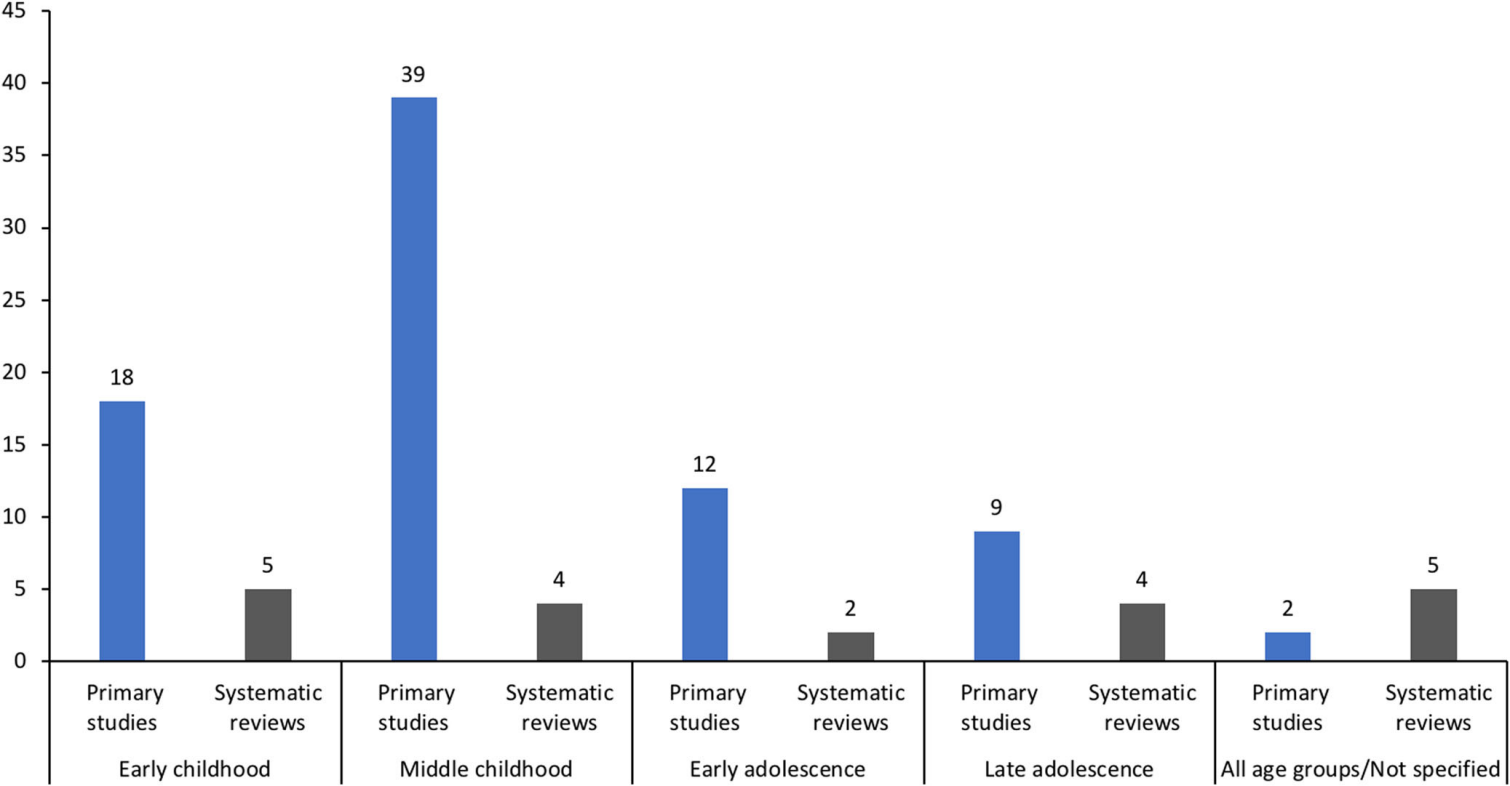
Number of included studies targeting each child age group (*N* = 73)

Figure 7 details the age group/s of the child population who received the interventions reported in primary studies, and reported by the primary studies included in the systematic reviews. Several studies included more than a single age group.

##### Primary studies

Most (*n* = 29) primary studies focused on middle childhood (6–11 years). Fewer focused on early childhood (0–5 years) (*n* = 16), early adolescence (12–14 years) (*n* = 12), or late adolescence (15–17 years) (*n* = 9). Seventeen of the primary studies included children from more than one age group (e.g., both early childhood and middle childhood aged participants), including two studies reporting on participants aged 0–18 years (Cerezo & Pons‐Salvador, 2004; Rheingold et al., 2014).

##### Systematic reviews

Most systematic reviews included studies reporting on participants in early (0–5 years) and/or middle (6–11 years) childhood (*n* = 6). Fewer systematic reviews included studies reporting on participants in early adolescence (12–14 years) and/or late adolescence (15–17 years) (*n* = 4). Most of the reviews included studies from more than one age group (*n* = 8), four additional reviews included or reported on children of all ages, between 0 and 18 years (Hermenau et al., 2017; McKibbin, 2017; Quadara et al., 2015; Radford et al. 2017; South et al., 2015 did not specify age).

#### 5.3.9 Child risk status

##### Primary studies

Most primary studies focused on children not at particular risk of maltreatment (*n* = 57). That is, the approach was universal rather than targeted to specific groups known to be at greater risk. All interventions offered to universal populations were prevention‐focused and delivered in educational settings. Two of these studies reported disclosure rates for a subset of children later suspected of experiencing abuse who had at some point in the past taken part in the universal intervention under evaluation (Elfreich et al., 2020; MacIntyre & Carr, 1999b). The GST was also a school‐based prevention intervention delivered to the whole school, however because the children in it reported violence in the past week, these studies were coded as targeting children at‐risk and/or exposed to violence (as opposed to being coded as a universal intervention). Two further primary studies included children at increased risk, including special education high school students with cognitive and/or physical disabilities (Dryden et al., 2014) and boys in residential youth care (Van Lieshout et al., 2019). Two focused on children exposed to maltreatment, including children raised in orphanages who experienced extreme neglect in early life (BEIP study) and children sexually abused at a residential school for the deaf (Sullivan et al., 1992). All studies ultimately had a focus on children, even where the intervention was delivered solely to institutional staff.

##### Systematic reviews

Most systematic reviews included primary studies focused on child populations that were not at a higher risk of maltreatment than the general population (i.e., universal child populations) (*n* = 7). Four systematic reviews included studies assessing exposed populations, three of which included child participants in out‐of‐home care (e.g., residential care, orphanage, foster care) (Hermenau et al., 2017; Sherr et al., 2017; South et al., 2015) and one included interventions providing support for victims and survivors of child sexual abuse (Radford et al. 2017). One review included studies that focused on children at a higher risk of maltreatment living in out‐of‐home care (McKibbin, 2017).

#### 5.3.10 Types of maltreatment

**Figure 8:**
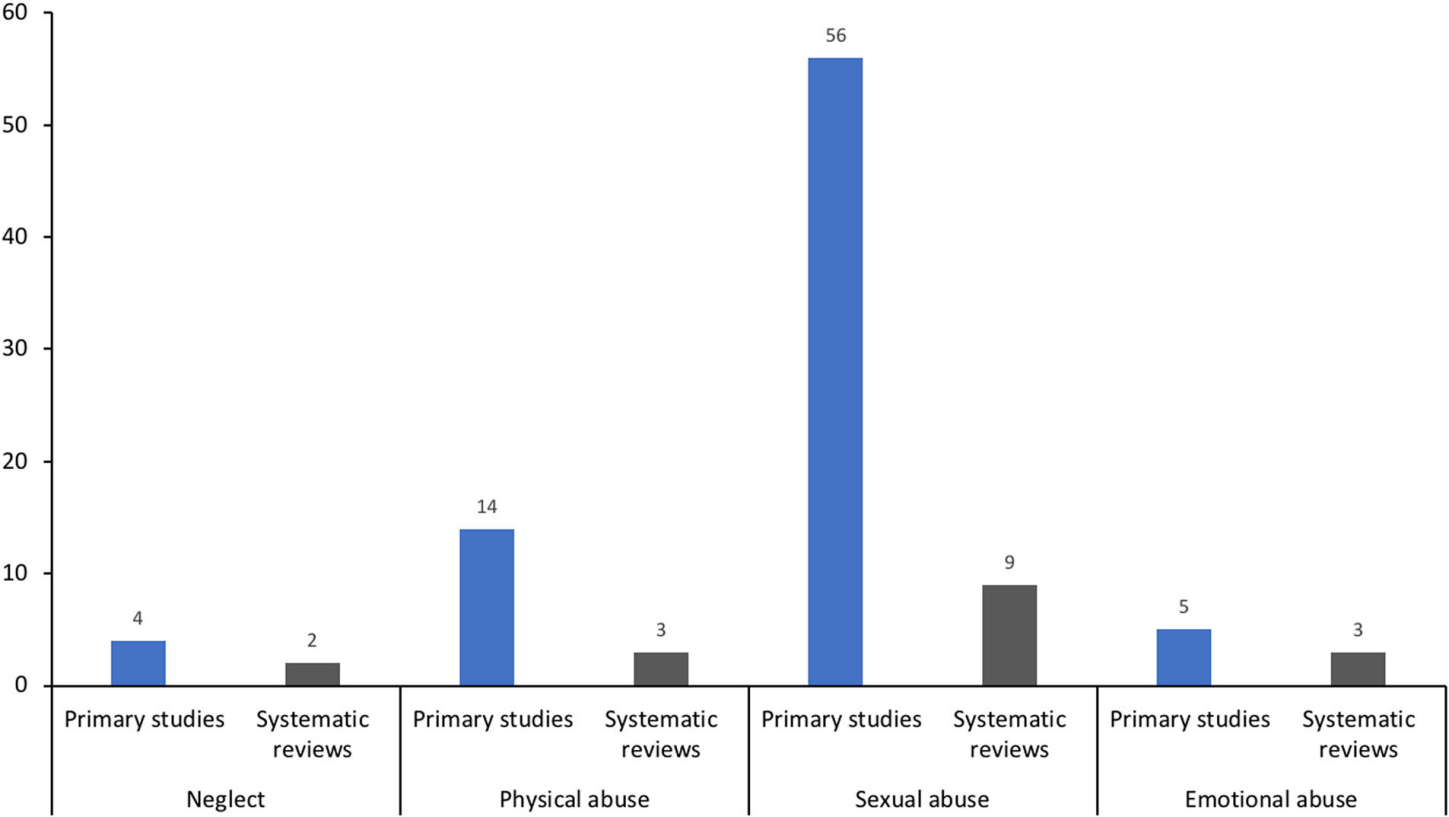
Number of included studies reporting each type of maltreatment (*N *= 73)

Figure 8 details the number of included studies targeting each type of maltreatment.

##### Primary studies

Most completed and ongoing primary studies included interventions that had a singular focus on sexual abuse (*n* = 46), with 10 additional studies focussing on sexual abuse alongside other maltreatment types (total sexual abuse: *n* = 57). Four primary studies assessed interventions specifically addressing physical abuse, and a further 10 incorporated physical abuse alongside other maltreatment types (total physical abuse: *n* = 14). Child neglect was the primary focus of the BEIP study, and three other studies also addressed neglect alongside other maltreatment types (total neglect: *n* = 4). No study focussed exclusively on emotional abuse, but emotional abuse was considered in the GST study and four others (total emotional abuse: *n* = 5).

##### Systematic reviews

Of the 11 systematic reviews, eight (plus one update) included studies that reported on interventions relating solely to sexual abuse. The other three systematic reviews included primary studies that reported on one or more types of child maltreatment. Hermenau et al. (2017) and Sherr et al. (2017) included studies assessing physical and emotional abuse, as well as neglect, and Ricardo et al. (2011) included studies reporting on sexual, physical and emotional abuse.

### 5.4 Quality appraisal

#### 5.4.1 Primary studies

**Figure 9:**
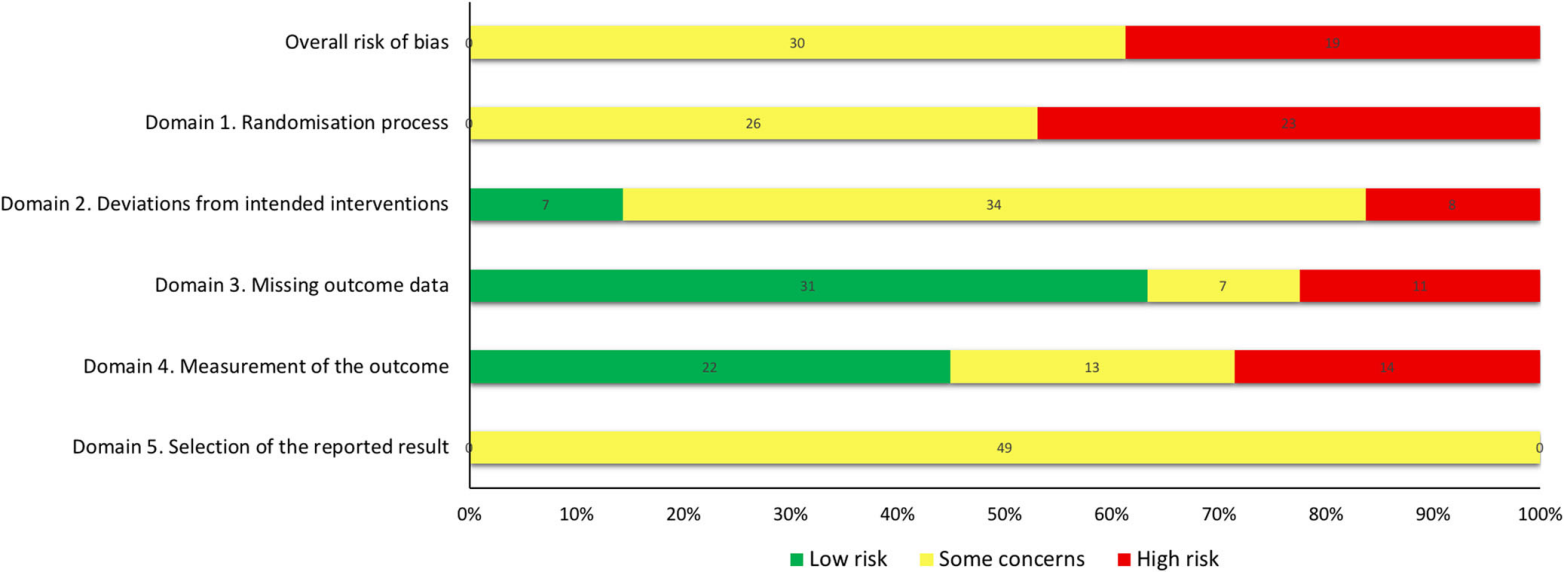
Number of completed RCT studies (*N *= 49) assessed as low, some concerns, or high risk of bias, overall and by domain using the Cochrane Risk of Bias Tool 2.0

Figure 9 shows the number of completed RCT studies that were assessed as low, some concerns or at a high risk of bias, both overall and by domain (see Appendix 8 for individual study assessments). Of the 49 reports of completed RCTs (noting that the BEIP study, GST study, Fryer et al., 1987 and Kraizer et al., 1988 publications were assessed separately), all were assessed to have either a “high risk” of bias (*n* = 18) or “some concerns” (*n* = 31) using the Cochrane Risk of Bias 2 tool (Sterne et al., 2019). No study received an overall assessment of low risk. Most studies raised concerns in relation to the randomisation process (*n* = 26), often because insufficient information about the randomisation method was provided to allow for high confidence in it. Of the studies assessed, 33 received an assessment of “some concerns” for items concerning deviations from the intended intervention. Thirty‐one of the 49 RCTs were assessed as low risk for potential biases associated with missing outcome data: this was generally because few participants dropped out of these studies. On how the outcomes were measured, 22 RCTs were at a low risk of bias, with the remainder raising “some concerns” or “high risk” of bias. Almost all studies received an assessment of “some concerns” in relation to the selection of reported results, with one study being at a high risk of bias for this domain.

#### 5.4.2 Systematic reviews

Overall, most systematic reviews (*n* = 10) were assessed as being of low quality (i.e., low confidence in the reported results) using the AMSTAR 2 checklist (Heidotting, 1996; Hermenau et al., 2017; McKibbin, 2017; Pitts, 2015; Quadara et al., 2015; Radford et al. 2017; Ricardo et al., 2011; Sherr et al., 2017; South et al., 2015; Topping & Barron, 2009). Two received a high quality rating (i.e., high confidence in the reported results) (Zwi et al., 2007; and update Walsh et al., 2015; assessed separately due to some variation in reported methods).

### 5.5 Interventions

**Figure 10:**
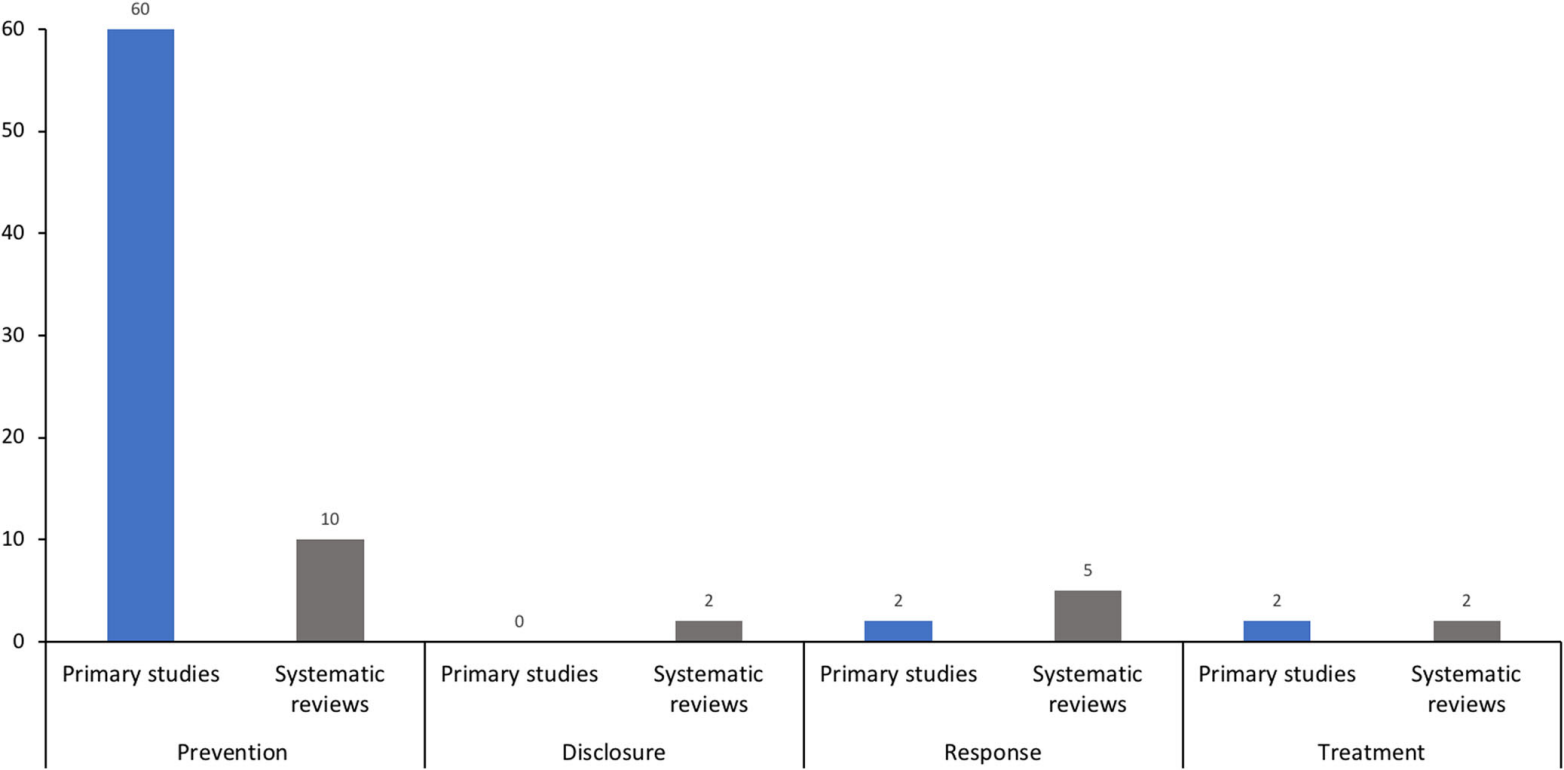
Number of included studies that reported on each of the intervention categories (*N *= 73)

Figure 10 shows the number of included studies reporting on each of the intervention categories. Interventions were categorised as prevention, disclosure, response, or treatment approaches. The studies reporting on each of the intervention categories are discussed in further detail in the sections below.

The overwhelming majority of studies assessed the effectiveness of prevention interventions (*n* = 58 primary studies; *n* = 5 systematic reviews), and a smaller number included prevention approaches alongside other intervention types (*n* = 2 primary studies; *n* = 5 systematic reviews). No primary studies were evaluations of interventions aiming solely to facilitate disclosure of child maltreatment. However, several primary studies did report on outcomes relating to disclosure (see Section 5.6). Two reviews searched for primary studies assessing interventions aiming to increase disclosure (Quadara et al., 2015; Radford et al. 2017). Note that these reviews either did not identify primary studies, or did not identify primary studies that met our inclusion criteria for disclosure interventions. Response interventions were evaluated by fewer studies (*n* = 2 primary studies; *n* = 5 systematic reviews), and for all bar one systematic review (Hermenau et al., 2017), these were reported alongside, or combined with, prevention‐focused interventions. Treatment interventions were assessed by fewer studies still (*n* = 2 primary studies; *n* = 2 systematic reviews).

#### 5.5.1 Prevention

Prevention interventions were defined as any intervention where the primary aim was to decrease the likelihood or risk of child maltreatment occurring or recurring in the future. This encompassed universal interventions for any child or adult, as well as interventions aimed at specific populations. Examples of the types of prevention interventions that could be included were school‐based safety programmes, organisational guidelines/practices and interventions to reduce perpetrator reoffending (see Table 1).

##### Primary studies

We identified 60 primary studies reporting evaluations of interventions aimed to prevent child maltreatment, including three ongoing studies. These are summarised in Appendix 6. Most of these studies were undertaken in the United States (*n* = 31); four were from Canada; three from Germany and the UK (two from Scotland, one from Northern Ireland); two each from China, Ireland, The Netherlands, Spain, Turkey and Uganda; and one each from Australia, Ecuador, Indonesia, Jamaica, Malaysia, Taiwan and Tanzania. Most were RCTs, including: *n* = 3 ongoing studies, *n* = 37 completed studies and *n* = 43 reports of completed studies (this number includes the GST publications, Fryer et al., 1987 and Kraizer et al., 1988, counted separately). Eighteen primary studies were assessed as having a high risk of bias, with the remaining 25 assessed as raising “some concerns” relating to the risk of bias. Most interventions were delivered in schools (*n* = 48), with fewer solely or also delivered in kindergarten/preschool/daycare settings (*n* = 13). Two included after‐school‐hours care programmes, two interventions were delivered across youth service organisations (Cerezo & Pons‐Salvador, 2004; Rheingold et al., 2014), and one was delivered in residential care (a group home) (Van Lieshout et al., 2019).

The most frequently targeted age group was middle childhood (6–11 years) (*n* = 39), followed by early childhood (*n* = 17) and early adolescence (*n* = 15). Fewer prevention interventions targeted children in late adolescence (15–17 years) (*n* = 8). Some interventions targeted more than one age group, including one study which targeted children across the range of age groups, from 0‐18 years.

In relation to maltreatment type, most interventions aimed to prevent sexual abuse (*n* = 56), either as a primary focus (*n* = 45), or in combination with other forms of maltreatment (*n* = 11) (Barron & Topping, 2013; Cerezo & Pons‐Salvador, 2004; Daigneault et al., 2012; Dake et al., 2003; Dhooper & Schneider, 1995; Elfreich et al., 2020; GST; Edwards et al., 2019; Kraizer, 1991; McElearney et al., 2018; Wolfe et al., 1986). Fewer focused solely on physical abuse, either in isolation (*n* = 4) (Baker‐Henningham et al., 2016; Dryden et al., 2014; Nkuba et al., 2018; Ssenyonga et al., 2018) or in combination with other forms of maltreatment (*n* = 10) (Barron & Topping, 2013; Cerezo & Pons‐Salvador, 2004; Daigneault et al., 2012; Dake et al., 2003; Dhooper & Schneider, 1995; GST, Edwards et al., 2019, Kraizer, 1991; Wolfe et al., 1986). No prevention interventions focused solely on neglect or emotional abuse, however these maltreatment types were the focus of six interventions which also addressed other maltreatment types (neglect—Cerezo & Pons‐Salvador, 2004; Dake et al., 2003; McElearney et al., 2018; emotional abuse—Barron & Topping, 2013; Cerezo & Pons‐Salvador, 2004; Dake et al., 2003; GST; Kraizer, 1991).

Most interventions were delivered in an educational setting and were curriculum‐based, with a focus on increasing child awareness and understanding of sexual abuse and teaching self‐protection skills (*n* = 54). For most (*n* = 43), the main intervention involved workshops or lessons, alongside written, audio‐visual or other resources (e.g., films or plays, images, activity books, parent resources), and was delivered directly to children in groups via an external agency or existing trained institutional staff or students. The intensity of these sessions varied from brief standalone educational programs involving single sessions (Daigneault et al., 2012; Pulido et al., 2015); delivery of between two to eight lessons over the course of 1–2 weeks (Cecen‐Erogul & Hasirci, 2013; Conte, 1985; Dake et al., 2003; Fryer et al., 1987; Jin et al., 2017; White et al., 2018; Wurtele, Gillispie, et al., 1992), and more intense delivery with multiple lessons delivered over longer periods ranging from 5 to 10 weeks (Citak Tunc et al., 2018; Dryden et al., 2014; Taylor et al., 2010; Van Lieshout et al., 2019; Weatherley et al., 2012). One additional study assessed a school‐based rape prevention intervention consisting of three 45‐min sessions (Hillenbrand‐Gunn et al., 2010).

Twelve studies reported on interventions that aimed to improve the knowledge, attitudes and practices of the organisation's staff via training—some with and some without follow‐up support in educational settings (*n* = 9) and multiple youth service organisations (*n* = 2). Eight were RCTs, three with a high risk of bias (Gushwa et al., 2018; Merrill et al., 2018; Nkuba et al., 2018) and two raising some concerns (del Campo Sánchez & Sánchez, 2006; Rheingold et al., 2014). Three studies were ongoing (Baker‐Henningham et al., 2016; McElearney et al., 2018; Ssenyonga et al., 2018). Four used quasi‐experimental designs (Cerezo & Pons‐Salvador, 2004; Kolko et al., 1987, 1989; MacIntyre & Carr, 1999b). Among these interventions, training for staff ranged from a brief 1‐h session (Gushwa et al., 2018), up to 5 days (Ssenyonga et al., 2018), with inclusion of follow‐up support strategies such as in‐school coaching (Baker‐Henningham et al., 2016; Dryden et al., 2014), performance feedback and text messaging (Baker‐Henningham et al., 2016), and supervision and peer networks (Ssenyonga et al., 2018). All staff training interventions with follow‐up support were focused on reducing violent discipline and improving teacher–student relationships in educational settings (including school and daycare).

Four school‐based prevention interventions used more comprehensive approaches, seeking to embed the programme across the broader school community, and included multiple strategies (combined with curriculum approaches) delivered over a longer timeframe (from two terms, up to a year) (Baker‐Henningham et al., 2016; GST study; McElearney et al., 2018; Ratto & Bogat, 1990). All were RCTs, two assessed at high risk of bias (Devries et al., 2015, 2017, 2018; Knight et al., 2018; Merrill et al., 2018; Ratto & Bogat, 1990) and two were ongoing (Baker‐Henningham et al., 2016; McElearney et al., 2018). For example, the GST was aimed multiple levels within the schools including head teachers, administration, classroom teachers, and students with multilayered training, processes, and school‐led activities for each level.

Two prevention interventions involved online or web‐based delivery. Both interventions were for staff in institutional settings and were RCTs. One raised some concerns of risk of bias (Rheingold et al., 2014) and one was rated as having a high risk of bias (Gushwa et al., 2018). Gushwa et al. (2018) described a 1‐h interactive online course targeting teachers in schools inclusive of kindergarten to year 12 (where learners could choose to take the course in one session or in separate 20‐min segments). The course addressed signs and symptoms of child sexual assault, grooming, sexual misconduct behaviours, and reporting responsibilities and requirements (Gushwa et al., 2018). The second study conducted by Rheingold et al. (2014) and colleagues included delivery of a 2.5 h interactive web‐based training session (with in‐person training as a comparison) to staff from youth serving organisations (including daycare centres, church organisations and schools) focused on preventing, recognising and responding to child sexual abuse (Rheingold et al., 2014).

##### Systematic reviews

Ten systematic reviews (plus one update) included studies reporting on prevention interventions. These are described in Appendix 7. One review was assessed as being of high quality (Zwi et al., 2007; and update Walsh et al., 2015), and the remaining systematic reviews assessed as low quality (Heidotting, 1996; McKibbin, 2017; Pitts, 2015; Quadara et al., 2015; Radford et al. 2017; Ricardo et al., 2011; Sherr et al., 2017; South et al., 2015; Topping & Barron, 2009). Most systematic reviews included studies that evaluated programmes in educational settings (e.g., schools, early childhood settings) to prevent sexual abuse, either as sole focus or reported alongside studies assessing other intervention types (*n* = 7) (Heidotting, 1996; Pitts, 2015; Quadara et al., 2015; Radford et al. 2017; Ricardo et al., 2011; Topping & Barron, 2009; Walsh et al., 2015; Zwi et al., 2007). The remaining three reviews (McKibbin, 2017; Sherr et al., 2017; South et al., 2015), included prevention interventions delivered in out‐of‐home care.

Walsh et al. (2015) (an update of Zwi et al., 2007) identified 24 RCTs and QEDs evaluating school‐based education interventions for preventing child sexual abuse. Interventions were delivered to children, who were provided with age‐appropriate information relating to sexual abuse, sexual abuse prevention concepts, and/or taught self‐protective skills. The duration of these interventions ranged from a single 45‐min session to eight 20‐min sessions on consecutive days. Most interventions were brief (<90 min total duration) with some of longer duration (lasting from 90 to 180 min). All programmes were delivered on school premises and during school hours, apart from one study in which the programme was delivered in the morning before school (Walsh et al., 2015). Three other reviews also synthesised the available evidence on school‐based education interventions for the prevention of child sexual abuse (Heidotting, 1996; Pitts, 2015; Topping & Barron, 2009), assessing their impact on child knowledge and protective skills.

Ricardo et al. (2011) had a slightly different focus, examining interventions for preventing boys' and youths' use of sexual violence in community and school settings. This review included studies with randomised or quasi‐experimental designs, and reported that the vast majority (*n* = 55) used group education methods to deliver the intervention, often using existing curricula (Ricardo et al., 2011). One‐third of included interventions were one session, 14 interventions were conducted in 2–9 sessions, and 12 were conducted in 10–15 sessions. Session durations ranged from around 1–4.5 h, with most lasting approximately 1 h. Interventions conducted as media or education campaigns lasting from a few weeks to several years were also identified. Most of the interventions were delivered by teachers (*n* = 17) (Ricardo et al., 2011).

Radford et al. (2017) and Quadara et al. (2015) also included studies evaluating school‐based sexual abuse prevention interventions, but within the broader policy context of child sexual abuse prevention. Radford et al. (2017) included systematic reviews, quantitative studies, and qualitative studies, and examined effective policy and interventions delivered by sectors and institutions to prevent and respond to child sexual abuse operating in jurisdictions outside, but comparable to, England and Wales (Radford et al. 2017). Quadara et al. (2015) included a similar range of studies. Notably, Radford et al. (2017) highlighted that universal or primary prevention responses to child sexual abuse have focused predominantly on teaching children to protect themselves, that limited evidence exists to support the effectiveness of interventions aimed at those with a sexual interest in children (which was corroborated by our search findings), and that evidence for social marketing or the use of media to promote public awareness, recalibrate social norms, and/or promote behaviour change was limited (Radford et al., 2017). Both the Quadara et al. (2015) and Radford et al. (2017) reviews also highlighted the current lack of robust evidence supporting the effectiveness of preventive interventions implemented within organisations (such as using situational crime prevention or safeguarding practices/policies). Radford et al. (2017) noted the particular need to expand safeguarding practices to faith‐based organisations and churches.

Three systematic reviews (McKibbin, 2017; Sherr et al., 2017; South et al., 2015) included prevention interventions in out‐of‐home care settings. Reviews by South et al. (2015) and McKibbin (2017) were both systematic scoping reviews and both had a focus on sexual abuse prevention. The scoping review by South et al. (2015) included seven evaluations of training, support and/or treatment for sexually abusive and/or sexually “acting‐out” children in out‐of‐home care and their caregivers. Of the total included studies, three were effectiveness studies, only one of which included a comparison group. This systematic review reported that the most common programme aim was to promote caregivers' understanding of sexual abuse and its consequences, including the effect of sexual abuse on children's behaviour and needs. Another common aim was to provide caregivers with strategies for coping with, and responding to, children's sexually abusive and/or sexual “acting‐out” behaviours (South et al., 2015). Programmes provided training, treatment or support for the children themselves, involving training/treatment sessions utilising one‐to‐one behavioural management, socialisation, crisis intervention and supportive counselling by psychiatric aids. McKibbin (2017) identified 20 studies, including one systematic scoping review and two RCTs. The authors highlighted that the current evidence base supporting prevention responses to harmful sexual behaviour and sexual exploitation of children and young people living in residential care, is under‐developed (McKibbin, 2017). The review by Sherr et al. (2017) focused on interventions to reduce violence in institutionalised care and included two studies describing caregiver training interventions that consisted of workshops and an instructional system which included training for caregivers.

#### 5.5.2 Disclosure

Disclosure interventions were defined as any intervention that aimed to facilitate, support, or promote the disclosure of child maltreatment. This encompassed a range of universal interventions, such as traditional or social media campaigns, or child helplines, as well as therapeutic interventions for children that aimed to promote disclosure (e.g., play therapy). It included tertiary interventions relating to perpetrators, such as mandatory reporting, and also included any intervention that aimed to promote disclosure within an organisational context (e.g., staff training, organisational guidelines; see Table 1).

##### Primary studies

We did not identify any primary studies that assessed interventions solely aimed at facilitating disclosure. However, multiple prevention interventions included components that aimed to provide children with knowledge and/or skills to disclose maltreatment to a trusted adult. Nine studies evaluating these interventions included participant rates of disclosure either during or directly after participation, and two studies specifically assessed disclosures rates of children currently suspected of experiencing abuse, who had at some point in the past taken part in one of these programs (see Section 5.6).

##### Systematic reviews

One low‐quality largescale rapid review included both primary studies and systematic reviews relating to child sexual abuse, and included 21 studies reporting on interventions implemented at the agency, organisation or community level that may support the disclosure, identification and reporting of child sexual abuse (Radford et al., 2017). The studies reported on a range of interventions, including: proactive outreach and engagement with minority communities; training those who work with children to be alert to the signs of sexual abuse and exploitation; colocated multidisciplinary investigation and response models; protocols and best practice approaches for investigative interviewing; and improved assessment methods and training for professionals. Radford et al. (2017) noted that research on improving disclosure had been largely focused on children and young people who are victims, and that research on improving the disclosure of those who abuse is a relatively recent development. A second systematic review (Quadara et al., 2015), also broad in scope, included a narrative synthesis of both primary and systematic review studies. The review refers to both mandatory reporting and “Working With Children Checks,” however the authors note that there have been few tests of the effectiveness of these schemes.

#### 5.5.3 Response

Response interventions were defined as any intervention that aimed to improve institutional responses to the occurrence of child maltreatment in relation to each of the target populations. Response interventions included legal or regulatory mechanisms aimed at introducing new procedures for institutions to follow, organisational guidelines and/or practices (e.g., response framework), support for the victim and/or family, working with child protection agencies, and providing training and/or crisis support to staff within organisations (see Table 1).

##### Primary studies

Two primary studies evaluated the effectiveness of response interventions.

Cerezo and Pons‐Salvador (2004) used a quasi‐experimental approach to assess a largescale intervention that aimed to increase detection of child maltreatment across a single territory in Spain. The intervention involved professional training based on motivational interviewing approaches and support. It was delivered in multiple settings to professionals from all frontline health and social services agencies, and schools, in the territory.

An RCT reported by Rheingold et al. (2014) compared a web‐based and in‐person training versions of an intervention with a dual focus on preventing and responding to child sexual abuse among children (ranging from 0 to 18 years). It was delivered to staff from youth service organisations including schools, churches, daycare, extracurricular activity agencies, state agencies, group home/residential settings and healthcare settings. The programme included education about child sexual abuse, ways to minimise child sexual abuse, how to recognise the signs and how to respond appropriately when a child discloses (Rheingold et al., 2014).

##### Systematic reviews

We found five low‐quality systematic reviews that included studies examining institutional response interventions (Hermenau et al., 2017; Quadara et al., 2015; Radford et al., 2017; Sherr et al., 2017; South et al., 2015).

Only one of these reviews focussed solely on interventions relating to institutional responses to child maltreatment (Hermenau et al., 2017). This review investigated the effects of structural interventions and caregiver trainings on child development, for children living full time in institutional care environments across the world (e.g., orphanages, residential care). It included interventions that aimed to change the organisational structure and culture of the institutions, as well as the ways in which caregivers interact with children. The review included 24 studies; 15 with experimental and control groups, three of which were RCTs (however, those RCTs did not meet the criteria for inclusion in this EGM, e.g., the maltreatment did not occur in an institution). Fifteen of its studies focused on interventions involving staff training and capacity building, nine studies assessed structural changes implemented within the institution, and one study assessed both (Hermenau et al., 2017). The authors concluded that caregiver trainings, structural changes and enriched caregiving environments in institutional care environments can have beneficial effects on children's emotional, social and cognitive development.

The four remaining reviews included studies assessing response interventions alongside other intervention types (Quadara et al., 2015; Radford et al., 2017; Sherr et al., 2017; South et al., 2015). The review by Sherr et al. (2017) identified three studies that reported on interventions aiming to reduce violence within institutionalised care. Two had a primary focus on staff training, and one compared institutional care with foster care. A scoping review by South et al. (2015) identified 16 studies in order to identify practice elements that aim to prevent child sexual abuse in out‐of‐home care. Seven studies evaluated training, support and/or treatment for sexually abusive and/or “acting‐out” children in out‐of‐home care, and nine retrospective case studies and surveys attempted to identify practices that contributed to, or prevented, child sexual abuse in out‐of‐home care. Two broad reviews (Quadara et al., 2015; Radford et al., 2017) focused on child sexual abuse, and included studies on interventions aimed at improving institutional responses to child sexual abuse. Radford et al. (2017) highlighted the lack of evidence assessing the effectiveness of response interventions within institutions, including religious organisations and institutional care (Radford et al., 2017). While Quadara et al. (2015) included some discussion on response interventions, including institutional policies for identifying and reporting maltreatment, no studies are included that assess the effectiveness of response interventions.

#### 5.5.4 Treatment

Treatment interventions were defined as any intervention that aimed to provide a therapeutic response to a target population. This included therapeutic interventions provided to children who experienced child maltreatment in institutions, and interventions targeted at perpetrators of institutional child abuse (see Table 1)The BEIP publications are included here, because foster care was provided as treatment for young children who spent their early lives in institutionalised care.

##### Primary studies

Two primary studies assessed the effectiveness of treatment interventions, including the six reports describing the BEIP. These are summarised in Appendix 6.

The BEIP study randomly assigned children in Romanian orphanages to remain in institutional care or be removed and placed in high‐quality foster care (the treatment intervention). Each of the six reports was assessed as raising some concerns relating to risk of bias. A range of outcomes was reported for children aged between 6 and 32 months, with follow‐up assessments reported across the ages of 42 months (Smyke et al., 2010) and 54 months (Johnson et al., 2010), and again between age 8 and 16 years (Bick et al., 2015; Humphreys et al., 2015; Troller‐Renfree et al., 2015; Wade et al., 2018).

Sullivan et al. (1992) used a quasi‐experimental approach to assess the effectiveness of a treatment intervention for hearing‐impaired children between the ages of 12 and 16 years, who had been sexually abused while attending a residential school for the deaf. The psychotherapeutic intervention was offered to the children by the school and involved 2 h of individual therapy per week for 36 weeks, delivered by a clinical psychologist and a supervising psychiatrist with expertise in the psychology of deafness and fluency in sign language (Sullivan et al., 1992).

##### Systematic reviews

The scoping review by McKibbin (2017) examined treatment interventions focused on harmful sexual behaviour and child sexual exploitation among children and young people living in out‐of‐home care. This review included 17 papers describing interventions, including treatment interventions, for young people who display harmful sexual behaviour. The authors concluded that evidence about the elements of a successful tertiary prevention response, including trauma‐informed therapeutic treatment was well‐developed particularly in the UK. The review by Quadara et al. (2015) examined prevention, early intervention and therapeutic responses to child sexual abuse and described one study comparing children in institutions with home‐based care as a form of treatment.

#### 5.5.5 Alignment with the WHO‐INSPIRE categories

The interventions described in the included in primary studies aligned with three of the seven WHO‐INSPIRE strategies, which were:

- Education and life skills: This strategy aims to increase children's access to more effective, gender‐equitable education, social‐emotional learning and life‐skills training, and ensure that school environments are safe. Interventions relevant to this category can include establishing a safe and enabling school environment, improving children's knowledge about sexual abuse and how to protect themselves against it, adolescent intimate partner violence prevention programmes, and life and social skills training programme. Fifty‐eight primary studies of interventions focused on education and life skills either as a primary focus (*n* = 55) or in combination with other strategies (*n* = 3).
- Norms and values: This strategy aims to strengthen norms and values that support nonviolent, respectful, nurturing, positive and gender equitable relationships for all children and adolescents. Interventions relevant to this category include community mobilisation programmes, bystander interventions and small group programmes that challenge harmful gender and social norms. Four studies evaluated interventions relating to norms and values. This was the primary approach of one intervention (*n* = 1), and was used in combination with other strategies for the remaining interventions (*n* = 3).
- Response and support services: This strategy aims to improve access to good quality health, social welfare and criminal justice support services for all children who need them—including for reporting violence—to reduce the long‐term impact of violence. Interventions in this category can include counselling and therapeutic approaches, screening combined with interventions, treatment programmes for juvenile offenders in the criminal justice system, and foster care interventions involving social welfare services. Two studies assessed interventions focused on response and support services (*n* = 2).

### 5.6 Outcomes

**Figure 11:**
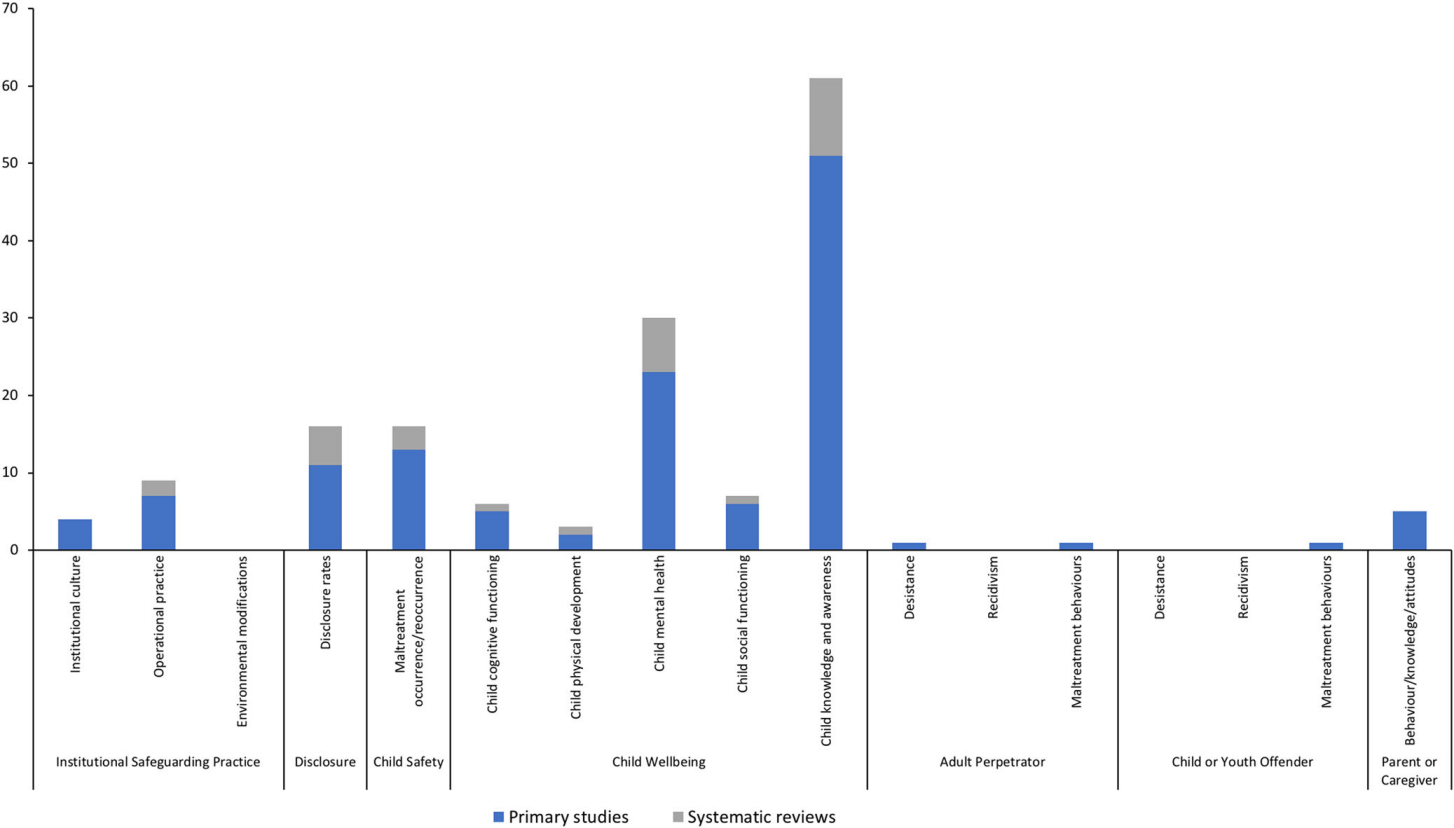
Number of included studies that reported each of the outcome categories and subcategories (*N* = 84)

This section describes the outcomes of interest to the EGM that were measured and reported across the included studies. This section reports each of the publications of the studies separately. Figure 11 details the number of studies that included each of the EGM outcome categories.

#### 5.6.1 Outcomes related to institutional safeguarding practice

##### Primary studies

We found 12 studies reporting outcomes related to institutional safeguarding practice: eight focused on operational practice (Baker‐Henningham et al., 2016; Cerezo & Pons‐Salvador, 2004; del Campo Sánchez & Sánchez, 2006; Gushwa et al., 2018; Kolko et al., 1987, 1989; MacIntyre & Carr, 1999a; Rheingold et al., 2014) and four on institutional culture (McElearney et al., 2018; Merrill et al., 2018; Nkuba et al., 2018; Ssenyonga et al., 2018).

Operational practice included both prevention and response interventions targeting staff in schools (Baker‐Henningham et al., 2016; del Campo Sánchez & Sánchez, 2006; Gushwa et al., 2018; Kolko et al., 1987, 1989; MacIntyre & Carr, 1999a) and youth service agencies and organisations (Cerezo & Pons‐Salvador, 2004; Rheingold et al., 2014). An RCT undertaken by Gushwa et al. (2018), with a high risk of bias, assessed a 1‐h online training programme focused on debunking misconceptions and fears/biases associated with responding to, and reporting, suspected abuse. This study used a 13‐item instrument to measure K‐12 teachers' knowledge awareness, including prevalence rates, types of CSA behaviours, impact of CSA on children, signs and symptoms, reporting responsibilities, and responses to suspected abuse (Gushwa et al., 2018). A second RCT, with some concerns relating to risk of bias, offered a pre‐training session to both teachers and parents that aimed to provide them with knowledge about sexual abuse and prepare them for interacting with students undergoing a school‐based sexual abuse prevention intervention (del Campo Sánchez & Sánchez, 2006). They reported an increase in teacher‐reported conversations with students relating to sexual abuse (del Campo Sánchez & Sánchez, 2006). Rheingold et al. (2014) reported on a RCT, assessed as having some concerns for risk of bias, that included a self‐report measure of child sexual abuse prevention behaviours by staff in youth services organisations (i.e., teachers, childcare personnel, clergy) after receiving web‐based or in‐person training on preventing and responding to child sexual assault. An RCT from Baker‐Henningham et al. (2016) will assess an intervention (The Irie Classroom Toolbox), which involves training teachers in classroom behaviour management and in strategies to promote children's social‐emotional competence, with the aim of decreasing violence in Jamaican preschools. The Kolko et al. (1987, 1989) and MacIntyre and Carr (1999a) studies each used quasi‐experimental designs to assess the effectiveness of a school‐based prevention programme (Red Light Green Light; Stay Safe), each used a teacher questionnaire to assess teacher knowledge and attitudes about child sexual abuse. Cerezo and Pons‐Salvador (2004) used a quasi‐experimental design to assess whether professional training and support offered to frontline health and social services agencies and school professionals increased the detection of cases of child maltreatment (i.e., number of children with signs of maltreatment, as detected by child protection services) across a single territory in Spain.

Four primary studies reported outcomes related to institutional culture: two ongoing studies (McElearney et al., 2018; Ssenyonga et al., 2018), and two completed RCTs (Merrill et al., 2018; Nkuba et al., 2018), both assessed as having a high risk of bias. McElearney et al. (2018) will use a composite teacher‐report survey to measure the following outcomes: teacher willingness to teach sexual health and safety; perceived confidence in their own skills to manage sensitive issues; attitudes toward teaching and learning about sensitive issues and sexual health education safe messages; and teacher perceptions of their school culture indicating how frequently various practices occur. This whole school prevention intervention aims to teach children how to keep safe from all forms of maltreatment carried out online or using digital technology, abuse perpetrated by other children, and bullying. It involves training and support for teachers and whole school staff and parent directed homework activities (McElearney et al., 2018). Using a randomised control trial, Ssenyonga et al. (2018) will assess a preventative intervention (Interaction Competencies with Children for Teachers), which targets teachers and aims to foster better adult‐child interactions while reducing the occurrence of violent discipline. They will assess change to teachers' positive attitudes toward violent disciplining and teachers' use of violent disciplinary methods using the Conflict Tactics Scale.

In an assessment of the Good School Kit, Merrill et al. (2018) used both single and composite measures to assess operational culture. School operational culture was assessed by investigating relational, psychological and structural domains. The relational domain examined: students' feelings of emotional support from teachers and peers; staffs' perceived relationship with students, colleagues and caregivers; and caregivers' perceived relationship with staff. The psychological domain assessed: degree of identification with the school among students and staff; acceptance of physical discipline practices in school among students and staff; and acceptance of sexual violence from teachers among students. The structural domain examined: students' perceived level of involvement with school operations; staffs' perceived level of involvement in school operations among staff and students; and caregivers' perceived level of involvement in school operations. Nkuba et al. (2018) used teacher and student reported outcome measures (using questionnaires) to assess attitudes to physical and emotional violence toward children, to evaluate the effectiveness of training delivered to teachers in Tanzanian secondary schools aimed at preventing violent discipline and improving teacher–student relationships.

##### Systematic reviews

Two recently published systematic reviews included interventions delivered in out‐of‐home care settings (Hermenau et al., 2017; McKibbin, 2017). Hermenau et al. (2017) included studies that assessed interventions aimed at improving the quality of care in institutional environments, reporting a broad range of outcome measures and measurement instruments used to assess changes in caregiving and institutional quality and attachment. They included institutional safeguarding practice outcomes relating to both operational practice (e.g., measures assessing changes in caregiving quality, child‐caregiver ratios) and the institutional environment (e.g., measures of environmental quality, structural changes to the institutional environment). A scoping review by McKibbin (2017) included studies reporting on interventions addressing harmful sexual behaviour and child sexual exploitation for children and young people living in residential care. The reported institutional safeguarding practice outcomes were about operational practice, and included outcomes measuring staff members' knowledge about, and skills relating to, recognising childrens' problematic sexual behaviour (McKibbin, 2017).

#### 5.6.2 Outcomes related to child maltreatment disclosure

##### Primary studies

Six RCTs, four with some concerns relating to risk of bias (Barron & Topping, 2013; del Campo Sánchez & Sánchez, 2006; Hazzard et al., 1991; Oldfield et al., 1996), and two with a high risk of bias (Daigneault et al., 2015; Devries et al., 2015), and five QEDs (Czerwinski et al., 2018; Elfreich et al., 2020; Kolko et al., 1987, 1989; MacIntyre & Carr, 1999b) reported outcomes relating to child maltreatment disclosure. All of these studies evaluated school‐based interventions aiming to prevent child maltreatment. Outcome measures included: participant, teacher and/or parent reported disclosure of sexual abuse over the course of the intervention and evaluation (Barron & Topping, 2013; del Campo Sánchez & Sánchez, 2006; Hazzard et al., 1991; Kolko et al., 1987, 1989; Oldfield et al., 1996); child reported courses of action in response to hypothetical scenarios, including possible disclosure options (Czerwinski et al., 2018); child‐reported likelihood of future disclosure (Kolko et al., 1989); youth recognition of sexual assault and response to a hypothetical disclosure of sexual assault (Daigneault et al., 2015); and students' self‐reports of physical violence from school staff (assessed in a follow‐up survey) (Devries et al., 2015). Two studies (Elfreich et al., 2020; MacIntyre & Carr, 1999b) specifically assessed disclosure rates of children who were suspected of experiencing maltreatment and who had at some point in the past taken part in a school‐based prevention programme. MacIntyre and Carr (1999b) reported children's disclosure of sexual abuse after they had been referred to a sexual abuse assessment unit, and Elfreich et al. (2020) assessed child disclosure of abuse during forensic interviews.

##### Systematic reviews

We found three systematic reviews examining interventions' impact on disclosure‐related outcomes. A high‐quality review by Walsh et al. (2015) (an update of Zwi et al., 2007) included school‐based sexual abuse programmes, and reported on disclosure of sexual abuse by child or adolescent participants during or after undertaking a programme. Pitts (2015) included studies that reported on the disclosure of child sexual abuse. Radford et al. (2017) also included studies that reported on measures of safe disclosure (e.g., rates of disclosure) to peers, adults, institutions and services, including disclosure of nonrecent abuse.

#### 5.6.3 Outcomes related to child safety—Maltreatment occurrence or reoccurrence

##### Primary studies

We found 13 primary studies. Eight completed studies, all with high risk of bias (GST; Nkuba et al., 2018; Taylor et al., 2010) and three of the protocols in the EGM (Baker‐Henningham et al., 2016; McElearney et al., 2018; Ssenyonga et al., 2018) reported/will report outcomes related to child maltreatment occurrence/reoccurrence. Eleven studies evaluated interventions focused on preventing maltreatment in educational settings (e.g., schools, day‐care), with most addressing physical violence. Outcome measures included: student self‐reported violence perpetrated by staff (Good School Kit); teacher and student reports of emotional and physical violence (Nkuba et al., 2018); and student‐reported exposure to violence (Ssenyonga et al., 2018); and teacher‐reported use of violent disciplinary methods (Ssenyonga et al., 2018). A further two QED studies used a child‐report questionnaire to determine childrens' experiences of inappropriate touching involving an uncomfortable or potentially abusive interaction (Kolko et al., 1987, 1989).

##### Systematic reviews

We found three recent low‐quality systematic reviews including interventions delivered in residential care settings that reported on child maltreatment occurrence/reoccurrence (Hermenau et al., 2017; Sherr et al., 2017; South et al., 2015). Outcomes examined included: self‐reports or observations of maltreatment from staff/adults (physical/emotional), as well as peer to peer violence in institutional care (Sherr et al., 2017); sexual abuse in out‐of‐home care (South et al., 2015); exposure to violence of children living in a child care institution (Hermenau et al., 2017); and documented abuse in official records (Sherr et al., 2017).

#### 5.6.4 Outcomes related to child wellbeing

Child wellbeing outcomes were coded into five subcategories: knowledge and awareness, mental health, cognitive functioning, social functioning, and health and development.

##### Primary studies

Across the child wellbeing outcome subcategories, more primary studies reported outcomes relating to knowledge and awareness (*n* = 51) than the number of primary studies reporting mental health outcomes (*n* = 23), outcomes relating to child cognitive functioning (*n* = 5), social functioning (*n* = 6) or physical health and development (*n* = 2) (see Figure 2).

All 51 studies reporting child knowledge and awareness outcomes evaluated curriculum‐based prevention interventions delivered in educational settings, with most focussing on child sexual abuse. Thirty‐three were completed RCTs, approximately a third of which were at a high risk of bias, with the remainder assessed as raising some concerns about risk of bias.

The most highly represented outcomes across these studies were:

- Knowledge about child sexual maltreatment, prevention and protective strategies. Multiple outcome measures were used to assess varying components of knowledge (e.g., knowledge about child sexual maltreatment, ability to differentiate between different types of touches, ability to identify appropriate and inappropriate scenarios, knowledge about how to act when confronted with inappropriate scenarios, knowledge about how and who to disclose to).
- Protective skills. Multiple outcome measures were used to assess whether children acquired protective skills as a result of the intervention. These were commonly assessed using hypothetical scenarios, where participants responded to a written (e.g., what‐if‐situations‐test: Nemerofsky et al., 1986; Wurtele et al., 1998) or other (e.g., roleplay) scenario.

Fewer studies assessed changes in participant knowledge and awareness about other child maltreatment types, such as physical or emotional abuse (*n* = 7) (Barron & Topping, 2013; Dake et al., 2003; Dhooper & Schneider, 1995; Dryden et al., 2014; Edwards et al., 2019; Kraizer, 1991; Wolfe et al., 1986).

Twenty‐three studies reported outcomes relating to child mental health. Mental health outcomes were measured in three studies evaluating treatment interventions (two RCTs at high risk of bias; 1 QED) (Humphreys et al., 2015; Sullivan et al. 1992; Troller‐Renfree et al., 2015), and 20 studies evaluating preventive interventions (including, two GST publications). Of the 20 prevention interventions, three were on‐going RCTs (Baker‐Henningham et al., 2016; McElearney et al., 2018; Ssenyonga et al., 2018), 12 were completed RCTs (four with a high risk of bias: Devries et al., 2015; Knight et al., 2018; Ratto & Bogat, 1990; Van Lieshout et al., 2019), and five were quasi‐experimental studies. The studies that evaluated prevention interventions reported outcomes relating to internalising and externalising behaviours, including anxiety, subjective wellbeing, self‐esteem and emotional intelligence. These studies focused primarily on sexual and physical maltreatment, and all but one was delivered in educational settings (Van Lieshout et al., 2019; delivered in a group home for adolescent boys). The three studies evaluating treatment interventions reported outcomes relating to internalising and externalising behaviours among abused children attending a residential school for the deaf who received psychotherapy (Sullivan et al., 1992), and prosocial behaviour, internalising and externalising behaviours in two studies describing outcomes of the BEIP (Humphreys et al., 2015; Troller‐Renfree et al., 2015). A range of questionnaires and instruments were used to assess these outcomes. These included unvalidated measures, as well as commonly used, and well validated instruments including the Strengths and Difficulties Questionnaire, Child Behaviour Checklist, State Trait Anxiety Inventory, and the Diagnostic Interview Schedule for Children IV.

Five primary studies reported outcomes related to cognitive functioning, including two RCTs assessing prevention interventions and three publications from the BEIP RCT. Education‐related outcomes were also coded under this subcategory. The Devries et al. (2015) RCT, assessed as having a high risk of bias, evaluated the GST intervention in Ugandan primary schools, and reported scores of educational performance relating to literacy and numeracy. This was the sole primary study to report on educational outcomes. Three publications reported cognitive functioning outcomes of participants in the BEIP study, and all three had some concerns relating to risk of bias. These publications reported on mental development and intelligence scores over time, and also reported measures of memory and executive functioning which can be an indicator of children's ability to regulate behaviour and emotion (Johnson et al., 2010; Smyke et al., 2010; Wade et al., 2018). The ongoing Baker‐Henningham et al. (2016) RCT plans to assess outcomes for school attendance obtained from school records.

Four RCTs (Daigneault et al., 2012; del Campo Sánchez & Sánchez, 2006; Smyke et al., 2010; Van Lieshout et al., 2019; all some concerns relating to risk of bias) and 2 QEDs (Hebert et al., 2001; Taal & Edelaar, 1997) reported social functioning outcomes. Two RCTs assessed social competencies and skills, including participants' confidence in others, respect toward one another, empathy and social norms (Daigneault et al., 2012; Van Lieshout et al., 2019). Van Lieshout et al. (2019) evaluated an education programme to promote respectful (sexual) relationships and to prevent sexual harassment delivered to boys aged 12–18 residing in residential care. This study assessed changes in communication, self‐control, boundaries, dating violence, adverse sexual beliefs and rape attitude. The two remaining RCTs evaluated differences in attachment and caregiver relationships (del Campo Sánchez & Sánchez, 2006; Smyke et al., 2010). Taal and Edelaar (1997) reported on social connections and relationships, using a child‐report questionnaire to assess changes in childrens' relationships with classmates and teachers. Adaptive behaviours were measured by Hébert (2001), including positive and negative behavioural responses to participation in a sexual abuse prevention programme.

Two BEIP references, with some concerns for risk of bias, reported on physical health and development, both in relation to brain development (Bick et al., 2015; Johnson et al., 2010). The specific outcomes reported included measures of brain white matter integrity (Bick et al., 2015) and measures of auxology (i.e., human physical growth incorporating length and height, occipital frontal circumference, weight) (Johnson et al., 2010).

##### Systematic reviews

Nine systematic reviews included studies that assessed an interventions' impact on child knowledge and awareness. Walsh et al. (2015) evaluated whether school‐based sexual abuse programmes increased knowledge of sexual abuse or sexual abuse prevention concepts, protective behaviours, retention of protective behaviours over time, and retention of knowledge over time. Six low‐quality reviews also examined sexual abuse prevention interventions in educational settings and also reported outcomes relating to knowledge of child sexual abuse, as well as protective behaviours (Heidotting, 1996; Pitts, 2015; Quadara et al., 2015; Radford et al., 2017; Topping & Barron, 2009). The review by Sherr et al. (2017) reported outcomes relating to risk awareness and behaviour of children in institutional care. One low‐quality systematic review examined sexual abuse prevention interventions delivered in residential care, and reported outcomes on child knowledge of normal sexual development and safe sexual relationships (McKibbin, 2017). A low‐quality review by Ricardo et al. (2011) included studies assessing interventions aimed at preventing boys' and youths' use of sexual violence in community and school settings, and reported outcomes relating to attitudes toward violence, acceptance of rape myths and bystander attitudes (Ricardo et al., 2011).

Six systematic reviews included studies that assessed an interventions' impact on child mental health outcomes. One high‐quality systematic review (Walsh et al., 2015) evaluated whether participation in school‐based sexual abuse programmes increased child anxiety or fear. Likewise, two low‐quality reviews evaluated whether children displayed increased levels of fear or anxiety (Pitts, 2015; Topping & Barron, 2009), self‐esteem or aggression (Topping & Barron, 2009) after participation in sexual abuse prevention interventions in educational settings. A further low‐quality review by Sherr et al. (2017) included evaluations of interventions aiming to decrease abuse experienced by children in institutionalised care. These studies measured child depression, externalising and internalising symptoms and suicidality using a range of measures, including the Strengths and Difficulties Questionnaire, Children's Depression Inventory, and the Mini‐International Neuropsychiatric Interview for Children and Adolescents. A further low‐quality review that included studies evaluating interventions aimed at addressing physical and emotional abuse and neglect within institutional care, reported child outcomes relating to depression, internalising and externalising symptoms, anxiety and posttraumatic stress symptom (Hermenau et al., 2017).

One low‐quality systematic review that assessed interventions delivered in institutionalised care, included studies that reported child cognitive functioning. Cognitive functioning outcomes included child mental development, language development and intelligence (Hermenau et al., 2017). These were measured using a range of instruments, including the Ankara Development Schedule, Bayley Scales of Infant Development II, Catell Infant Intelligence, and the Griffiths Mental Development Scale.

Hermenau et al. (2017) was the only systematic review that included studies evaluating interventions' impact on child social functioning, including outcomes relating to children's social‐emotional competencies and skills, as well as attachment and caregiver relationships.

Hermenau et al. (2017) was also the only systematic review that reported on child physical development and health outcomes, including psychomotor development specifically, as well as general development (including cognitive, language, social‐emotional development).

#### 5.6.5 Outcomes related to adult perpetrators or child/youth offenders

##### Primary studies

We found two studies reporting outcomes relating to adult perpetrators and child/youth offenders. Baker‐Henningham et al. (2016) reported on an ongoing RCT for a prevention focused study that plans to include observations of teachers' use of violence against children in daycare settings in Jamaica. Edwards et al. (2019) evaluated a bystander‐focused interpersonal violence prevention programme with high school students in the United States. The study was an RCT with high risk of bias, and self‐reported youth offender outcomes including sexual harassment, sexual assault and stalking victimisation and perpetration among high school students (Edwards et al., 2019).

##### Systematic review

No systematic review reported outcomes for adult perpetrators or child/youth offenders that specifically related to child maltreatment that occurred in an institutional setting.

#### 5.6.6 Outcomes related to parent or caregiver behaviour, knowledge or attitudes

##### Primary studies

We identified five studies reporting parent or caregiver behaviour, knowledge or attitudes; two RCTs (high risk of bias: Merrill et al., 2018; Wurtele, Gillispie, et al., 1992), one ongoing RCT (McElearney et al., 2018), and one QED study (Kolko et al., 1987). Wurtele, Gillispie, et al. (1992) compared teachers and parents as instructors of a personal safety programme delivered to preschool children and assessed parents' perceptions of their child's understanding of protective behaviour concepts, and their application of those behaviours. Merrill et al. (2018) assessed changes in parental normative beliefs relating to school based physical discipline when assessing the GST programme (Merrill et al., 2018). In their evaluation of a multicomponent “whole‐school” programme designed to teach 4–11 year olds how to keep safe from all forms of maltreatment, McElearney et al. (2018) will assess parents' confidence in talking to their children about keeping safe. Kolko et al. (1987) reported changes to parental knowledge about sexual abuse when evaluating a school‐based sexual abuse prevention intervention.

##### Systematic review

No systematic review reported outcomes related to parent or caregiver behaviour, knowledge or attitudes.

### 5.7 Other outcomes

#### 5.7.1 Implementation outcomes

Of the primary studies, 23 reported outcomes relating to the implementation of the intervention, including one ongoing study (Ssenyonga et al., 2018). Outcomes representing feasibility (i.e., the utility, fit or practicality of the implemented programme), adoption (i.e., uptake or utilisation of the intervention), fidelity (i.e., the degree to which an intervention was implemented as it was intended), acceptability (i.e., perception among implementation stakeholders that an intervention is satisfactory in relation to content, complexity, comfort, delivery and credibility) and penetration (i.e., reach, spread and institutionalisation) (Proctor et al., 2011), were reported across these studies. Aspects of fidelity were assessed in 15 studies, acceptability was reported in 11, five studies reported aspects of penetration (Devries et al., 2017; Knight et al., 2018; Nkuba et al., 2018; Ssenyonga et al., 2018; White et al., 2018), feasibility (Nkuba et al., 2018; Ssenyonga et al., 2018) and adoption (Devries et al., 2017; Knight et al., 2018) were each reported by two studies.

Almost all of the studies reporting on fidelity used either checklists or questionnaires to assess how closely the implemented programme adhered to the intended intervention, and almost all were evaluations of an intervention delivered to children, teachers or parents in educational settings (excepting Rheingold et al., 2014, delivered across youth service organisations). These assessments varied in their comprehensiveness, but generally included how much core content was covered and/or which activities had been completed in the session/s or workshop/s. For some, additional information was captured, such as the timeframe or mode of delivery, or whether any other modifications were made to the intervention's delivery. Of the 13 studies reporting on intervention fidelity; all or a proportion of the fidelity checklists/questionnaires were completed by independent assessors (e.g., research assistant/s, volunteer/s) in eight studies (Baker et al., 2012; Daigneault et al., 2012, 2015; Hebert et al., 2001; Jin et al., 2017; Kolko et al., 1989; Pulido et al., 2015; Rheingold et al., 2014), and all or a proportion of the fidelity checklists/questionnaires were completed by intervention facilitators (e.g., teachers, counsellors) in seven of the studies (Barron & Topping, 2013; Daigneault et al., 2012; Kenny et al., 2012; Pulido et al., 2015; Warden et al., 1997; White et al., 2018; Zhang et al., 2013).

Most studies assessing the acceptability of an intervention used a questionnaire, and were evaluations of an intervention delivered to children, teachers or parents in educational settings. Questionnaires were completed in writing or face‐to‐face interviews, and generally assessed satisfaction and/or requested feedback on content of an intervention. These were completed by children (Barron & Topping, 2013; del Campo Sánchez & Sánchez, 2006; Grendel, 1991; Hebert et al., 2001; Jin et al., 2017; Kraizer, 1991; MacIntyre & Carr, 1999a; Wurtele, Gillispie, et al., 1992), teachers/programme facilitators (Barron & Topping, 2013; Jin et al., 2017; Kraizer, 1991; MacIntyre & Carr, 1999a; Nkuba et al., 2018; Ssenyonga et al., 2018; Wurtele, Gillispie, et al., 1992) and/or parents (Grendel, 1991; Kolko et al., 1987; MacIntyre & Carr, 1999a; White et al., 2018; Wurtele, Gillispie, et al., 1992).

Five studies reported several components of implementation. An RCT by Nkuba et al. (2018), evaluating the Interaction Competencies with Children for Teachers (ICC‐T) programme to prevent violent discipline in schools in Tanzania, used multiple measures to report an overall assessment of feasibility. Feasibility was assessed using teacher responses to survey items, and related to the demand for the programme (i.e., attitudes toward the use of violence to discipline students), the applicability of the programme to teachers (e.g., relevance of the workshop content to the daily work), and acceptability (e.g., satisfaction, the topics of the workshop related to the daily work). Aspects of intervention penetration were also reported, including self‐reports of teachers' integration of the core intervention strategies at follow‐up (Nkuba et al., 2018). The ongoing study by Ssenyonga et al. (2018), evaluating the same intervention in Uganda, will use similar methods to assess implementation as those reported by Nkuba et al. (2018).

A process evaluation of the Good School Kit included measures relating to the adoption of the programme, fidelity and penetration (reported in Devries et al., 2017; Knight et al., 2018). Adoption of the school kit elements by schools was assessed independently by a set of questions, answered once a term by a teacher representative, designed to determine the presence of the intervention structures and elements implemented at the school (Knight et al., 2018). Other process measures captured aspects of the intervention's fidelity and penetration, including: routine data collection relating to programme delivery in schools; school‐led monitoring of the activities planned and completed across a school term; and completed surveys to determine the exposure of both teachers and students to components of the intervention.

Outcomes related to adoption, fidelity and acceptability were reported by White et al. (2018), in a study conducted in Australia evaluating a child sexual assault prevention programme delivered in primary schools. Implementation measures included a record of child attendance at each session, a facilitator checklist recording whether core content and activities were undertaken, and a parent/caregiver questionnaire assessing satisfaction with their child's involvement in programme (White et al., 2018).

#### 5.7.2 Adverse outcomes

It is not the aim of an EGM to report on the direction of findings in relation to the reported outcomes. Therefore, we cannot report adverse effects on outcomes where the intervention had a negative effect, but was hoped to have a positive effect (e.g., knowledge of sexual abuse). However, some studies included specific outcomes that aimed to capture adverse effects. These outcomes included: measures of anxiety, fear and touch aversion, which were commonly used to assess whether education‐based prevention programmes targeting the sexual abuse of children had a negative effect on their well‐being. For the most part, these programmes did not appear to adversely impact children. A single study (Taylor et al., 2010) reported that an intervention addressing gender violence and sexual harassment, delivered to sixth and seventh graders, reduced peer violence victimisation and perpetration, but may have increased dating violence perpetration, or at least the reporting of it.

### 5.8 Subgroup analyses

#### 5.8.1 Gender

Of the completed primary studies, 26 reported results disaggregated by sex (i.e., they reported differences between males and females), and 43 studies either did not conduct, or did not report, a gender‐specific approach to their analysis of the intervention's effectiveness. One of these primary studies included male participants only (Van Lieshout et al., 2019). Two protocols (Baker‐Henningham et al., 2016; McElearney et al., 2018) reported that a gender analysis will be undertaken; the other protocol did not include a planned gender analysis (Ssenyonga et al., 2018).

Of the 26 studies, 22 assessed curriculum‐based preventive interventions delivered in educational settings, and 10 of these reported differences between males and females for at least one outcome (Bustamante et al., 2019; Czerwinski et al., 2018; Jin et al., 2017; Elfreich et al., 2020; Hazzard et al., 1991; Hillenbrand‐Gunn et al., 2010; MacIntyre & Carr, 1999b; Oldfield et al., 1996; Snyder, 1986; Weatherley et al., 2012). Three GST related publications assessing the implementation and/or effectiveness of a whole school prevention programme addressing physical violence in schools perpetrated by staff, reported that the intervention produced more positive results for male students than for female students (Devries et al., 2015; Devries et al., 2017; Knight et al., 2018). Across three reports of the BEIP study, two reported differences between boys and girls in relation to child mental health outcomes for internalising and externalising symptoms and caregiver attachment (Humphreys et al., 2015; Smyke et al., 2010), and one additional report showed no effect of gender (Johnson et al., 2010). Sullivan et al. (1992) examined differences in behavioural symptoms (including internalising symptoms, somatic, schizoid, uncommunicative and obsessive behaviours) between sexually abused boys and girls attending a residential school for the deaf, after receiving a psychotherapeutic treatment (Sullivan et al., 1992). Two studies showed that girls were significantly more likely to disclose maltreatment than boys (Elfreich et al., 2020; MacIntyre & Carr, 1999b).

## 6 DISCUSSION

The objectives of this EGM were twofold: (a) Provide a structured and accessible collection of existing evidence from finalised and ongoing overviews of systematic reviews, systematic reviews and effectiveness studies of interventions addressing child maltreatment—for those who work to fund, develop, implement, and evaluate interventions aimed at ensuring children's safety in institutional settings; (b) Identify gaps in the available evidence on interventions addressing child maltreatment—thereby helping to inform the research agendas of funders and other organisations.

### 6.1 Quality of the evidence

Most of the studies included in the EGM are low to moderate quality. Ten of 12 systematic reviews received a low‐quality rating, and 19 of the 49 included RCTs were assessed as having a high risk of bias (low‐quality). We only found one high‐quality systematic review, and no RCTs which we assessed as having a low risk of bias (high quality). Therefore, any assessment of effectiveness of the interventions on the reported outcomes should be cautiously interpreted.

### 6.2 Gaps and strengths in the evidence

Overall, there were more gaps across the EGM than areas with high quality evidence. This, combined with the fact that most studies were published in the last five years, suggests that empirical research on the effectiveness of interventions addressing child maltreatment in institutions is very much at an early stage and highlights a need for considerable future research.

Most studies focused on children, on prevention, and on sexual abuse specifically. This is not proportionate to the prevalence of different maltreatment types. For example, current estimates suggest that physical abuse is more, or at least equally, prevalent as sexual abuse (WHO, 2014). Most studies targeted universal child populations, with far fewer targeting children who are high‐risk or who had already been exposed to maltreatment. That a majority of the studies evaluate interventions for children raises some concerns that could be framed as “unintended harm.” While children have rights to provision, protection and participation in areas that affect them, relying solely on interventions focussing on children potentially places the burden of responsibility of prevention and disclosure of child maltreatment on children, rather than on perpetrators of abuse or on the organisations that serve them. Of further concern is that, by focusing on children in this way, the child may feel responsible or may be blamed if maltreatment occurs. Sadly, there is a long history of blaming the victim, especially with respect to violent sexual offenses such as rape. Asking the question, “What can I do differently to prevent becoming a victim?” can easily translate into self‐blame if maltreatment occurs. There is a clear need for more high‐quality evaluations of interventions that cover the whole spectrum of players that this issue concerns, including children, perpetrators, adults in institutional environments, as well as the institutional environment itself. In some cases, this kind of research can be unpopular and difficult to promote or fund, such as research on offenders. However, in this instance the onus of responsibility should be on governments, funding agencies, criminal justice systems and the institutions themselves, who have been unwilling or unable to fund offender prevention, response, and treatment interventions now and in the past.

#### 6.2.1 Institutional settings

Education and early childhood settings were by far the most well‐studied. This is perhaps not surprising, given that most children have more contact with schools than any other institution and studying children in schools is relatively easy. In contrast, evidence assessing the effectiveness of interventions across other institutions, such as OOHC (e.g., foster care, residential care, orphanages), was very limited. For many types of institution within our scope, there were no specific studies at all. For instance, there were no studies specifically targeting religious organisations, sports clubs, or other recreational settings.

There are several potential explanations for this. While it is certainly the case that many institutional settings have not adequately addressed child maltreatment, there are also instances where interventions have been implemented, but have not yet been evaluated or have not been evaluated in an institutional context. The EGM's selection criteria excluded studies that did not explicitly define an institutional setting. However, there are also evaluations that either focus on maltreatment in family settings, or do not specify where the maltreatment occurred. Taking this into account, it is possible that existing evidence‐based interventions targeting general populations, or specific populations outside of an institutional setting, may also be effective, or may be adapted and be effectively used within an institutional context. For instance, interventions targeting sexual abuse perpetrators could possibly be adapted to specifically target people who perpetrated sexual abuse in an institutional setting. Or, interventions targeting staff in schools may be adapted to target staff in other organisational contexts. However, this approach has limitations: institutional environments are diverse, and one‐size‐fits‐all interventions are unlikely to be effective without at least some modifications. There are also differences in risk factors for perpetrators and victims, as well as differences in the experience, perpetration and response to maltreatment both across different institutional settings and also when compared to other settings where maltreatment occurs (Quadara et al., 2015; Radford et al., 2017). These factors would need to be considered, and likely a strong implementation plan developed and executed well, when adapting existing interventions to (other) institutional environments.

#### 6.2.2 Geographic coverage

Though the studies look at many countries, the evidence is dominated by studies undertaken in the US and Europe. It is clear therefore, that the available research does not currently represent countries with the largest populations (and, potentially the greatest incidence of child maltreatment), nor does it represent countries with the highest estimated prevalence of child maltreatment (WHO, 2014).

#### 6.2.3 Target population

Most of the included studies assessed education or skills‐based interventions delivered to children. Fewer were delivered to institutional staff, teachers and/or adult care providers. There was a lack of evidence targeting adult perpetrators and only one study of youth offenders, and where evidence was identified, the focus was primarily on children who display sexually aggressive behaviour toward other children. Only a third of studies analysed differences in outcomes between girls and boys. For those that did, several found different outcomes by gender for at least one reported outcome. There are multiple reasons why boys and girls may respond differently to an intervention, and future research should consider gender‐specific interventions or include analyses that allow an evaluation to determine any differential impact an intervention may have on boys and girls.

#### 6.2.4 Type of maltreatment

Most interventions focussed on sexual abuse—and specifically on preventing sexual abuse. Though we did identify a cluster of studies focused on addressing physical violence in schools (including harsh discipline), far fewer studies targeted other maltreatment types.

#### 6.2.5 Intervention type

A major gap was identified in relation to studies evaluating interventions that specifically aimed to improve disclosure. Interventions with a particular focus on disclosure were not studied in any of the primary studies that were found, and included in only one systematic review. However, there were a number of studies reporting on disclosure outcomes relating to prevention programs which inlcuded disclosure components. Evidence supporting the effectiveness of organisational response‐based approaches was lacking in both breadth and quality. Of the small number of studies, only one was an RCT, which evaluated a very brief staff training intervention. Studies that assessed treatment interventions that addressed child maltreatment experienced or perpetrated in institutional settings were also extremely limited and solely focused on out‐of‐home care settings. Prevention‐based interventions were by far the most highly represented group of interventions. Of these, most reported on school‐based interventions that primarily aimed to provide children with knowledge and skills to better protect themselves from maltreatment, often with elements geared toward normalising and promoting helpseeking. This was also reflected in the findings for alignment of interventions with the WHO‐INSIPRE framework, with the vast majority aligning with the “education and life skills” domain.

#### 6.2.6 Outcomes

The predominance of curriculum‐based interventions in education settings targeted toward children is also reflected in the outcomes presented in the EGM. Across all the included studies, outcomes relating to child knowledge and awareness were reported more than any other type of outcome. Child mental health and maltreatment occurrence outcomes were also reported in a substantial number of studies. It is perhaps not surprising, given the nature of child maltreatment and its measurement in institutional contexts, that these studies mostly focused on short‐term, self‐report risk indicators for maltreatment rather than measurements of whether maltreatment actually occurred. Overall, reported outcomes tended to focus on children, and not perpetrators. Direct measures of perpetrator maltreatment behaviours, recidivism and desistence were included in only two primary studies.

Despite lowering our inclusion criteria for primary studies well‐below the RCT threshold, there was scarce evidence reporting outcomes relating to institutional safeguarding practices that may better support the prevention, disclosure and organisational responses to child maltreatment. Unfortunately, these gaps may be due to a lack of concerted, rigorous efforts at evaluation within institutional settings. Though the reasons for this are unknown, it is potentially associated with a reluctance to look closely at institutional failures and to evaluate them in a way that builds the knowledge base for prevention work in this area. The past has seen a larger research focus on maltreatment in family/home settings than in institutions. This is only now being challenged as victims of child sexual and physical abuse recount their experiences, seeking justice and restitution, sparking numerous inquiries across the world. Hopefully, this level of scrutiny and a demand for a meaningful response will translate into a growing number of safeguarding approaches that are rigorously evaluated.

Finally, only a third of the studies reported one or more outcomes that related to implementation. These included measures of feasibility, adoption, fidelity, acceptability and intervention penetration. Implementation outcomes are “the effects of deliberate and purposive actions to implement new treatments, practices, and services” and describe the result of intentional actions to deliver a policy or an intervention (see Proctor et al., 2011). Measuring implementation is important in determining how or whether an intervention was delivered as intended, information that is essential to ascertaining its effectiveness. Moreover, the effectiveness of an intervention may be compromised by insufficient attention to implementation. Measures of implementation also provide information about whether an intervention is acceptable to participants, and/or whether it is likely to be successfully adopted in real life contexts. The fact that most studies in the EGM did not report on measures of implementation is concerning, given that many studies reported on interventions which were delivered by multiple individuals (e.g., practitioners, trained staff) and across multiple study sites. This creates ample scope for variation in what gets delivered, which may impact the reliability of a study's findings.

### 6.3 Implications for future research, policy and practice

Overall, the evidence included in the EGM is sparse and of low to moderate quality. There is much need for further high‐quality research, specifically:

- Evaluating interventions in a broader range of institutions;
- In countries with the largest populations, in which the greatest prevalence of child maltreatment in institutions is likely to occur;
- Assessing interventions that focus on perpetrators and the organisational environment (as well as children);
- Studies of gender‐specific interventions or studies that disaggregate the results by gender, particularly those evaluating group‐based delivery approaches, to ascertain whether gender‐specific approaches to prevention or treatment have merit;
- Assessing interventions addressing a broader range of maltreatment types, in particular those relating to neglect and emotional abuse (i.e., not only sexual abuse);
- Assessing interventions focussed on disclosure, organisational responses and treatment (both victim/survivor and offender);
- Assessing interventions targeting perpetrators, maltreatment behaviours, recidivism and desistence and
- Assessment and reporting of implementation outcomes.

The current evidence base for interventions specifically addressing institutional child maltreatment is sparse. It is therefore difficult to assess whether an intervention which achieved some result in one location or setting will achieve that same result elsewhere. For instance:

- Could school‐based education and skills training interventions be appropriately translated to other institutions and/or other populations? For example, could the kind of child trainings which have been studied schools be delivered at Scouts? Could training for teachers be delivered to clergy?
- Could effective institutional safeguarding practices or policies be adapted to other organisational contexts and/or personnel?
- Are treatment principles for children who experienced maltreatment in other settings appropriate and effective for children who experienced maltreatment in an institutional environment? Or, are interventions for perpetrators as effective with populations of perpetrators who abused within an institutional setting?
- Given the potential for boys to respond differently to programmes than girls, should nongendered approaches be adapted into gender‐specific interventions?

Clearly, interventions that are moved from one type of setting to another may not work as well there. This highlights the importance of continuing to evaluate an intervention when it is delivered somewhere other than the setting it has been shown to be effective in. For example, if an education and training intervention which has been effective when delivered in schools, is used in a sports or recreational setting, it should be further evaluated there. Similarly, an education and training intervention for school staff to prevent child physical and emotional abuse may be transferrable to coaches and mentors in sport and recreation contexts, but would need to be evaluated with those personnel in their contexts.

### 6.4 Limitations of the EGM

The EGM involved an extensive and rigorous search for peer reviewed and grey literature, and examined over 6000 citations. We also sought relevant studies from contact with experts in the field. Despite this, it is possible that some studies relating to institutional responses to child maltreatment were missed. When screening at the title or abstract level, we may have incorrectly excluded some studies where information provided did not clearly reveal relevance to the setting or topic. Similarly, some relevant studies with crossover to settings outside the scope of our EGM, including health or clinical settings, may have been excluded on the basis of setting criteria. Snowballing techniques were not used for screening primary studies, and though we screened the primary studies included in the included systematic reviews, we did not screen all the studies in their reference lists.

Though the search terms were carefully designed, and piloted, relevant studies could still have been missed because of our included terms or because of variations in database indexing. There may have been studies in other languages that were not picked up by our search strategy, or studies that used different language/terms to describe institutional settings or child maltreatment. We will further assess the appropriateness of search terms in future updates to ensure that the search terms include relevant terminology.

Finally, due to unclear reporting, it was at times difficult to categorise intervention type, define age groups and identify the exact institutional setting where the intervention was delivered or where the abuse took place. As a result, we categorised the information based on what was available, and at times, some assumptions were necessary.

## 7 AUTHORS' CONCLUSIONS

This EGM shows a need for more high‐quality studies that assess interventions across a broad range of institutional contexts and maltreatment types. The evidence gaps are particularly evident for countries with large populations, and therefore the greatest number of children affected by child maltreatment. Few studies focussed on perpetrators or organisational environments. Evidence gaps were also identified for interventions relating to disclosure, organisational responses and treatment, and few studies were identified that assessed an intervention's impact on perpetrators' maltreatment behaviours, recidivism and desistence. There is also need for more studies to measure and report on implementation.

## CONTRIBUTIONS OF AUTHORS

The three key competency areas—content, methods and information retrieval—required for this EGM were covered by the research team members in the following way:

**Content**: Prof. Aron Shlonsky and Dr. Robyn Mildon provided content expertise in producing this EGM.

- **Prof. Aron Shlonsky** is Professor and Head of Social Work at Monash University in Melbourne, Australia. After graduating from UC Berkeley with a doctorate in social welfare and a master's degree in public health, Shlonsky was an Assistant Professor at Columbia University School of Social Work and was then Factor‐Inwentash Chair in Child Welfare at the University of Toronto Faculty of Social Work. Shlonsky is known internationally for his work in risk assessment for child maltreatment and domestic violence, child welfare practice and policy, data analytics and the use of evidence to inform practice and policy. He also has had a long‐term involvement with the Campbell Collaboration. Shlonsky has been investigator and Co‐Investigator on a large number of impact evaluations of systems that provide services to children and families and has authored and coauthored numerous other books and peer‐reviewed articles on issues related to evidence‐based child welfare practice and child maltreatment. This includes the lead authorship for multiple reports commissioned by the Australian Royal Commission into Institutional Responses to Child Sexual Abuse between 2014 and 2017.
- **Dr. Robyn Mildon** is an internationally recognised figure in the field of evidence synthesis and translation, implementation science and programme and policy evaluations in health and human services. She is the Founding Executive Director of the Centre for Evidence and Implementation (CEI), an Honorary Associate Professor with the University of Melbourne, and the inaugural Co‐Chair of the Knowledge Translation and Implementation Group with the Campbell Collaboration, an international systematic review group. Over her career, Robyn has led a number of projects focused on the generation of evidence through systematic review methods and the better use of evidence in policy and practice through the study and application of implementation science. She has a substantial track record working with multiple stakeholders to support the adoption, implementation and evaluation of effective approaches to working with children, families and their communities, and advancing the use of evidence in practice

**EGM methods**: Dr. Meghan Finch, Dr. Bianca Albers, Dr. Rebecca Featherston, and Prof. Aron Shlonsky provided methods expertise in producing this EGM:

- **Dr. Meghan Finch** has extensive experience in conducting systematic literature reviews for policy and practice use. She is a senior advisor specialising in synthesis with the Centre for Evidence and Implementation (CEI), a conjoint lecturer with the University of Newcastle, and Managing Editor of the Knowledge Translation and Implementation Group with the Campbell Collaboration, an international systematic review group. Over her career she has lead or coauthored multiple systematic reviews including Cochrane reviews. She also has a substantial track record leading large scale trials focusing on supporting the adoption, implementation and evaluation of effective approaches to implementing evidence‐based policies practices in the early childhood education and care sector.
- **Dr. Bianca Albers** is an Associate Director at Centre for Evidence and Implementation (CEI). Dr. Albers has extensive experience in conducting different types of systematic literature reviews for policy and practice use. She has been involved in the production of full systematic reviews, rapid evidence assessments, scoping reviews, reviews of reviews and evidence gap maps—commissioned among others by the VIC Department of Health and Human Services Australia (EGM on child and family services); the NSW Department of Family and Community Services (Australia—EGM on OOHC); WHO (full systematic reviews on Community Health Worker Programmes), and The Royal Commission into Institutional Responses to Child Sexual Abuse (multiple projects).
- **Dr. Rebecca Featherston** is a Research Fellow in the Department of Social Work at Monash University. She holds a PhD in Biosciences and a Masters of Social Work from the University of Melbourne. Dr. Featherston has been involved in a range of social and child welfare related research projects, partnering with government and other organisations. These projects have included programme evaluations, multiple systematic evidence syntheses and quantitative data analysis. She has substantial experience in conducting systematic literature reviews, including full systematic reviews, scoping reviews, reviews of reviews and evidence gap maps.

### INFORMATION RETRIEVAL

The production of this EGM was supported by a Monash University librarian. The daily management of the EGM production, including coordination of multiple, parallel work processes, stakeholder engagement and communication and other responsibilities was shared by Caroline Fiennes, Dr. Meghan Finch and Dr. Rebecca Featherston. The screening of literature, quality assessments and coding of studies and comparable research tasks were undertaken by Sangita Chakraborty, Ludvig Bjørndal, Rebecca Schachtman, Taoran Yang, Dr. Rebecca Featherston and Dr. Meghan Finch. David Taylor developed the visual EGM. In questions that required particular methodical and/or statistical expertise, the team was supported by Prof. Aron Shlonsky.

## DECLARATIONS OF INTEREST

**Porticus** has supported, and currently supports, efforts among its grantees to improve organisational safeguarding of children. Specifically, Porticus:

- Requires from all its grantees to have a safeguarding policy;
- Works with some grantees to further develop interventions that can make the organisations safer.

These projects are conducted in collaboration with both faith‐based organisations and non‐faith‐based organisations. To ensure that all standards for the production of a Campbell EGM were met, Porticus was not involved in any technical steps taken to produce the EGM, including information retrieval, data analysis and reporting of findings.

Porticus commissioned this EGM to further support its own and others' ongoing work to enhance organisational safeguarding. More detail about the project that produced this research, and other products from it, are at: www.giving-evidence.com/csa.

**Prof. Aron Shlonsky, Dr. Robyn Mildon and Ms. Bianca Albers** have coauthored multiple publications commissioned by the Australian Royal Commission into Institutional Responses to Child Sexual Abuse including the following:

- **Shlonsky A., Albers B**., Paterson N., Condron P., Morrissey F., Romey G. Rapid evidence review on the availability, modality and effectiveness of psychosocial support services for child and adult victims and survivors of child sexual abuse. Royal Commission into Institutional Responses to Child Sexual Abuse, Commonwealth of Australia; 2017
- **Shlonsky A., Albers B**., Tolliday D., Wilson S. J., Norvell J., Kissinger L. Rapid evidence assessment: Current best evidence in the therapeutic treatment of children with problem or harmful sexual behaviours, and children who have sexually offended. Sydney: Royal Commission into Institutional Responses to Child Sexual Abuse, Commonwealth of Australia; 2017
- Valentine K., Katz I., Smyth C., Bent C., Rinaldis S., Wade C., **Albers B**. Key elements of child safe organisations–research study final report. Sydney: Royal Commission into Institutional Responses to Child Sexual Abuse, Commonwealth of Australia; 2016
- Parenting Research Centre, **Albers B., Mildon R**. Implementation best practice: A rapid evidence assessment. Royal Commission into Institutional Responses to Child Sexual Abuse, Commonwealth of Australia; 2016.
- South S., **Shlonsky A., Mildon R**. Scoping review: Evaluations of out‐of‐home care practice elements that aim to prevent child sexual abuse. Royal Commission into Institutional Responses to Child Sexual Abuse, Commonwealth of Australia; 2014.
- South S., **Shlonsky A., Mildon, R**. Scoping review: Evaluations of pre‐employment screening practices for child‐related work that aim to prevent child sexual abuse. Royal Commission into Institutional Responses to Child Sexual Abuse, Commonwealth of Australia; 2015.

### Plans for updating the EGM

This EGM will be updated on a biennial basis.

## Differences between protocol and review

1. Our affiliated university libraries were unable to access the Family and Society Studies Worldwide and SocIndex academic databases. On the recommendation of our librarian and with approval of the author team, the following databases were searched as suitable replacements:

- Informit Families and Society Collection (Australian)—Covers subjects related to family and community, social services and public welfare, family law, and culture and institutions. Contains journals, books, and reports.
- Sociological Abstracts—Proquest index to international literature in sociology and related disciplines in the social and behavioural sciences.
- Sociology Source Ultimate—Subjects include gender identity, marriage and family, demographics, political sociology, religion and socio‐cultural anthropology.

2. Interventions were further coded using the INSPIRE categories outlined by the WHO.
3. The included studies lists of all included systematic reviews underwent title and abstract screening in order to find further primary and/or systematic review studies.
4. Multiple wording changes and correction of typographical errors have been made on sections that relate to the protocol, including the EGM framework, with the aim to improve clarity and consistency.

## SOURCES OF SUPPORT

### Internal sources

- No sources of support provided.

### External sources

- Porticus, Other.

This EGM was funded by Porticus, an international organisation managing and developing the philanthropic programmes of charitable entities established by Brenninkmeijer family entrepreneurs. Porticus is involved with and fund a broad range of social service activities, including to both faith‐based organisations, and organisations unrelated to religious institutions.

## APPENDIX A

### ACADEMIC DATABASE SEARCH TERMINOLOGY

#### Database(s): PsycINFO 1806

**Table:**

#	Searches
1	(adolescence 13 17 yrs or childhood birth 12 yrs or infancy 2 23 mo or neonatal birth 1 mo or preschool age 2 5 yrs or school age 6 12 yrs).ag
	(Infant or infants or infancy or Child or childs or children or childrens or childhood or Minors or Minor person* or minor people or Toddler or toddlers or baby or babies or Adolescent or adolescents or adolescence or teen or teens or teenage or teenaged or teenager or teenagers or young person or young persons or young people or youth or youths or juvenile or juveniles or boy or boys or girl or girls).mp
	1 or 2
	child neglect/or child abuse
	(neglect* or abandon* or maltreat* or mistreat* or ill treat* or illtreat* or harm or harmful or harmed or vulnerab* or abus* or assault or problem sexual behavi*).mp
	4 or 5
	meta analysis/or "systematic review"/
	(metaanal* or meta anal* or (systematic adj2 review*) or systematic synthesis).mp. or (meta analysis or metasynthesis or "systematic review").md.
	randomized controlled trials/
	(RCT or randomi* or (random* adj3 (assign* or allocat*)) or blinded or double blind* or doubleblind*).mp.
	quasi experimental methods/
	time series/
	(Quasi experiment* or quasiexperiment* or step wedge or "difference in difference*" or synthetic control group or covariate matching or propensity score or doubly robust estimat* or regression adjustment estimate* or regression discontinuity or instrumental variable* estimate* or time series or timeseries or before after or before‐after or pre post).mp
	7 or 8 or 9 or 10 or 11 or 12 or 13
	intervention/
	(intervention or interventions or prevent* or treatment or treatments or program or programs or programme or programmes or policy or policies).mp
	Health Education/or Mass Media/or Prevention/or Social Media/or Communications Media/
	professional development/or continuing education/or inservice teacher education/or inservice training/or training/or professional training/or mental health inservice training/or professional certification/or professional competence/or professional standards/
	(Human Resource Management or Job Applicant Screening or Personnel Recruitment or employ* screening or pre employ* screening).mp.
	15 or 16 or 17 or 18 or 19
	((residential and (care or institution)) or (oohc or (out of home adj3 care*)) or (foster* adj2 (youth or child* or infant*)) or (child* adj2 "looked after") or orphanage or (child* adj2 home) or (child* adj2 institution) or pre school or preschool or "pre k" or kindergarten or day care or daycare or nursery or nurseries or play group* or playgroup* or ((after school or afterschool or out of school) and program*) or camp or camps or club or clubs or (child* and (center* or centre* or institution*)) or (institution* adj2 (faith based religious or care or setting)) or church* or temple* or mosque*).mp.
	exp correctional institutions/
	junior high schools/or technical schools/or middle schools/or nursery schools/or elementary schools/or nongraded schools/or military schools/or high schools/or charter schools/or boarding schools/or schools/or institutional schools/
	21 or 22 or 23
25	3 and 6 and 14 and 20 and 24

#### Database(s): Medline 1946–present

**Table:**

#	
1	adolescent/or exp child/or exp infant/
	(Infant or infants or infancy or Child or childs or children or childrens or childhood or Minors or Minor person* or minor people or Toddler or toddlers or baby or babies or Adolescent or adolescents or adolescence or teen or teens or teenage or teenaged or teenager or teenagers or young person or young persons or young people or youth or youths or juvenile or juveniles or boy or boys or girl or girls).mp.
	1 or 2
	child abuse/or child abuse, sexual/
	(neglect* or abandon* or maltreat* or mistreat* or ill treat* or illtreat* or harm or harmful or harmed or vulnerab* or abus* or assault or problem sexual behavi*).mp.
	4 or 5
7	meta‐analysis/or "systematic review"/
8	double‐blind method/or meta‐analysis as topic/or single‐blind method/
9	(metaanal* or meta anal* or (systematic adj2 review*) or systematic synthesis).mp.
10	(RCT or randomi* or (random* adj3 (assign* or allocat*)) or blinded or double blind* or doubleblind*).mp.
11	(Quasi experiment* or quasiexperiment* or step wedge or "difference in difference*" or synthetic control group or covariate matching or propensity score or doubly robust estimat* or regression adjustment estimate* or regression discontinuity or instrumental variable* estimate* or time series or timeseries or before after or before‐after or pre post).mp.
12	Randomized Controlled Trial/
13	7 or 8 or 9 or 10 or 11 or 12
14	(intervention or interventions or prevent* or treatment or treatments or program or programs or programme or programmes or policy or policies).mp.
15	Health Education/or Mass Media/or Prevention Prevention/or Social Media/or Communications Media/
16	education, continuing/or teacher training/or inservice training/or staff development/
17	Professional Competence/
18	(Human Resource Management or Job Applicant Screening or Personnel Recruitment or employ* screening or pre employ* screening or professional standard* or professional development or professional training).mp.
19	14 or 15 or 16 or 17 or 18
20	((residential and (care or institution)) or (oohc or (out of home adj3 care*)) or (foster* adj2 (youth or child* or infant*)) or (child* adj2 "looked after") or orphanage or (child* adj2 home) or (child* adj2 institution) or pre school or preschool or "pre k" or kindergarten or day care or daycare or nursery or nurseries or play group* or playgroup* or ((after school or afterschool or out of school) and program*) or camp or camps or club or clubs or (child* and (center* or centre* or institution*)) or (institution* adj2 (faith based religious or care or setting)) or church* or temple* or mosque*).mp.
21	Prisons/or (correctional institution* or gaol* or jail*).mp.
22	Schools/
23	school*.mp.
24	20 or 21 or 22 or 23
**25**	**3 and 6 and 13 and 19 and 24**

#### CINAHL

**Table:**

#	Query
S1	(MH "Child") OR (MH "Adolescence") OR (MH "Infant+") OR (MH "Child, Preschool")
S2	(Infant or infants or infancy or Child or childs or children or childrens or childhood or Minors or Minor person* or minor people or Toddler or toddlers or baby or babies or Adolescent or adolescents or adolescence or teen or teens or teenage or teenaged or teenager or teenagers or young person or young persons or young people or youth or youths or juvenile or juveniles or boy or boys or girl or girls)
S3	S1 OR S2
S4	(MH "Child Abuse") OR (MH "Child Abuse, Sexual") OR (MH "Neglect (Omaha)")
S5	(neglect* or abandon* or maltreat* or mistreat* or ill treat* or illtreat* or harm or harmful or harmed or vulnerab* or abus* or assault or problem sexual behavi*)
S6	S4 OR S5
S7	(MH "Meta Analysis") OR (MH "Meta Synthesis")
S8	(MH "Systematic Review")
S9	(metaanal* or "meta anal*" or (systematic n2 review*) or "systematic synthesis" or metasynthesis)
S10	(MH "Randomized Controlled Trials")
S11	(MH "Double‐Blind Studies")
S12	(RCT or randomi* or (random* n3 (assign* or allocat*)) or blinded or double blind* or doubleblind*)
S13	(MH "Quasi‐Experimental Studies")
S14	(MH "Time Series")
S15	("Quasi experiment*" or quasiexperiment* or "step wedge" or "difference in difference*" or "synthetic control group" or "covariate matching" or "propensity score" or "doubly robust estimat*" or "regression adjustment estimate*" or "regression discontinuity" or "instrumental variable* estimate*" or "time series" or timeseries or "before after" or "before‐after" or "pre post")
S16	S7 OR S8 OR S9 OR S10 OR S11 OR S12 OR S13 OR S14 OR S15
S17	(intervention or interventions or prevent* or treatment or treatments or program or programs or programme or programmes or policy or policies)
S18	(MH "Health Education")
S19	(MH "Communications Media")
S20	(MH "Social Media")
S21	(MH "Professional Development")
S22	(MH "Education, Continuing")
S23	(MH "Professional Competence")
S24	("Human Resource Management" or "Job Applicant Screening" or "Personnel Recruitment" or "employ* screening" or "pre employ* screening" or training or "professional standard*")
S25	S17 OR S18 OR S19 OR S20 OR S21 OR S22 OR S23 OR S24
S26	((residential and (care or institution)) or (oohc or ("out of home" n3 care*)) or (foster* n2 (youth or child* or infant*)) or (child* n2 "looked after") or orphanage or (child* n2 home) or (child* n2 institution) or "pre school" or preschool or "pre k" or kindergarten or "day care" or daycare or nursery or nurseries or "play group*" or playgroup* or (("after school" or afterschool or "out of school") and program*) or camp or camps or club or clubs or (child* and (center* or centre* or institution*)) or (institution* n2 ("faith based religious" or care or setting)) or church* or temple* or mosque*)
S27	(MH "Correctional Facilities")
S28	gaol* or jail* or "correctional institution*"
S29	(MH "Schools, Elementary") OR (MH "Schools, Middle") OR (MH "Schools, Nursery") OR (MH "Schools, Secondary") OR (MH "Schools")
S30	S26 OR S27 OR S28 OR S29
**S31**	**S3 AND S6 AND S16 AND S25 AND S30**

#### ERIC, Sociological Abstracts & Proquest Dissertations and Theses

noft(Infant OR infants OR infancy OR Child OR childs OR children OR childrens OR childhood OR Minors OR "Minor person*" OR "minor people" OR Toddler OR toddlers OR baby OR babies OR Adolescent OR adolescents OR adolescence OR teen OR teens OR teenage OR teenaged OR teenager OR teenagers OR "young person" OR "young persons" OR "young people" OR youth OR youths OR juvenile OR juveniles OR boy OR boys OR girl OR girls) AND noft(neglect* OR abandon* OR maltreat* OR mistreat* OR "ill treat*" OR illtreat* OR harm OR harmful OR harmed OR vulnerab* OR abus* OR assault OR "problem sexual behavi*") AND noft(metaanal* OR "meta anal*" OR (systematic near/2 review*) OR "systematic synthesis" OR metasynthesis OR RCT OR randomi* OR (random* near/3 (assign* OR allocat*)) OR blinded OR "double blind*" OR doubleblind* OR "Quasi experiment*" OR quasiexperiment* OR "step wedge" OR "difference in difference*" OR "synthetic control group" OR "covariate matching" OR "propensity score" OR "doubly robust estimat*" OR "regression adjustment estimate*" OR "regression discontinuity" OR "instrumental variable* estimate*" OR "time series" OR timeseries OR "before after" OR "before‐after" OR "pre post") AND noft(intervention OR interventions OR prevent* OR treatment OR treatments OR program OR programs OR programme OR programmes OR policy OR policies OR "Health Education" OR "Mass Media" OR Prevention OR "Social Media" OR "Communications Media" OR training OR "professional development" OR "continuing education" OR training OR "professional certification" OR "professional competence" OR "professional standards" OR "Human Resource Management" OR "Job Applicant Screening" OR "Personnel Recruitment" OR "employ* screening" OR "pre employ* screening") AND noft(((residential AND (care OR institution)) OR (oohc OR ("out of home" near/3 care*)) OR (foster* near/2 (youth OR child* OR infant*)) OR (child* near/2 "looked after") OR orphanage OR (child* near/2 home) OR (child* near/2 institution) OR "pre school" OR preschool OR "pre k" OR kindergarten OR "day care" OR daycare OR nursery OR nurseries OR "play group*" OR playgroup* OR (("after school" OR afterschool OR "out of school") AND program*) OR camp OR camps OR club OR clubs OR (child* AND (center* OR centre* OR institution*)) OR (institution* near/2 ("faith based religious" OR care OR setting)) OR church* OR temple* OR mosque*))

#### SCOPUS

TITLE‐ABS (infant OR infants OR infancy OR child OR childs OR children OR childrens OR childhood OR minors OR "Minor person*" OR "minor people" OR toddler OR toddlers OR baby OR babies OR adolescent OR adolescents OR adolescence OR teen OR teens OR teenage OR teenaged OR teenager OR teenagers OR "young person" OR "young persons" OR "young people" OR youth OR youths OR juvenile OR juveniles OR boy OR boys OR girl OR girls) AND TITLE‐ABS (neglect* OR abandon* OR maltreat* OR mistreat* OR "ill treat*" OR illtreat* OR harm OR harmful OR harmed OR vulnerab* OR abus* OR assault OR "problem sexual behavi*") AND TITLE‐ABS (metaanal* OR "meta anal*" OR (systematic W/2 review*) OR "systematic synthesis" OR metasynthesis OR rct OR randomi* OR (random* W/3 (assign* OR allocat*)) OR blinded OR "double blind*" OR doubleblind* OR "Quasi experiment*" OR quasiexperiment* OR "step wedge" OR "difference in difference*" OR "synthetic control group" OR "covariate matching" OR "propensity score" OR "doubly robust estimat*" OR "regression adjustment estimate*" OR "regression discontinuity" OR "instrumental variable* estimate*" OR "time series" OR timeseries OR "before after" OR "before‐after" OR "pre post") AND TITLE‐ABS (intervention OR interventions OR prevent* OR treatment OR treatments OR program OR programs OR programme OR programmes OR policy OR policies OR "Health Education" OR "Mass Media" OR prevention OR "Social Media" OR "Communications Media" OR training OR "professional development" OR "continuing education" OR training OR "professional certification" OR "professional competence" OR "professional standards" OR "Human Resource Management" OR "Job Applicant Screening" OR "Personnel Recruitment" OR "employ* screening" OR "pre employ* screening") AND TITLE‐ABS (((residential AND (care OR institution)) OR (oohc OR ("out of home" W/3 care*)) OR (foster* W/2 (youth OR child* OR infant*)) OR (child* W/2 "looked after") OR orphanage OR (child* W/2 home) OR (child* W/2 institution) OR "pre school" OR preschool OR "pre k" OR kindergarten OR "day care" OR daycare OR nursery OR nurseries OR "play group*" OR playgroup* OR (("after school" OR afterschool OR "out of school") AND program*) OR camp OR camps OR club OR clubs OR (child* AND (center* OR centre* OR institution*)) OR (institution* W/2 ("faith based religious" OR care OR setting)) OR church* OR temple* OR mosque*))

#### INFORMIT

Infant or infants or infancy or Child or childs or children or childrens or childhood or Minors or “Minor person*” or “minor people” or Toddler or toddlers or baby or babies or Adolescent or adolescents or adolescence or teen or teens or teenage or teenaged or teenager or teenagers or “young person” or “young persons” or “young people” or youth or youths or juvenile or juveniles or boy or boys or girl or girls AND neglect* or abandon* or maltreat* or mistreat* or “ill treat*” or illtreat* or harm or harmful or harmed or vulnerab* or abus* or assault or “problem sexual behavi*” AND metaanal* or “meta anal*” or “systematic review*” or “systematic synthesis” or metasynthesis or RCT or randomi* or (random* and (assign* or allocat*)) or blinded or “double blind*” or doubleblind* or “Quasi experiment*” or quasiexperiment* or “step wedge” or "difference in difference*" or “synthetic control group” or “covariate matching” or “propensity score” or “doubly robust estimat*” or “regression adjustment estimate*” or “regression discontinuity” or “instrumental variable* estimate*” or “time series” or timeseries or “before after” or “before‐after” or “pre post” ANDintervention or interventions or prevent* or treatment or treatments or program or programs or programme or programmes or policy or policies or “Health Education” or “Mass Media” or Prevention or “Social Media” or “Communications Media” or training or “professional development” or “continuing education” or training or “professional certification” or “professional competence” or “professional standards” or “Human Resource Management” or “Job Applicant Screening” or “Personnel Recruitment” or “employ* screening” or “pre employ* screening” AND ((residential and (care or institution)) or (oohc or (“out of home” and care*)) or (foster* and (youth or child* or infant*)) or (child* and "looked after") or orphanage or (child* and home) or (child* and institution) or “pre school” or preschool or "pre k" or kindergarten or “day care” or daycare or nursery or nurseries or “play group*” or playgroup* or ((“after school” or afterschool or “out of school”) and program*) or camp or camps or club or clubs or (child* and (center* or centre* or institution*)) or (institution* and (“faith based religious” or care or setting)) or church* or temple* or mosque*)

#### Campbell Collaboration Library

"child maltreatment" OR "child abuse"

### EGM Subject Matter Expert Group

**Table:**

	Name	Organisational affiliation	Country
**1**	Prof. Leah Bromfield	Australian Centre for Child Protection, University of South Australia	AUS
**2**	Prof. Daryl Higgins	Institute of Child Protection Studies, Australian Catholic University	AUS
**3**	Prof. Ben Mathews	Director, Childhood Adversity Research Program, Faculty of Health, Queensland University of Technology	AUS
**4**	Emeritus Prof. Stephen Smallbone	Griffith University, Australia	AUS
**5**	Mathieu Lacambre/Wayne Bodkin	Department of Forensic Psychiatry, University Hospital Montpellier	F
**6**	Dr. Karen Devries/Louise Knight	London School of Hygiene and Tropical Medicine, U.K.	UK
**7**	Donald Findlater/Stuart Allardyce	“Stop It Now”/Lucy Faithfull Foundation, U.K.	UK
**8**	Honorary Prof. Derek E. Perkins	School of Law, Royal Holloway University of London, U.K.	UK
**9**	Prof. Richard Wortley & Lorraine Sherr	University College London, U.K.	UK
**10**	Francisca Meinck	Oxford University	UK
**11**	Prof. Elizabeth J. Letourneau	Johns Hopkins Bloomberg School of Public Health	US
**12**	Prof. Jennie Noll	Penn State College of Health and Human Development	US
**13**	Dr. Bruce Taylor	NORC, University of Chicago	US
**14**	Nicole Williams	Maestral International	INT
**15**	Kerry Albright	UNICEF	INT
**16**	Claire Feinstein	Save the Children	INT

### EGM Coding Scheme

#### Study characteristics

1. **Study design**
  1.1. Systematic review
  1.2. RCT (including cluster RCT)
  1.3. QED
  1.4. Unclear

2. **Status of study**
  2.1. Completed
  2.2. Ongoing
  2.3. Unclear
3. **Systematic review quality**
  3.1. Critically Low/Low
  3.2. Moderate
  3.3. High

4. **Primary study quality**
  4.1. Low
  4.2. Some concerns
  4.3. High

#### Population

5. **Target population**
  5.1. Child victims
  5.2. Child offenders
  5.3. Institutional adult members/care providers
  5.4. Adult perpetrators
  5.5. Mixed
  5.6. Unclear

6. **Child age group(s)**
  6.1. Prenatal
  6.2. Infancy (0–23 months)
  6.3. Early childhood (24 months–5 years)
  6.4. Middle childhood (6–11 years)
  6.5. Early adolescence (12–14)
  6.6. Late adolescence (15–17)
  6.7. Mixed
  6.8. Unclear
7. **Child risk status**
  7.1. Not at risk population
  7.2. At risk population
  7.3. Exposed population
  7.4. Mixed population
  7.5. Unclear
8. **Type of maltreatment**
  8.1. Neglect
  8.2. Physical abuse
  8.3. Sexual abuse
  8.4. Emotional abuse
  8.5. Mixed
  8.6. Unclear

#### Intervention

9. **Intervention type**
  9.1. Prevention
  9.2. Disclosure
  9.3. Response
  9.4. Treatment
  9.5. Other: _________________
  9.6. Mixed
  9.7. Unclear
10. **Intervention target**
  10.1. Child victim
  10.2. Child offender
  10.3. Adult perpetrator
  10.4. Organisational leadership
  10.5. Organisational staff
  10.6. Caregiver/parent
  10.7. Other: _________________
  10.8. Mixed
  10.9. Unclear
11. **Delivery mode**
  11.1. Individual
  11.2. Group
  11.3. Other: ______________________
  11.4. Mixed
  11.5. Unclear

#### Setting

12. **Geography (following** WHO Regions**)**
  12.1. Africa
  12.2. Americas
  12.3. South‐East Asia
  12.4. Europe
  12.5. Eastern Mediterranean
  12.6. Western Pacific
13. **Institutional setting**
  13.1. Early childhood settings (e.g., kindergarten, pre‐school, centre‐based daycare)
  13.2. School (e.g., primary/elementary, secondary/high, before/after school care)
  13.3. Sports clubs, recreational settings (e.g., dance/drama and music studios)
  13.4. Churches/religious institutions
  13.5. Summer/vacation camps
  13.6. Out of home care settings (e.g., orphanages, residential care, foster care)
  13.7. Detention centres/juvenile justice settings
  13.8. Rescue centres
  13.9. Primary health care facilities
  14.0. Secondary health care facilities
  14.1. Other: ___________________
  14.2. Mixed
  14.3. Unclear

#### Outcomes

14. **Institutional safeguarding practice**
  14.1. Institutional culture
  14.2. Operational practice
  14.3. Environmental changes
15. **Disclosure**
  15.1. Disclosure rates
16. **Child safety**
  16.1. Maltreatment occurrence/reoccurrence
17. **Child cognitive functioning**
  17.1. Language development
  17.2. Pre‐academic skills (e.g., literacy/numeracy)
  17.3. Academic achievement
  17.4. Problem solving skills
  17.5. School engagement/school attachment
18. **Child physical health and development**
  18.1. Normative standards for health and development
  18.2. Gross motor and fine motor skills
  18.3. Overall health
  18.4. BMI
  18.5. Health related risk‐avoidance behaviour
19. **Child mental health**
  19.1. Self‐control, emotional management, and expression
  19.2. Internalising symptoms
  19.3. Externalising symptoms
  19.4. Traumatic stress symptoms
  19.5. Self‐esteem
  19.6. Emotional intelligence
  19.7. Self‐efficacy
  19.8. Motivation
  19.9. Pro‐social behaviour
20. **Child social functioning**
  20.1. Social competence
  20.2. Social skills
  20.3. Attachment and caregiver relationships
  20.4. Adaptive behaviours
  20.5. Social connections and relationships
21. **Child knowledge and awareness**
  21.1. Knowledge about, and responses to (i.e., protective skills), child maltreatment behaviour/offending
  21.2. Risk awareness and risk targeting behaviour
22. **Child or youth offender outcomes**
  22.1. Desistance
  22.2. Recidivism
  22.3. Maltreatment behaviours
  22.4. Other: ______________________
23. **Adult perpetrator outcomes**
  23.1. Recidivism
  23.2. Desistance
  23.3. Maltreatment behaviours
  23.4. Other: ______________________
24. **Parent/caregiver outcomes**
  24.1. Behaviour/knowledge/attitudes
  24.2. Other: ______________________
25. **Implementation outcomes**
  25.1. Fidelity
  25.2. Other **________________________**
26. **Other outcomes**

27.1. Other ________________________

### Excluded studies with reasons for exclusion

**Table:**

Source	Reason for exclusion
1. Abatemarco DJGRS, LaNoue MD, Phlig RT, Slovin SR, Healy JA, Kairys S. Practicing safety: A quality improvement intervention to test tools to enhance pediatric psychosocial care for children 0‐3 years. *Primary Health Care Research & Development*, 2018;19(4):365–377.	Wrong intervention
2. Abebe KZJKA, Ciaravino S, Ripper L, Paglisotti T, Morrow SE, Grafals M, Van Dusen C, Miller E. A cluster‐randomized trial of a middle school gender violence prevention program design, rationale and sample characteristics. *Contemporary Clinical Trials*, 2017;62:11–20.	Wrong setting
3. Ackerman ARK, Bilal. Assessing reporting patterns of child sexual abuse within the Catholic Church using discontinuities in model parameter timeseries. *Social Science Research*, 2012;41(2):253–262.	Wrong study design
4. Alexander MA. Sexual offender treatment efficacy revisited. *Sexual Abuse: Journal of Research & Treatment*, 1999;11(2):101–116.	Wrong intervention
5. Allen J, Vostanis P. The impact of abuse and trauma on the developing child: An evaluation of a training program for foster carers and supervising socila workers. *Adoption & Fostering*, 2005;29(3):68–81.	Wrong setting
6. Anderson‐Varney T. The evaluation of a personal safety curriculum for preschoolers. Master's Thesis, Michigan state University 1988.	Unable to contact author
7. Ashley D & Fox K. *The role of the health sector in violence prevention and management*. Ending Violence in Childhood Global Report 2017 2017.	Wrong study design
8. Azzopardi C, Eirich R, Rash CL, MacDonald S, Madigan S. A meta‐analysis of the prevalence of child sexual abuse disclosure in forensic settings. *Child Abuse & Neglect*, 2019;93:291. https://doi.org/10.1016/j.chiabu.2018.11.020	Wrong setting
9. Baiocchi M, Omondi B, Langat N, Boothroyd DB, Sinclair J, Pavia L, Mulinge M, Githua O, Golden NH & Sarnquist C. A behaviour‐based Intervention that prevents Sexual Assault: The result of a matched‐pairs, cluster‐randomized study in Nairobi, Kenya. *Prevention Science*, 2017;18(7):818*–*827. https://doi.org/10.1007/s11121-016-0701-0	Wrong outcome measures
10. Barlow J, Simkiss D, Stewart‐Brown S. Intervention to prevent or ameliorate child physical abuse and neglect: Findings from a systematic review of reviews. *Journal of Children's Services* 2006;1(3):6–28.	Wrong setting
11. Bartelink C, van Yperen TA, ten Berge IJ. Deciding on child maltreatment: A literature review on methods that improve decision‐making. *Child Abuse & Neglect*, 2015;49:142–153. https://doi.org/10.1016/j.chiabu.2015.07.002	Wrong setting
12. Barth R, Yeaton J, Winterfelt N. Psychoeducational groups with foster parents of sexually abused children. *Child and Adolescent Social Work Journal*, 1994;11(5):405–424.	Wrong setting
13. Baux‐Cazal L, Gokalsing E, Anadeo S & Messiah A. Suicidal Behaviour Prevention for Children Under Age 13: A systematic review. L'Encephale: Revue de psychiatrie clinique biologique et therapeutique 2017;43(3):273–280.	Wrong outcome measures
14. Beattie TS, Bhattacharjee P, Isac S, Davey C, Javalkar P, Nair S, Thalinja R, Sudhakar G, Collumbien M, Blanchard JF, Watts C, Moses S & Heise:L. Supporting adolescent girls to stay in school, reduce child marriage and reduce entry into sex work as HIV risk prevention in north Karnataka, India: protocol for a cluster randomised controlled trial. *BMC Public Health*, 2015;15(292). https://doi.org/10.1186/s12889-015-1623-7	Wrong intervention
15. Bencuva NL. Acceptance and mindfulness treatment for children adopted from foster care. Dissertation Abstracts International: The Sciences and Engineering, 2014;75.	Wrong setting
16. Bernard K, Dozier M, Bick J & Gordon KM. Intervening to enhance cortisol regulation among children at risk for neglect: Results of a randomized clinical trial. *Development & Psychopathology*, 2015;27(3):829–841. https://doi.org/10.1017/S095457941400073X	Wrong setting
17. Berrick JD & Barth RP. Child sexual abuse prevention: Research review and recommendations. *Social Work Research & Abstracts*, 1992;28(4):6–15. https://doi.org/10.1093/swra/28.4.6	Wrong study design
18. Berry K & Hunt CJ. Evaluation of an intervention program for anxious adolescent boys who are bullied at school. Journal of Adolescent Health, 2009;45(4):376–382. https://doi.org/10.1016/j.jadohealth.2009.04.023	Child maltreatment type out of scope
19. Biehal N. Maltreatment in foster care: A review of the evidence. *Child Abuse Review*, 2014;23(1):48–60. https://doi.org/10.1002/car.2249	Wrong intervention
20. Bjornseth I & Szabo A. Sexual Violence Against Children in Sports and Exercise: A Systematic Literature Review. Journal of Child Sexual Abuse, 2018;27(4):365–385. https://doi.org/10.1080/10538712.2018.1477222	Wrong outome measures
21. Black BM, Weisz AN & Dheeshana SJ. Dating violence and sexual assault prevention with african american middle schoolers: Does group gender composition impact dating violence attitudes? *Child & Youth Services*, 2012;33(2):158–173.	Wrong setting
22. Blair K. *Parent‐child interaction therapy and resilience within child welfare*. Dissertation Abstracts International Section A: Humanities and Social Sciences 2018;79.	Wrong outcome measures
23. Bonell C, Fletcher A, Fitzgerald‐Yau N, hale D, Allen E, Elbourne D, Jones R, Bond L, WIggins M, Miners A, Legood R, Scott S, Christie D & Viner R. Initiating change locally in bullying and aggression through the school environment (INCLUSIVE): A pilot randomised controlled trial. *Health Technology Assessment (Winchester, England)*, 2015;19(53):1–109. https://doi.org/10.3310/hta19530	Wrong intervention
24. Boulton MJ & Boulton L. Modifying self‐blame, self‐esteem, and disclosure through a cooperative cross‐age teaching intervention for bullying among adolescents. *Violence & Victims*, 2017 August 1;32(4):609–626. https://doi.org/10.1891/0886-6708.VV-D-15-00075	Wrong intervention
25. Breckenridge J & Flax G. Service and support needs of specific population groups that have experienced child sexual abuse. Royal Commission into Institutional Responses to Child Sexual Abuse, Sydney 2016.	Wrong outcome measures
26. Bruce J, McDermott JM, Fisher PA & Fox NA. Using behavioral and electrophysiological measures to assess the effects of a preventive intervention: A preliminary study with preschool‐aged foster children. *Prevention Science*, 2009;10(2):129–140. https://doi.org/10.1007/s11121-008-0115-8	Wrong intervention
27. Burgess E & Wurtele S. Enhancing parent‐child communication about sexual abuse: A pilot study. *Child Abuse & Neglect*, 1998;22(11):1167–1175.	Wrong setting
28. Cantone E, Piras AP, Vellante M & Preti A. Interventions on bullying and cyberbullying in schools: A systematic review. *Clinical Practice & Epidemiology in Mental Health*, 2015;11:58–76.	Child maltreatment type out of scope
Cardazone 2014. https://doi.org/10.2174/1745017901511010058	
29. Cardazone G, Sy AU, Chik I & Corlew LK. Mapping One Strong 'Ohana: Using Network Analysis and GIS to Enhance the Effectiveness of a Statewide Coalition to Prevent Child Abuse and Neglect. *American Journal of Community Psychology*, 2014;53(3–4):346–356. https://doi.org/10.1007/s10464-014-9641-7	Wrong study design
30. Carr A, Duff H & Craddock F. A systematic review of reviews of the outcome of severe neglect in underresourced childcare institutions. *Trauma Violence & Abuse*, 2018;21(3):484–497. https://doi.org/10.1177/1524838018777788	Wrong study design
31. Casler L. The effects of extra tactile stimulation on a group of instituionalized infants. *Genetic Psychology Monographs*, 1965;71:137–175.	Wrong intervention
32. Casler L. The effects of supplimentary verbal stimulation on a group of institutionalized infants. *Journal of Child Psychology & Psychiatry*, 1965;6:19–27.	Wrong intervention
33. Chamroonsawasdi K, Suparp J, Kittipichai W & Khajornchaikul P. gender roles, physica and sexual violence prevention in primary extend to secondary school in Samutsakorn province, Thailand. *Journal of the Medical Association of Thailan*d, 2011;93(3):358–365.	Wrong intervention
34. Chen CC, Hamm JV, Farmer TW, Lambert K, Mehtaji M. Exceptionality and Peer Victimization Involvement in Late Childhood. *Remedial & Special Education*, 2015;36(5):312–324.	Wrong outcome measures
Child 2014. https://doi.org/10.1177/0741932515579242	
35. Child JC, Naker D, Horton J, Walakira EJ & Devries KM. Responding to abuse: Children's experiences of child protection in a central district, Uganda. *Child Abuse & Neglect*, 2014;38(10):1647–1658. https://doi.org/10.1016/j.chiabu.2014.06.009	Wrong study design
36. Chiodo D, Crooks CV, Wolfe DA, McIsaac C, Hughes R & Jaffe PG. Longitudinal prediction and concurrent functioning of adolescent girls demonstrating various profiles of dating violence and victimization. *Prevention Science*, 2012;13(4):350–359. https://doi.org/10.1007/s11121-011-0236-3	Wrong setting
37. Cid A. Interventions using Regular Activities to Engage High‐risk School‐age Youth: A Review of After‐school Programmes in Latin America and the Caribbean. Ending Violence in Childhood Global Report 2017.	Wrong study design
38. Clarkson S, Axford N, Berry V, Edwards RT, Bjornstad G, Wrigley Z, Charles J, Hoare Z, Ukoumunne OC, Matthews J & Hutchings J. Effectiveness and micro‐costing of the KiVa school‐based bullying prevention programme in Wales: Study protocol for a pragmatic definitive parallel group cluster randomised controlled trial. *BMC Public Health*, 2016;16(104). https://doi.org/10.1186/s12889-016-2746-1	Wrong setting
39. Cohen JA, Deblinger E, Mannarino AP & Steer RA. A Multisite, randomized controlled trial for children with sexual abuse‐related PTSD symtpoms. *Journal of the American Academy of Child & Adolescent Psychiatry*, 2004;43(4):393–402. https://doi.org/10.1097/00004583-200404000-00005	Wrong setting
40. Cohen JA, Mannarino AP & Knudsen K. Treating sexually abused children: 1 year follow‐up of a randomized controlled trial. *Child Abuse Neglect*, 2005;29(2):135–145. 10.1016/j.chiabu.2004.12.005	Wrong setting
41. Cohen JA, mannarino PA, Murray LK ^& Igelman R. Psychosocial Interventions for Maltreated and Violence‐Exposed Children. *Journal of Social Issues*, 2006;62(4):737–766. https://doi.org/10.1111/j.1540-4560.2006.00485.x	Wrong setting
42. Cohen, J. A. M., Anthony P., Zhitova, Aren C., Capone, Margery E. (2003). Treating Child Abuse‐Related Posttraumatic Stress and Comorbid Substance Abuse in Adolescents. *Child Abuse & Neglect: The International Journal, 27*(12), 1345–1365. https://doi.org/10.1016/j.chiabu.2003.08.001	Wrong setting
43. Coker AL, Bush HM, Cook‐Craig PG, DeGue SA, Clear ER, Brancato CJ, Fisher BS & Recktenwald EA. RCT testing bystander effectiveness to reduce violence. *American Journal of Preventive Medicine*, 2017;52(5):566–578. https://doi.org/10.1016/j.amepre.2017.01.020	Wrong outcome measures
44. Cook‐Craig PG, Millspaugh PH, Recktenwald EA, Kelly NC, Hegge LM, Coker AL, Pletcher TS. From empower to green dot: Successful strategies and lessons learned in developing comprehensive sexual violence primary prevention programming. *Violence Against Women*, 2014;20(10):1162–1178. https://doi.org/10.1177/1077801214551286	Wrong study design
45. Courtin E, Layte R, Avendano M, Allchin E. Interventions to reduce or prevent exposure to adverse experiences in childhood (ACEs): A systematic review.	Wrong setting
46. Crable AR, Underwood LA, Parks‐Savage A & Maclin V. An Examination of a Gender‐Specific and Trauma‐Informed Training Curriculum: Implications for Provider. *International Journal of Behavioral Consultation & Therapy*, 2013;7(4):30–37.	Wrong setting
47. Crooks CV, Scott K, Ellis W & Wolfe DA. Impact of a universal school‐based violence prevention program on violent delinquency: Distinctive benefits for youth with maltreatment histories. *Child Abuse & Nelgect*, 2011;35(6):393–400. https://doi.org/10.1016/j.chiabu.2011.03.002	Wrong outcome measures
48. Dagenais C & Dutil J. Action in Childcare Settings training programme: Development of an evidence‐based training programme for the prevention of child maltreatment. *Global Health Promotion*, 18;1(66–68).	Wrong study design
49. Dawson G. An evaluation of cognitive and affective outcomes of a prevention program for childhood sexual abuse. EdD Thesis, Memphis State University.	Unable to contact author
50. de Valk s, Beld M, Schaftenaar P & Kuiper C. Does punishment in secure residential youth care work? An overview of the evidence. *Journal of Children's Services*, 2015;10(1):3–16. https://doi.org/10.1108/JCS-11-2014-0048	Wrong study design
51. Decker MR, Wood SN, Ndinda E, Yenokyan G, Sinclair J, Maksud N, Ross B, Omondi B & Ndirangu M. Sexual violence among adolescent girls and young women in malawi: A cluster‐randomized controlled implementation trial of empowerment self‐defense training. *BMC Public Health*, 2018;4(18):1341. https://doi.org/10.1186/s12889-018-6220-0	Wrong setting
52. Deidda M, Boyd KA, Minnis H, Donalson J, Brown K, Boyer NRS & McIntosh E. Protocol for the economic evaluation of a complex intervention to improve the mental health of maltreated infants and children in foster care in the UK (The BeST? services trial). *BMJ*, 2018. https://doi.org/10.1136/bmjopen-2017-020066	Wrong setting
53. DeSena AD, Murphy RA, Douglas‐Palumberi H, Blau G, Blandina K, Horwitz SM & Kaufman J. SAFE Homes: Is it worth the cost? An evaluation of a group home permanency planning program for children who first enter out‐of‐home care. *Child Abuse Neglect*, 29;6(627–643). https://doi.org/10.1016/j.chiabu.2004.05.007	Wrong setting
54. Dhar D, Jain T & Jayachandran S. Reshaping adolescents' gender attitudes: Evidence from a school‐based experiment in India. National Bureau of Economic Research 2018.	Wrong intervention
55. Dhooper SS & Schneider PL. Evaluations of a school‐based child abuse prevention program. Research on Social Work Practice, 1995;5(1):36–46. https://doi.org/10.1177/104973159500500104	Wrong setting
56. Arango DJ, Morton M, Gennari F, Kiplesund S, Ellsberg M. Interventions to prevent or reduce violence against women and girls: A systematic review of reviews. The World Bank 2014	Wrong outcome measures
57. Diaz 2016. *Identifying a history of childhood physical and sexual abuse in adolescents and young adults and understanding its impact on perceived health and health care utilization*. Columbia University, Ann Arbor.	Wrong setting
58. Dorrepaal E, Thomaes K, Smit JH, van Balkom AJLM, van Dyck R, veltman Dj & Draijer N. Stabilizing group treatment for Complex Posttraumatic Stress Disorder related to childhood abuse based on psycho‐education and cognitive behavioral therapy: A pilot study. *Child Abuse & Neglect*, 2010;34(4):284–288. https://doi.org/10.1016/j.chiabu.2009.07.003	Wrong setting
59. Dozier M, Pelooso P, Lindhiem O, Gordon KM, Manni M, Sepulveda S & Ackerman J. Developing evidence‐based interventions for foster children: An example of a randomized clinical trial with infants and toddlers. *Journal of Social Issues*, 2006;62(4):767–785. https://doi.org/10.1111/j.1540-4560.2006.00486.x	Wrong setting
60. Dubas JS, Lynch KB, galano J, Geller S & Hunt D. Preliminary evaluation of a resiliency‐based preschool substance abuse and violence prevention project. *Journal of Drug Education*, 1998;28(3):235–255. https://doi.org/10.2190/VBY0-RLXA-WJ05-NPRX	Wrong setting
61. Dubowitz D, Feigelman S, Lane W & Kim Jeongeun. Pediatric primary care to help prevent child maltreatment: The Safe Environment for Every Kid (SEEK) Model. *Pediatrics*, 2009;123(3):858‐864. https://doi.org/10.1542/peds.2008-1376	Wrong setting
62. Efevbera Y, McCoy DC, Wuermli AJ & Betancourt T. Integrating early child development and violence prevention programs: A systematic review. New Direction for Child and Adolescent Development, 2018;(159):27–54. https://doi.org/10.1002/cad.20230	Wrong setting
63. Ertl V, Pfeiffer A, Schauer E, Elbert T, Neuner F. Community‐implemented trauma therapy for former child soldiers in Northern Uganda: A randomized controlled trial. *JAMA*, 2011;306(5):503–512.	Wrong setting
64. Espelage DL, Valido A, Hatchel T, Ingram KM, Huang Y, Torgal C. A literature review of protective factors associated with homophobic bullying and its consequences among children & adolescents. *Aggression and Violent Behaviour*, 2019;45:98–110. https://doi.org/10.1016/j.avb.2018.07.003	Wrong study design
65. Euser S, Alink LRA, Stoltenborgh M, Bakermans‐Kranenburg MJ, van IJzendoorn MH. A gloomy picture: A meta‐analysis of randomized controlled trials reveals disappointing effectiveness of programs aiming at preventing child maltreatment. *BMC Public Health* 2015;15(1068).	Wrong setting
66. Fantuzzo JW, Stovall A, Schachtel D, Goins C, Hall R. Effects of adult and peer social initiations on the social behavior of withdrawn, maltreated preschool children. Journal of Behaviour Therapy and Experimental Psychiatry, 1987;18(4):357–363. https://doi.org/10.1207/s15374424jccp3402_11	Wrong setting
67. Fantuzzo J, Sutton‐Smith B, Atkins M, Meyers R, Stevenson H, Coolahan K, Weiss A, Manz P. Community‐based resilient peer treatment of withdrawn maltreated preschool children. *Journal of Consulting and Clinical Psychology*, 1996;64(6):1377–1386. https://doi.org/10.1037/0022-006X.64.6.1377	Wrong setting
68. Fantuzzo J, Manz P, Atkins M & Meyers R. Peer‐Mediated Treatment of Socially Withdrawn Maltreated Preschool Children: Cultivating Natural Community Resources. *Journal of Clinical Child Adolescent Psychology*, 2005;34(2):320–335. https://doi.org/10.1037/0022-006X.56.1.34	Wrong setting
69. Farina V, Salemi S, Tatari F, Abdoli N, Jouybai TA, Mostafa A, Basanj B & Zakiei A. Trauma‐focused cognitive behavioral therapy a clinical trial to increase self‐efficacy in abused the primary school children. *Journal of Education and Health promotion*, 2018;1(7):33. https://doi.org/10.4103/jehp.jehp_80_17	Wrong setting
70. Farina V, Naami A, Zargar Y, Davoodi I, Salemi S, Tatari F, Kazemi A, Basanj B, Jouybari TA & Alikhani M. Comparison of trauma‐focused cognitive behavioral therapy and theory of mind: Improvement of posttraumatic growth and emotion regulation strategies. *Journal of Education and Health promotion*, 2018;3(7):58. https://doi.org/10.4103/jehp.jehp_140_17	Wrong setting
71. Farrell AD, Valois RF, Meyer AL. Evaluation of the RIPP‐6 violence prevention program at a rural middle school. *American Journal of Health Education*, 2002;33(3):167–172.	Wrong outcome measures
72. Finkelhor D & Berliner L. Research on the treatment of sexually abused children: A review and recommendations. *Journal of the American Academy of Child and Adolescent Psychiatry*, 1995;34(11):1408–1423.	Wrong setting
73. Fisher PA, Burraston B & Pears K. The Early Intervention Foster Care Program: Permanent Placement Outcomes From a Randomized Trial. Child Maltreatment. *Child Maltreatment*, 2005;10(1):61–71. https://doi.org/10.1177/1077559504271561	Wrong setting
74. Fitzpatrick C. A decreased screen‐violence “media diet” intervention improves preschool children's behavior. *The Journal of Pediatrics*, 2014;164(1):217. https://doi.org/10.1016/j.jpeds.2013.10.058	Wrong outcome measures
75. Foshee VA, Bauman KE, Arriaga XB, helms RW, Koch GG & Linder GF. An evaluation of Safe Dates, an adolescent dating violence prevention program. A*merican Journal of Public Health*, 1998;88(1):45–50.	Wrong setting
76. Foshee VA, Reyes L, Agnew‐Brune CB, Simon TR, Vagi KJ, Lee RD & Suchindran C. The effects of the evidence‐based Safe Dates dating abuse prevention program on other youth violence outcomes. Prevention Science, 2014;15(6):907–916. https://doi.org/10.1007/s11121-014-0472-4	Wrong outcome measures
77. Foshee VA, Benefield T, Dixon KS, Chang LY, Senkomago V, Ennett ST, Moracco KE & Bowling JM. The effects of moms and teens for safe dates: A dating abuse prevention program for adolescents exposed to domestic violence. *Journal of Youth and Adolescence*, 2015;44(5):995–1010. https://doi.org/10.1007/s10964-015-0272-6	Wrong setting
78. Fulu E, Kerr‐Wilson A, Lang J. What works to prevent violence against women and girls? Evidence Review of interventions to prevent violence against women and girls. GOV.UK 2014.	Wrong study design
79. Franziska M, Pantelic M, Spreckelsen TF, Orza L, Little MT, Nittas V, Picker V, Bustmam AA, Romero RH, Mella EPD & Stockl H. Interventions to prevent and reduce gender‐based violence (GBV) among young people living with, or most affected by, HIV in low‐ and middle‐income countries: A systematic review and narrative synthesis. *AIDS*, 2019;15(33):2219–2236.	Wrong setting
80. Gaete J, Valenzuela D, Rojas‐Barahona C,Valenzuela E, Araya R & Salmiavalli C. The KiVa antibullying program in primary schools in Chile, with and without the digital game component: Study protocol for a randomized controlled trial. *Trials*, 2017;18(1):75. https://doi.org/10.1186/s13063-017-1810-1	Wrong outcome measures
81. Garaigordobil M & Martinez‐Valderrey V. Technological Resources to Prevent Cyberbullying During Adolescence: The Cyberprogram 2.0 Program and the Cooperative Cybereduca 2.0 Videogame. *Frontier Psychology*, 2018;9(745). https://doi.org/10.3389/fpsyg.2018.00745	Wrong intervention
82. Grant C. Intimate partner abuse: Young Australians' attitudes and the effectiveness of a brief educational program. Doctoral Dissertation, RMIT University, Melbourne, Australia 2007.	Wrong intervention
Griffith University, 2017 Griffith University. Preventing Youth Sexual Violence and Abuse in Aurukun and West Cairns: Neighbourhoods Project Implementation and Evaluation Report. Queensland: Griffith University.	
83. Griffith University. (2017) *Preventing Youth Sexual Violence and Abuse in Aurukun and West Cairns: Neighbourhoods Project Implementation and Evaluation Report*. Queensland: Griffith University	Wrong study design
84. Gusmoes JDSP, Sanudo A, Valente Y & Sanchez ZM. Violence in Brazilian Schools: Analysis of the Effect of the #Tamojunto Prevention Program for Bullying and Physical Violence. *Journal of Adolescence*, 2018;63:107–117. https://doi.org/10.1016/j.adolescence.2017.12.003	Wrong intervention
85. Habigzang, L. F. S., F. H., Hatzenberger, R., Cunha, R. C., Ramos, Mda, Koller, S. H. (2009). Cognitive behavioral group therapy for sexually abused girls. *Revista de saude publica, 43 Suppl 1*, 70–78.	Wrong setting
86. Hambrick EP, Oppenheim‐Weller S, N'zi AM & Taussig HN. Mental health interventions for children in foster care: A systematic review. *Child and Youth Services Review*, 2016;70:65–77. https://doi.org/10.1016/j.childyouth.2016.09.002	Wrong setting
87. Hanson RK, Bourgon G, Helmus L & Hodgson S. The principles of effective correctional treatment also apply to sexual offenders: A meta‐analysis. *Criminal Justice and Behaviour*, 2009;36(9):865–891.	Child maltreatment type out of scope
88. Hardwick L. Fostering children with sexualised behaviour. *Adoption & Fostering*, 2005;29(2):33–43.	Wrong study design
89. Hatzenbuehler ML, Flores JE, Cazanaugh JE, Onwuachi‐Willig A, Ramirez MR. Anti‐bullying Policies and Disparities in Bullying: A State‐Level Analysis. *American Journal of Preventative Medicine*, 2017;52(2):184–191. https://doi.org/10.1016/j.amepre.2017.02.004	Child maltreatment type out of scope
90. Heidotting T, Keiffer S & Soled SW. A quantitative synthesis of child sexual abuse prevention program. *Child Sexual Abuse Prevention Review*.	Wrong study design
91. Herbert JL & Bromfield L. Evidence for the efficacy of the child advocacy centre model: A systematic review. *Trauma Violence & Abuse*, 2016;17(3):341–357.	Wrong outcome measures
92. Hernandez MC. Exploring the Impact of Multiple Foster Care Placements Among Foster Youth and the Interventions that Address Multiple Placements: A Systematic Literature Review. California State University, Los Angeles, Ann Arbo 2017.	Wrong outcome measures
93. Higareda‐Almaraz MA, Higareda‐Almaraz E, Higareda‐Almaraz IR, Barrera‐de Leon JC, Gomez‐Llamas MA & Benites‐Godinez V. Parental aptitude to prevent child sexual abuse after a participatory education intervention. Salud Publica de Mexico, 2011;53(2):134–140.	Wrong outcome measures
94. Higgins S & Moore T. Keeping our eye on sex, power, relationships, and institutional contexts in preventing institutional child sexual abuse. Child Abuse and Neglect: Forensic Issues in evidence, Impact & Management, 2019;45–62.	Wrong outcome measures
95. Hill J. An evaluation of a school‐based child sexual abuse primary prevention program. *Psychotherapy Bulletin*, 1987;(22):36–38.	Child maltreatment type out of scope
96. Holzaepfel E & Paul D. Evaluating the effectiveness of gender‐based violence prevention programs with refugees in Malaysia. United States Department of State 2013.	Wrong setting
97. IICSA Research Team. Child Sexual Abuse in custodial institutions: A rapid evidence assessment. London: Independent Inquiry into Child Sexual Abuse. 2018.	Wrong setting
98. Iyumade OT. Intervention Models of Non‐formal Education for the Reintegration of Abused Children in South‐Western, Nigeria. 2019.	Wrong setting
99. Jackson V, SHihning C & Browne K. Protective Factors Against Child Victimization in the School and Community: An Exploratory Systematic Review of Longitudinal Predictors and Interacting Variables. *Trauma, Violence & Abuse*, 2017;18(3):303–321. https://doi.org/10.1177/1524838015611675	Wrong study design
100. Jaime MCD, McCauley HL, Tancredi DJ, Nettiksimmons J, Decker MR, Silverman JG, O'Connor B, Stetkevich N & Miller E. Athletic coaches as violence prevention advocates. Journal of Interpersonal Violence, 2014;30(7):1090–1111. https://doi.org/10.1177/0886260514539847	Wrong study design
101. Jankowski KF, Bruce J, Beauchamp KG, Roos LE, Moore WE & Fisher PF. Preliminary evidence of the impact of early childhood maltreatment and a preventive intervention on neural patterns of response inhibition in early adolescence. *Developmental Science*, 2017;20(4). https://doi.org/10.1111/desc.12413	Wrong outcome measures
102. Jaycoz LH, McCaffrey D, Eisman B, Aronoff J, Shelley GA, Collins RL & Marshall GN. Impact of a school‐based dating violence prevention program among Latino teens: Randomized controlled effectiveness trial. *Journal of Adolescent Health*, 2006;39(5):694–704.	Wrong intervention
103. Jemmott JB, O'Leary A, Jemmorr LS, Ngwane ZP, Teitelman AM, Makiwane MB, Bellamy SL. Effect of a behavioral intervention on perpetrating and experiencing forced sex among South African adolescents: A secondary analysis of a cluster randomized trial. *JAMA*, 2018;1(4). https://doi.org/10.1001/jamanetworkopen.2018.1213	Wrong outcome measures
104. Jinich S. *Supportive response training for parents of sexually abused children*. Dissertation Abstracts International: Section B: The Sciences and Engineering, 1995;55(8‐B):3590.	Wrong setting
105. Jones R Ownbey M, Everidge J, Judkins B, & Timbers G. Focused foster care for children with serious sexual behavior problems. *Child and Adolescent Social Work Journal*, 2006;23(3):278–297.	Wrong study design
106. Kajlee L, Zhang L, Langhang L, Munjile K, Tembo S & Menon A, Stanton B, Li X, Malungo J. A randomized‐control trial for the teachers' diploma programme on psychosocial care, support and protection in Zambian government primary schools. *Psychology, Health & Medicine*, 2016;22(4):381–392.	Wrong oucome measures
107. Kane JC, Murray LK, Cohen J, Dorsey S, van Wyk SS, Henderson JG, Imasiku M, Mayeya J & Bolton P. Moderators of treatment response to trauma‐focused cognitive behavioral therapy among youth in Zambia. Journal of Child Psychology and Pyschiatry and allied disciplines 2016;57(10):1194–1202. https://doi.org/10.1111/jcpp.12623	Wrong setting
108. Kaufman K, Erooga M, Stewart K, Zatkin J, McConnell E, Tews H & Higgins D. Risk profiles for institutional child sexual abuse: A literature review. Royal Commission into Insitutional Responses to child Sexual Abuse: Australian Institute of Family Studies 2016.	Wrong outcome measures
109. Keller J, Mboya BO, Sinclair J, Githua OW, Mulinge M, Bergholz L, Paiva L, Golden NH & Kapphahn C. A 6‐week school curriculum improves boys' attitudes and behaviors related to gender‐based violence in Keny. *Journal of Intersponal Violence*, 2017 Feb;32(4):535–557.	Wrong outcome measures
110. Kindt M. *Follow‐up evaluation of a school based sexual abuse prevention program*. Master's Thesis, Bowling Green State Universtity, USA 1991.	Unable to contact author
111. Klevens J & Whitaker DJ. Primary prevention of child physical abuse and neglect: Gaps and promising directions. Child Maltreatment December, 2007;12(4):364–377. https://doi.org/10.1177/1077559507305995	Wrong setting
112. Know Violence in Childhood. Ending violence in childhood global report 2017. Know Violence in Childhood: New Delhi 2017.	Wrong study design
113. Ko SF. Evaluation of school‐based child abuse prevention based on high school follow‐up. Dissertation Abstracts International: Section B: The Sciences and Engineering, 62(8‐B):3828.	Wrong setting
114. Komro KA, Perry CKm Veblen‐Mortenson S, Stigler MH, Bosma LM, Munson KA & Farbaksh K. Violence‐related outcomes of the D.A.R.E. Plus Project. *Health Education & Behaviour*, June 2004;31(3):335–354.	Child maltreatment type out of scope
115. Krahe B & Knappert L. A group‐randomized evaluation of a theatre‐based sexual abuse prevention programme for primary school children in Germany. *Community & Applied Social Psychology*, July 2009;19(4):321–329.	Wrong setting
116. Laing L, Tolliday D, Kelk N & Law B. Recidivism following community based treatment for non‐adjudicated young people with sexually abusive behaviors. *Sexual Abuse in Australian and New Zealand*, 2014;6(1):38–47.	Wrong setting
117. Långström N, Enebrink P, Laurén EM, Lindblom J, Werkö S, & Hanson, R. Preventing sexual abusers of children from reoffending: Systematic review of medical and psychological interventions. *BMJ*, 2013;347.	Wrong setting
118. Lawson J, Maynard B & Berrick J. PROTOCOL: Court Appointed Special Advocates (CASA) as an Intervention for Improving Child Welfare Case Outcomes: A Systematic Review. *Campbell Systematic Reviews*, 2015;11:1‐38.	Wrong outcome measures
119. Lee Y & tang C. Evaluation of a sexual abuse prevention program for female Chinese adolescents with mild mental retardation. *American Journal on Mental Retardation*, 1998;103(2):105–116.	Wrong study design
120. Linares L, Jimenez J, Nesci C, Pearson E, Beller S, Edwards N & Levin‐Rector A. Reducing sibling conflict in maltreated children placed in foster homes. *Prevention Science*, Feb 16 2015;16(2):211–221. https://doi.org/10.1007/s11121-014-0476-0	Wrong setting
121. Liotta L, Springer C, Misurell JR, Block‐Lerner J & Branwein D. Group treatment for child sexual abuse: treatment referral and therapeutic outcomes. *Journal of Child Sexual Abuse*, 30 May 2014;24(3):217–237. https://doi.org/10.1080/10538712.2015.1006747	Wrong setting
122. Livny A & Carmit K. Schools, families and the prevention of child maltreatment: Lessons that can be learned from a literature review. Trauma, Violence & Abuse, 2018;19(2):148–158. https://doi.org/10.1177/1524838016650186	Wrong outcome measures
123. Lynas J & Hawkins R. Fidelity in school‐based child sexual abuse prevention programs: A systematic review. *Child Abuse & Neglect*, 20 July 2017;72:10–21. https://doi.org/10.1016/j.chiabu.2017.07.003	Wrong setting
124. Maclean MJ, Sims S, O'DOnnell M & Gilbert R. Out‐of‐Home Care versus In‐home Care for Children Who Have Been Maltreated: A systematic Review of Health and Wellbeing Outcomes. *Child Abuse Review*, 18 July 2016;25(4):251–272.	Wrong setting
125. Mannarino AP, Cohen JA, Deblinger E, Runyon MK & Steer RA. Trauma‐focused cognitive‐behavioral therapy for children: Sustained impact of treatment 6 and 12 months later. *Child Maltreatment*, August 2012;17(3):231–241. https://doi.org/10.1177/1077559512451787	Wrong setting
126. Matthews B, Walsh K, Coe S, Kenny M & Vagenas D. Child protection training professionals to improve reporting of child abuse and neglect [Protocol]. *Cochrane Database of Systematic Reviews* 2015.	Wrong setting
127. Matthews B, Walsh K, Coe S, Kenny CM & Vagenas D. Protocol for a systematic review: Child protection training for professionals to improve reporting of child abuse and neglect. The Campbell Colloboration 2015.	Wrong outcome measures
128. McCauley HL, Jaime MCD, Tancredi DJ, Silverman JG, Decker MR, Austin SB, Jones K & Miller E. Differences in adolescent relationship abuse perpetration and gender‐inequitable attitudes by sport among male high school athletes. *Journal of Adolecent Health*, June 2014;54(6):742–744. https://doi.org/10.1016/j.jadohealth.2014.01.001	Wrong intervention
129. McKibbin G, Humphreys C & Hamilton B. Prevention‐enhancing interactions: A critical interpretive synthesis of the evidence about children who sexually abuse other children. *Health & Social Care in the Community*, November 2016;24(6):657–671. https://doi.org/10.1111/hsc.12260	Wrong setting
130. Meiskin R, Allen E, Crichton J, Morgan GS, Barter C, Elbourne D, Hunt K, Melendez‐Torres GJ, Morris S, Mc Naughton Reyes HL, Sturgess J, Taylor B, Young H Cmpbell R & Bonell C. Protocol for pilot cluster RCT of project respect: A school‐based intervention to prevent dating and relationship violence and address health inequalities among young people. *BMC*, 2019;5(13). https://doi.org/10.1186/s40814-019-0391-z	Child maltreatment type out of scope
131. Merrill KG, Knight L, Namy S, Allen E, Naker D & Devries KM. Effects of a violence prevention intervention in schools and surrounding communities: Secondary analysis of a cluster randomised‐controlled trial in Uganda. *Child Abuse & Neglect*, August 2014;84:182–195.	Child maltreatment type out of scope
132. Mikton C & Butchart A. Child maltreatment prevention: A systematic review of reviews. World Health Organisation 2019;87:353–361.	Wrong setting
133. Miller E, Goldstein S, mcCauley HL, Jones KA, Dick RN, Jetton J, Silverman JG, Blackburn S, Monasterio E, james L & Tancredi DJ. A school health center intervention for abusive adolescent relationships: A cluster RCT. *Official Journal of the American Academy of Pediatrics*, January 2015;135(1):76–85. https://doi.org/10.1542/peds.2014-2471	Child maltreatment type out of scope
134. Miller E, Das M, Tancredi DJ, McCauley HL, Virata MCD, Nettiksimmons J, O'Connor B, Ghosh S. Evaluation of a gender‐based violence prevention program for student athletes in Mumbai, India. Journal of Interpersonal Violence, 2014;29(4):758–778.	Wrong outcome measures
135. Miller E, Tancredi DJ, Mccauley HL, Decker MR, Virata MCD, Anderson HA, O'Connor B & Silverman JG. One‐year follow‐up of a coach‐delivered dating violence prevention program: A cluster randomized controlled trial. *American Journal of Preventative Medicine*, 2013;45(1):108–112. https://doi.org/10.1016/j.amepre.2013.03.007	Child maltreatment type out of scope
136. Miller E, Tancredi DJ, McCauley HL, Decker MR, Virata MC, Anderson HA, Stetkevich N, Brown EW, Moideen F & Silverman JG. Coaching boys into men: A cluster‐randomized controlled trial of a dating violence prevention program. *Journal of Adolscent Health*, 2012;51(5):431–438. https://doi.org/10.1016/j.jadohealth.2012.01.018	Child maltreatment type out of scope
137. Miller S, Williams J, Cutbush S, Gibbs D, Clinton‐Sherrod M & Jones S. Evaluation of the Start Strong initiative: Preventing teen dating violence and promoting healthy relationships among middle school students. *Journal of Adolescent Health*, 2015;56(2 Suppl 2):S14–9. https://doi.org/10.1016/j.jadohealth.2014.11.003	Child maltreatment type out of scope
138. Moynihan M, Pitcher C & Saewyc E. Interventions that Foster Healing Among Sexually Exploited Children and Adolescents. *Child Sexual Abuse*, 2017;27(4):403–423. https://doi.org/10.1080/10538712.2018.1477220	Wrong setting
139. Murray LK, Skavenski S, Kane JC, Mayeya J, Dorsey S, Cohen JA, Michalopoulos LTM, Imasiky M & Bolton Pa. Effectiveness of Trauma‐Focused Cognitive Behavioral Therapy Among Trauma‐Affected Children in Lusaka, Zambia: A Randomized Clinical Trial. *JAMA Pediatrics*, 2015;169(8):761–769.	Wrong setting
140. Naidoo S, Satorius BK, de vries H & taylor M. Verbal Bullying Changes Among Students Following an Educational Intervention Using the Integrated Model for Behavior Change. *Journal of School Health*, 2016;86(11):816–822. https://doi.org/10.1111/josh.12439	Wrong type of child maltreatment
141. Nelson CA III, Zeanah CH & Fox NA. The effects of early deprivation on brain‐behavioral development: The Bucharest Early Intervention Project. Adolescent Pscyhopathaology and the developing brain: Integrating brain and prevention science 2007;197–215. https://doi.org/10.1093/acprof:oso/9780195306255.003.0009	Book chapter
142. O'Callaghan P, McMullen J, Shannon C, Rafferty H & Black A. A randomized controlled trial of trauma‐focused cognitive behavioral therapy for sexually exploited, war affected Congolese girls. *Journal of the American Academy of Child and Adolescent Psychiatry*, 2013;52(4):359–369.	Child maltreatment type out of scope
143. Ogunfowokan AA & Fajemilehin RB. Impact of a school‐based sexual abuse prevention education program on the knowledge and attitude of high school girls. *Journal of School Nursing*, 2012;28(6):459–468. https://doi.org/10.1177/1059840512446949	Wrong population
144. Pacifici C, Stoolmiller M & Nelson C. Evaluating a prevention program for teenagers on sexual coercion: A differential effectiveness approach. *Journal fo Consulting and Clinical Psychology*, 2001;69(3):552–559.	Child maltreatment type out of scope
145. Parker B & Turner W. Psychoanalytic/Psychodynamic Psychotherapy for Sexually Abused Children and Adolescents: A Systematic Review. *Research on Social Work Practice*, 2014;24(4):389–399. https://doi.org/10.1177/1049731514525477	Wrong setting
146. Parker B & Turner W. Psychoanalytic/psychodynamic psychotherapy for children and adolescents who have been sexually abused. *Cochrane Database of Systematic Reviews*, 2015, Issue 7. https://doi.org/10.1002/14651858.CD008162.pub2	Wrong setting
147. Poche C, Yoder P & Miltenberger R. Teaching self‐protection to children using television techniques. *Journal of Applied Behavior Analysis*, 1988;21(3):253–261.	Child maltreatment type out of scope
148. Quadara A, Higgins D, Nagy V, Lykhina A & Wall L. Therapeutic needs of adult survivors of child sexual abuse: Implications for service provision. Report to the Department of Families, Housing, Community Services and Indigenous Affairs. Melbourne. Department of Families, Housing, Community Services and Indigenous Affairs 2013.	Wrong setting
149. Rheingold A., Campbell C, Self‐Brown S, de Arellano M, Resnick H, & Kilpatrick, D. Prevention of child sexual abuse: Evaluation of a community media campaign. *Child Maltreatment*, 2007;12(4):352–363.	Wrong setting
150. Rheingold A, Zajac K & Palton M. Feasibility and acceptability of a child sexual abuse prevention program for childcare professionals: Comparison of a web‐based and in‐person training. *Journal of Child Sexual Abuse*, 2012;21(4):422–436.	Wrong outcome measures
151. Roche S. Child Protection and Maltreatment in the Philippines: A Systematic Review of the Literature. *Asian and the Pacific Policy Studies*, 2017;4(1):104–128. https://doi.org/10.1002/app5.167	Wrong setting
152. Russell D & Higgins D. Safeguarding capabilities in preventing child sexual abuse: A scale measuring knowledge, attitudes and skills applicable to all youth‐serving sectors. *Child Maltreatment*, 2019;1–43.	Wrong setting
153. Sanders R & McAllen A. Training foster carers of children who have been sexually abused: Issues and dilemma. Child Abuse Review, 1995;4(2):136–145.	Wrong setting
154. Sarnquist C, Kang JL, Amuyunzu‐Nyamongo M, Oguda G, Otieno D, Mboya B, Omondi N, Kipkirui D & Baiocchi M. A protocol for a cluster‐randomized controlled trial testing an empowerment intervention to prevent sexual assault in upper primary school adolescents in the informal settlements of Nairobi, Kenya. *BMC Public Health*, 2019;19(1):834. https://doi.org/10.1186/s12889-019-7154-x	Wrong outcome measures
155. Saunders V & McArthur M. Help‐seeking needs and gaps for preventing child sexual abuse. Royal Commission into Insitutional Responses to Child Sexual Abuse, 2017.	Wrong outcome measures
156. Sayegh Y & Dennis W. The effect of supplementary experiences upon the behavioral development of infants in institutions. *Child Development*, 1965;36:81–90.	Child maltreatment type out of scope
157. Schilling S, Lanier P, Rose R, Shanahan M & Zolotor A. A quasi‐experimental effectiveness study of triple p on child maltreatment. *Journal of Family Violence*, 2019 https://doi.org/10.1007/s10896-019-00043-5	Wrong setting
158. Shetgiri R, Katoaka S, Lin H & Flores G. A randomized, controlled trial of a school‐based intervention to reduce violence and substance use in predominantly Latino high school students. *Journal of the National Medical Association*, 103;9‐10(932–940). https://doi.org/10.1016/S0027-9684%2815%2930450-8	Wrong outcome measures
159. Skar AS, Sherr L, MAcedo A, von Tetzchner S & Fostervold KI. Evaluation of Parenting Interventions to Prevent Violence Against Children in Colombia: A Randomized Controlled Trial. *Journal of Interpersonal Violence*, 2017. https://doi.org/10.1177/0886260517736881	Wrong setting
160. Smyke A, Dumitrescu A & Zeanah CH. Attachment disturbances in young children. I: The continuum of caretaking casualty. *Journal of the American Academy of Child & Adolescent Psychiatry*, 2002;41(8):972–982.	Unable to contact author
161. Spangaro J, Adogy C, Ranmuthugala G, Davies GP, Steinacker L & Zwi A. What is the Evidence of the Impact of Initiatives to Reduce Risk and Incidence of Sexual Violence in Conflict and Post‐conflict Zones and Other Humanitarian Crises in Lower‐and Middle‐income Countries? A systematic review. *PLOS One*, 2013.	Wrong setting
162. Sparling J, Dragomir C, Ramey SL & Florescu L. An educational intervention improves developmental progress of young children in a Romanian orphanage. *Infant Mental Health Journal*, 2005;26(2):127‐142.	Child maltreatment type out of scope
163. Swaim RC & Kelly K. Efficacy of a randomized trial of a community and school‐based anti‐violence media intervention among small‐town middle school youth. *Prevention Science*, 2008;9(3):202–214. https://doi.org/10.1007/s11121-008-0096-7	Wrong intervention
164. Taussig HN & Culhane SE. Impact of a mentoring and skills group program on mental health outcomes for maltreated children in foster care. *Archives of Pediatrics Adolescent Medicine*, 2016;164(8):739–746. https://doi.org/10.1001/archpediatrics.2010.124	Wrong setting
165. Taussig HN, Culhane SE, Garrido E, Knudtson MD, Petrenki CLM. Does severity of physical neglect moderate the impact of an efficacious preventive intervention for maltreated children in foster care? *Child Maltreatment*, 2013;18(1):56–64. https://doi.org/10.1177/1077559512461397	Wrong setting
166. Vandana, C. S., Satapathy, Rajesh, Sagar. (2016). Review of randomized controlled trials on psychological interventions in child sexual abuse: Current status and emerging needs in the Indian context.	Wrong setting
167. Ventevogel P & Spiegel P. Psychological treatments for orphans and vulnerable children affected by traumatic events and chronic adversity in Sub‐Saharan Africa. *JAMA*, 2015;314(5):511–512.	Child maltreatment type out of scope
168. Walker D, McGovern S, Poey E & Otis K. Treatment effectiveness for male adolescent sexual offenders: A meta‐analysis and review. *Journal of Child Sexual Abuse*, 2004;13(3–4):281–293.	Child maltreatment type out of scope
169. WWhitehead H. Hedgehogs pilot programme evaluation report. Brimingham, UK. The Lucy Faithful Foundation.	Wrong study design
170. Yan H, Chen J & Huang J. School Bullying Among Left‐Behind Children: The Efficacy of Art Therapy on Reducing Bullying Victimization. *Frontiers in psychiatry Frontiers Research Foundation*, 2019;10(40). https://doi.org/10.3389/fpsyt.2019.00040	Wrong intervention
171. Yount KM, Krause KH & Miedema SS. Preventing gender‐based violence victimization in adolescent girls in lower‐income countries: Systematic review of reviews. *Social Science & Medicine*, 2017;182:1–13. https://doi.org/10.1016/j.socscimed.2017.08.038	Wrong outcome measures
172. Zeanah CH, Neson CA, Fox NA, Smyke AT, Marshall P, Parker SW & Koga S. Designing research to study the effects of institutionalization on brain and behavioral development: The Bucharest Early Intervention Project. *Development and Psychopathology*, 2003;15(4):885–907. https://doi.org/10.1017/S0954579403000452	Wrong intervention

### Sources of studies

**Table:**

Author	Year	Title	Source
Baker	2012	Increasing knowledge of sexual abuse: A study with elementary school children in Hawaii	Reference list
Baker‐Henningham	2016	Irie Classroom Toolbox: A study protocol for a cluster‐randomised trial of a universal violence prevention programme in Jamaican preschools	Database
Barron	2013	Prevention programs and legal considerations for child sexual abuse cases	Reference list
Bick	2015	Effect of early institutionalization and foster care on long‐term white matter development: A randomised clinical trial	Database
Blumberg	1991	The touch discrimination component of sexual abuse prevention training: Unanticipated positive consequences	Database
Bustamante	2019	“I have the right to feel safe”: Evaluation of a school‐based child sexual abuse prevention programme in Ecuador	Database
Cecen‐Erogul	2013	The effectiveness of psycho‐educational school‐based child sexual abuse prevention training program on Turkish elementary students	Database
Cerezo	2004	Improving child maltreatment detection systems: a largescale case study involving health, social services, and school professionals	Database
Chen	2012	Pilot evaluation of a sexual abuse prevention programme for Taiwanese children	Database
Citak	2017	Preventing child sexual abuse: Body safety training for young children in Turkey	Pundir
Conte	1985	An evaluation of a programme to prevent the sexual victimization of young children	Database
Crowley	1989	Evaluation of good touches/bad touches: A program to prevent child sexual abuse in school‐age children	Reference list
Czerwinski	2018	Effectiveness of a school‐based intervention to prevent child sexual abuse‐evaluation of the German IGEL program	Database
Daigneault	2012	Evaluation of a sexual abuse prevention workshop in a multicultural, impoverished urban area	Reference list
Daigneault	2015	Effectiveness of a sexual assault awareness and prevention workshop for youth: A 3‐month follow‐up pragmatic cluster randomization study	Database
Dake	2003	Evaluation of child abuse prevention curriculum for third‐grade students: assessment of knowledge and efficacy expectations	Database
del Campo Sanchez	2006	Evaluación de un programa de prevención de abusos sexuales a menores en Educación Primaria	Reference list
Devries	2015	The Good School Toolkit for reducing physical violence from school staff to primary school students: a cluster‐randomised controlled trial in Uganda	Websites
Devries	2017	Does the good schools toolkit reduce physical, sexual and emotional violence, and injuries, in girls and boys equally? A cluster‐randomised controlled trial	Database
Devries	2018	Reducing physical violence toward primary school students with disabilities	Database
Dhooper	1995	Evaluation of a school‐based child abuse prevention program	Reference list
Dryden	2014	Effectiveness of the IMPACT: Ability programme to improve safety and self‐advocacy skills in high school students with disabilities	Database
Edwards	2019	Evaluation of a bystander‐focused interpersonal violence prevention program with high school students	Database
Elfreich	2020	Sexual abuse disclosure mediates the effect of an abuse prevention program on substantiation	Reviewer
Feldmann	2018	ReSi: Evaluation of a programme for competency training and prevention of sexual abuse in Kindergarten	Database
Fryer	1987	Measuring actual reduction of risk to child abuse: A new approach	Reference list
Grendel	1991	Cognitive and emotion al effects of a brief child sexual abuse prevention program for first‐graders	Reference list
Gushwa	2019	Advancing child sexual abuse prevention in schools: An exploration of the effectiveness of the enough! Online training program for K‐12 teachers	Database
Harvey	1988	The prevention of sexual abuse: Examination of the effectiveness of a program with kindergarten‐age children	Reference list
Hazzard	1991	Child sexual abuse prevention: Evaluation and one‐year follow‐up	Reference list
Heidotting	1994	A quantitative synthesis of child sexual abuse prevention programs	Database
Herbert	2001	Proximate effects of a child sexual abuse prevention program in elementary school children	Reference list
Hermenau	2017	Fostering child development by improving care quality: A systematic review of the effectiveness of structural interventions and caregiver trainings in institutional care	Database
Hillenbrand‐Gunn	2012	Men as allies: The efficacy of a high school rape prevention intervention	Reference list
Humphreys	2015	Effects of institutional rearing and foster care on psychopathology at age 12 years in Romania: Follow‐up of an open, randomised controlled trial	Database
Jin	2017	Evaluation of a sexual abuse prevention education programme for school‐age children in China: a comparison of teachers and parents as instructors	Database
Johnson	2010	Growth and associations between auxology, caregiving environment, and cognition in socially deprived Romanian children randomised to foster vs ongoing institutional care	Database
Kenny	2012	Evaluation of a personal safety program with Latino preschoolers	Reference list
Kolko	1987	Promoting awareness and prevention of child sexual victimization using the Red Flag/Green Flag program: An evaluation with follow‐up	Reference list
Kolko	1989	Classroom training in sexual victimization awareness and prevention skills: An extension of the Red Flag/Green Flag people program	Reference list
Krahe	2009	A group‐randomized evaluation of a theatre‐based sexual abuse prevention programme for primary school children in Germany	Reference list
Knight	2018	Implementation of the Good School Toolkit in Uganda: a quantitative process evaluation of a successful violence prevention program	Database
Kraizer	1988	Programming for preventing sexual abuse and abduction: What does it mean when it works?	Database
Kraizer	1991	The Safe Child Program for the prevention of child abuse: Development and evaluation of a school‐based curriculum	Reference list
McElearney	2018	Cluster randomised controlled trial of “whole school” child maltreatment prevention programme in primary schools in Northern Ireland: study protocol for Keeping Safe	Database
McIntyre	1999a	Evaluation of the effectiveness of the Stay Safe primary prevention programme for child abuse	Reference list
McIntyre	1999b	Helping children to the other side of silence: A study of the impact of the Stay Safe Programme on Irish children's disclosures of sexual victimisation.	Reviewer
McKibbin	2017	Preventing harmful sexual behaviour and child sexual exploitation for children & young people living in residential care: A scoping review in the Australian context	Database
Merrill	2018	Effects of a violence prevention intervention in schools and surrounding communities: Secondary analysis of a cluster randomised‐controlled trial in Uganda	Pundir
Neherta	2017	The difference in intervention of sexual abuse prevention by two variance professions on primary school children in Padang	Database
Nkuba	2018	Reducing violence by teachers using the preventative intervention Interaction Competencies with Children for Teachers (ICC‐T): A cluster randomized controlled trial at public secondary schools in Tanzania	Database
Oldfield	1996	Evaluation of the effectiveness of Project Trust: An elementary school‐based Victimization Prevention Strategy	Reference list
Pitts	2015	Child sexual abuse prevention programmes for pre‐schoolers: A synthesis of current evidence	Inquiries‐Aus
Pulido	2015	Knowledge gains following a child sexual abuse prevention programme among urban students: A cluster‐randomized evaluation	Database
Quadara	2015	Conceptualising the prevention of child sexual abuse. Final Report	Expert Input
Radford	2017	Rapid evidence assessment: What can be learnt from other jurisdictions about preventing and responding to child sexual abuse	Inquiries‐Ind
Ratto	1990	An evaluation of a preschool curriculum to educate children in the prevention of sexual abuse	Database
Rheingold	2014	Child sexual abuse prevention training for childcare professionals: An independent multi‐site randomized controlled trial of stewards of children	Expert Input
Ricardo	2011	Engaging boys and young men in the prevention of sexual violence: A systematic and global review of evaluated interventions	Pundir
Saslawsky	1985	Educating children about sexual abuse: Implications for pediatric intervention and possible prevention	Reference list
Sherr	2017	Child violence experiences in institutionalised/orphanage care	Database
Smyke	2010	Placement in foster care enhances quality of attachment among young institutionalized children	Database
Snyder	1986	An evaluation of the 'good secrets, bad secrets' sexual assault prevention program	Reference list
South	2015	Scoping review: Evaluations of out‐of‐home care practice elements that aim to prevent child sexual abuse	Inquiries‐Aus
Ssenyonga	2018	Reducing violence against children by implementing the preventative intervention Interaction Competencies with Children for Teachers (ICC‐T): Study protocol for a cluster randomized controlled trial in Southwestern Uganda	Database
Sullivan	1992	The effects of psychotherapy on behavior problems of sexually abused deaf children	Database
Taal	1997	Positive and negative effects of a child sexual abuse prevention program	Reference list
Taylor	2010	The effects of gender violence/harassment prevention programming in middle schools: A randomized experimental evaluation	Database
Telljohann	1997	Evaluation of a third grade sexual abuse curriculum	Reference list
Topping	2009	School‐based child sexual abuse prevention programs: A review of effectiveness	Reference list
Troller‐Renfree	2015	The effects of early foster care intervention on attention biases in previously institutionalized children in Romania	Database
Tutty	1997	Child sexual abuse prevention programmes: Evaluating Who Do You Tell	Database
vanLieshout	2019	Make a move: A comprehensive effect evaluation of a sexual harassment prevention program in Dutch Residential Youth Care	Database
Wade	2019	Long‐term effects of institutional rearing, foster care, and brain activity on memory and executive functioning	Database
Walsh	2015	School‐based education programmed for the prevention of child sexual abuse (Review)	Campbell
Warden	1997	An evaluation of a children's safety training program	Reference list
Weatherley	2012	Evaluation of a school‐based sexual abuse prevention curriculum in Malaysia	Pundir
White	2018	Promoting young children's interpersonal safety knowledge, intentions, confidence, and protective behavior skills: Outcomes of a randomized controlled trial	Expert Input
Wolfe	1986	Evaluation of a brief intervention for educating school children in awareness of physical and sexual abuse	Reference list
Wurtele	1986	Teaching personal safety skills for potential prevention of sexual abuse: A comparison of treatments.	Reference list
Wurtele, Gillispie	1992	A comparison of teachers vs parents as instructors of a personal safety program for preschoolers	Reference list
Wurtele, Kast	1992	Sexual abuse prevention education for young children: A comparison of teachers and parents as instructors	Inquiries‐Aus
Zhang	2014	Evaluation of a sexual abuse prevention education for Chinese preschoolers	Database
Zwi	2007	School‐based education programmes for the prevention of child sexual abuse	Campbell

### Characteristics of primary studies

**Table:**

Study	Design (status)	Country	Maltreatment type/s	Setting	Child target population (age)	Intervention delivery personnel/approach	Alignment with WHO‐INSPIRE	Intervention category and primary focus	Brief description of the intervention components	
Baker et al. (2012)	QED	USA	Sexual abuse	Primary/elementary school	Children from grades 3–5 (7–10 years old)	Train the trainer approach: school staff including teachers, counselors, social workers, and human resource professionals given an overview of relevant information about child abuse and how to teach sensitively and deal with abuse disclosure	Education and life skills	Prevention: To increase children's knowledge of sexual abuse and develop self‐protection/safety skills	MBMB curriculum: (My Body My Boundries), consists of topics to help children identify types of sexual abuse, recognise and respond to unsafe and unwanted behaviours, learn how to identify and tell others if they have been abused, understanding personal boundaries and learning to stay safe from internet predators	
Baker‐Henningham et al. (2016)	RCT (ongoing)	Jamaica	Physical abuse	Preschool	Children in preschool (3–5 years old)	Trained classroom teachers	Education and life skills	Prevention: To improve teacher classroom behaviour management and teach strategies in order to decrease the use of violence and promote children's social‐emotional competence	The Irie Classroom Toolbox: Training for teachers delivered through five full‐day workshops, monthly in‐class coaching over two school terms, and weekly text messages, provision of resources (guidance booklet, lesson plans, games, song books, picture cards)	
Barron and Topping (2013)	RCT	UK	Sexual abuse, physical abuse, emotional abuse	Secondary/high school	Children from grades 6–8 (11–13 years old)	Presenters who have received a 1‐h training delivered by the program authors in program aims/objectives, content, delivery style, confidentiality limits, and response to disclosure	Education and life skills	Prevention: To increase children's knowledge and identification of safe and risky people and situations, and knowledge/skills about how to disclose abuse	The Tweenees program (Matthew & Laurie, 2002) has two main aims: to enable students to be aware of potentially abusive situations and to disclose CSA and other abuses. Lessons are 50 min long	
Blumberg (1991)	RCT	USA	Sexual abuse	Primary/elementary school	Children from kindergarten to grade 3 (5–9 years old)	Trained volunteers social workers	Education and life skills	Prevention: To increase awareness and understanding of child sexual abuse and to teach self‐protection/safety skills	Role play modeling, rehearsal and discussion, presentations. Toys such as teddy bears, puppets were used to demonstrate and initiate discussion. A film was also shown to learn about self‐protection, sexual abuse and strangers	
						Presentations were also delivered by educators, counselor, school nurse and teachers			
Bustamante (2019)	RCT	Ecuador	Sexual abuse	Primary/elementary school	Children in primary/elementary school (6–14 years old)	“Train the trainer”—Trained psychologist guided schoolteachers on how to conduct the program. Teacher then had one week to prepare before leading the weekly activity in his/her class	Education and life skills	Prevention: To increase children's knowledge of child sexual abuse and self‐protection strategies	Specific activities for each session were organised in a workbook given to each child and teacher.	
									Sessions involved interactive activities, and discussion of the lessons from the activity through a set of “powerful questions”
Cecen‐Erogul and Hasirci (2013)	RCT	Turkey	Sexual abuse	Primary/elementary school	Children in grade 4 (9‐–10 years old)	Not reported	Education and life skills	Prevention: To increase children's knowledge of child sexual abuse and self‐protection strategies	One‐hour session, on four consecutive days.	
									Videos and lecture, role‐playing, modeling, and rehearsal teaching techniques
Cerezo and Pons‐Salvador (2004)	QED	Spain	Neglect; physical abuse; sexual abuse; emotional abuse	Multiple (including health services, social services agencies, schools)	Phase 1: All children under 18 years old.	Not reported	Response and support services	Prevention and Response: To improve rates of child maltreatment detection and reporting	Professionals were trained to better detect signs of child maltreatment and how to follow a protocol about reporting suspected child maltreatment cases to child protection	
					Phase 2: Children in preschool and primary/elementary school (3–10 years old)				
Chen et al. (2012)	RCT	Taiwan	Sexual abuse	Primary/elementary school	Children in primary/elementary school (6–13 years old)	Not reported	Education and life skills	Prevention: To increase children's knowledge of child sexual abuse and self‐protection strategies	Skills‐based child sexual abuse prevention program	
									(a) sexual abuse knowledge and prevention and (b) abduction prevention. included the following: (a) body ownership, (b) distinguishing appropriate from inappropriate touches or requests to touch and the responsibility associated with inappropriate touching, and (c) distinguishing types of secrets
Citak Tunc et al. (2018)	RCT	Turkey	Sexual abuse	Preschool	Children in preschool (3–5 years old)	Not reported	Education and life skills	Prevention: To teach preschoolers about body safety and self‐protection strategies	Body Safety Training Program :10 sessions, each session lasts between 20 and 25 min and is implemented in small groups (6–10 children)	
									In each session, the children participated in various (interactive and experiential) exercises and exchange views about the topics, e.g., they discuss situations in which touches or kisses from adults feel like a boundary violation and how to deal with such situations. The first five sessions cover “general safety” topics and the remaining five sessions focus on “body safety”
Conte (1985)	RCT	USA	Sexual abuse	All day and after school program	Children in an all day and after school program (4–10 years old)	Deputy sheriffs who have gone through general training on the problem of sexual abuse of children and have been trained specifically in sexual abuse prevention	Education and life skills	Prevention: To increase children's knowledge of child sexual abuse and self‐protection strategies	The programme is presented on three consecutive days for 1 h each day. The programme teaches children personal safety awareness, assertiveness training, and practical self‐protection skills (e.g., where it is safe to walk in the city). Children are also taught to assertively say no when touched in not‐OK ways and to say no, run, and tell as a basic protective response to threatening situations. Through role plays, children practice ways of saying no to adults who may touch them inappropriately	
Crowley (1989)	RCT	USA	Sexual abuse	Primary/elementary school	Children from grades 4–5 (Age not reported)	Certifies school psychologists, social workers, and school nurse/teacher who received at least two training sessions from the program developers	Education and life skills	Prevention: To increase children's knowledge of child sexual abuse and self‐protection strategies	Good Touches/Bad Touches (GT/BT) program: Uses a structured lesson format combined with activities for the children, a film, and extensive discussion sessions. The program was designed to be presented to children from kindergarten through grade six and is conscious of the developmental level of the students. The GT/BT program teaches a core of concepts present in other sexual abuse programs	
Czerwinski et al. (2018)	QED	Germany	Sexual abuse	Primary/elementary school	Children in grade 3 (8–9 years old)	The programme is implemented by teachers who have undergone training in two workshops and received all the necessary materials for its implementation	Education and life skills	Prevention: To increase children's knowledge of child sexual abuse and self‐protection strategies. In addition, the programme aims to raise the school personnel's awareness of sexual abuse and enable them to identify inappropriate situations and react adequately	The programme comprises seven school sessions. In each session, the children participate in various (interactive and experiential) exercises and exchange views about the topics, e.g., they discuss situations in which touches or kisses from adults feel like a boundary violation and how to deal with such situations. Involves two‐day training workshop for teachers. materials and implementation support	
Daigenault (2012)	RCT	Canada	Sexual abuse; physical abuse	Primary/elementary school	Children from grades 1–4 (5–11 years old)	Not reported	Education and life skills	Prevention: To increase children's knowledge of child sexual abuse and self‐protection strategies	ESPACE consists of a 90 min workshop delivered by 3 community workers. Activities include role‐playing, guided discussions, behaviour modelling to enhance, promote and teach children awareness of personal right, self‐assertions skills and responses to abuse. Booster sessions included a revision of the ESPACE workshop	
Daigneault et al. (2015)	RCT	Canada	Sexual abuse	Secondary/high school	Youth in secondary/high school (15–17 years old)	Facilitated by two female sexual assault advocacy centre staff	Education and life skills	Prevention: To improve knowledge of, response and disclosure of sexual assault	One 75‐min workshop delivered to one classroom at a time	
Dake et al. (2003)	RCT	USA	Physical abuse, sexual abuse, emotional abuse, neglect	Primary/elementary schools	Children in grade 3 (8–9 years old)	Delivered by child abuse prevention agency employees and trained volunteers	Education and life skills	Prevention: To increase children's knowledge of child abuse and self‐protection strategies	Two 1‐h sessions. Curriculum included class discussions of abuse concepts and teacher roleplays depicting abusive scenarios to increase children's knowledge of child abuse and confidence to respond	
del Campo Sánchez and Sánchez (2006)	RCT	Spain	Sexual abuse	Primary/elementary schools	Children from grades 3–6 (8–12 years old)	Not reported (before the intervention, a training session with teachers and parents was conducted to offer knowledge and prepare them to interact with the children during and after the intervention)	Education and life skills	Prevention: To increase children's knowledge of child abuse and self‐protection strategies	The intervention consisted of two 1‐h sessions during school hours	
Dhooper & Schneider (1995)	QED	USA	Physical abuse; sexual abuse	Primary/elementary schools	Children from grades 3–5 (Age not reported)	Not reported	Education and life skills	Prevention: To enhance children's understanding of child abuse and their ability to recognise abuse and to interrupt and/or avoid abusive situations	This school‐based educational program provided by the Family Nurturing Center of Kentucky (an agency specializing in child abuse issues and responsible for offering prevention programs in schools) is based on the belief that complete learning requires not only the thinking process but internalizing concepts on a feeling level as well. It uses the KIDS ON THE BLOCKTM puppets and skits. Puppets serve as models with which children can identify on both affective and cognitive levels	
Dryden (2014)	QED	USA	Physical abuse; sexual abuse	Secondary/high school	Youth with disabilities in secondary/high school (13–21 years old)	Teaching team includes one instructor who coaches students through these scenarios and a second instructor who plays the role of an unsafe, untrustworthy, or challenging individual	Education and life skills	Prevention: To increase safety and self‐advocacy skills of children with disabilites	IMPACT:Ability: Consists of ten 90‐min weekly class sessions. Eight classes focus on training methods that place students in realistic simulations of unsafe and uncomfortable situations ranging from refusing unwanted help from a stranger to attempted sexual abuse and abduction. Two classes discuss the meaning of self‐determination and self‐advocacy with a facilitator, where participants engage in creative activities around future goals, develop an action plan toward one goal, and identify people that can help them reach that goal	
Edwards et al. (2019)	RCT	USA	Physical abuse; sexual abuse.	Secondary/high school	Children in grades 9–12 (14–18 years old)	Vast majority of sessions co facilitated by one facilitator who identifies as male and one facilitator who identifies as female	Norms and values	Prevention: To teach students how to safely and effectively intervene before, during, and after situations of relationship abuse and sexual assault to both prevent and stop these forms of abuse from happening, as well as supporting victims in the aftermath of these experiences	Bringing in The Bystander (BITB): A seven‐session curriculum delivered to a mixed sex audience in class periods (approximately 45 min per session) and include lectures, large and small group discussions, hands‐on and experiential exercises, skill‐building activities, and video segments.	
									In addition to student programming, the BITB‐HSC includes a 60‐ min School Personnel Workshop that trains teachers and other school staff skills to be positive bystanders in situations of adolescent interpersonal violence
Elfreich (2020)	QED	USA	Physical abuse; sexual abuse	Primary/elementary school	Children in primary/elementary school (disclosure data reported here was collected between 0 and 9 years after participation in the program)	Not reported	Education and life skills	Prevention: To teach children how to avoid abusive situations (sexual and physical), while also empowering children who have experienced abuse to report to a trusted adult	Think First, Stay Safe: A multiple session program delivered in classrooms. The program is altered in ways that are developmentally appropriate for children. For instance, for kindergartners and first graders, the information is presented in a shorter format and mature, sexual abuse‐related language is not yet introduced. For children in third grade through sixth grade, the curriculum becomes a little longer, includes peer role‐playing, information about common lures used by perpetrators, and more sexual abuse‐relevant language	
Feldmann et al. (2018)	RCT	Germany	Sexual abuse	Kindergarten; daycare	Children in Kindergarten (3–6 years old)	Taught to children by early childhood educators. Educators receive a 1‐day pre‐service training and then administer the programme on their own without further implementation support	Education and life skills	Prevention: To increase children's knowledge of child sexual abuse and self‐protection strategies	The ReSi program: The programme is structured into four competency domains: Emotions, Body, Relations, and Language. Teaching is based on “exercises” and “play”. Exercises are structured into obligatory “core” exercises and optional exercises. Each exercise is described in a manual, and educators use dolls, picture books, an emotion rubric and decks of cards to support the exercises	
Grendel (1991)	RCT	USA	Sexual abuse	Primary/elementary schools	Children in grade 1 (6–8 years old)	Classroom teachers and special services personnel are trained to present the program, to recognise and respond to children who are currently being abused and to report the abuse properly. Parents are educated through a seminar and supplementary materials the children bring home daily	Education and life skills	Prevention: To increase children's knowledge of child sexual abuse and self‐protection strategies	Women Helping Women Prevention Program: The program for first graders consisted of a single, 50‐min presentation to individual classes conducted by Women Helping Women's Education Coordinator. In the first 15 min, the presenter introduced the concepts of body safety rules. Following the presentation, children viewed a 20 min film that uses a vicarious presentation of a group of children participatin in a prevention program as the means to educate the children watching the film. The teacher in the film plays a game called “Let's pretend”, in which children picture themselve in four potentially abusive situations. The film stresses the four body safety rules that are emphasized in the initial presentation	
Gushwa (2018)	RCT	USA	Sexual abuse	K‐12 school	Children from kindergarten to year 12 (5–18 years old)	N/A	Education and life skills	Prevention: To increase knowledge of behaviors associated with grooming and awareness of the importance of reporting responsibilities	Enough! Preventing Child Sexual Abuse in My School: An online training course for school employees including teachers, administrators, counselors, coaches, office personnel, and support staff. Training is available in a 1‐h interactive course that learners can choose to take in one session or in separate 20‐min segments. The training addresses signs and symptoms of CSA, grooming, and sexual misconduct behaviors, reporting responsibilities and requirements (including consequences for failure to report), with the focus on debunking some of the misconceptions and fears/biases associated with responding to and reporting suspected abuse/misconduct	
Harvey (1988)	RCT	USA	Sexual abuse	Primary/elementary school	Children in kindergarten (5–6 years old)	Two college‐educated experimenters with substantial experience working with children delivered the program	Education and life skills	Prevention: To increase children's knowledge of child sexual abuse and self‐protection strategies	Good Touch‐Bad Touch: Three 1/2‐h sessions occurring across three consecutive days with approximately 20 children in each session. The program consists of the following components: Defining sexual abuse; differentiating between good (e.g., holding hands with another child), bad (e.g., hitting and kicking others), and sexually abusive touches; delineating safety rules to prevent abuse; and identifying who can sexually abuse children (e.g., a stranger, a familiar adult, or teenager). Instructions, modeling, rehearsal, and social reinforcement were utilised as teaching procedures. In the first session the concepts and skills were taught by instruction, through a story and large storybook, and by playing a simple game. The second session consisted of reviewing the material covered in the first session, observing a film designed to prevent sexual abuse, and learning a simple song about body safety. The third session involved reviewing material covered in session two, reading a story about who sexually abuses children, and finally presenting two stories in which the concept that victims of sexual abuse are not bad is taught	
Hazzard et al. (1991)	RCT	USA	Sexual abuse	Primary/elementary school	Children in grades 3 and 4 (age not reported)	Not reported	Education and life skills	Prevention: To increase children's knowledge of child sexual abuse and self‐protection strategies	Feeling Yes, Feeling No: The program was a three‐session format. Each session was an hour long and led by a female mental health professional with expertise in child sexual abuse. Each session included a 15‐min videotape, group discussions, and role‐plays. In each classroom, a prevention poster was display and a silent question box which a child could anonymously ask a question	
Herbert (2001)	QED	Canada	Sexual abuse	Primary/elementary school	Children in grades 1 and 3 (6–9 years old)	Led by specialized community workers	Education and life skills	Prevention: To increase children's knowledge of child sexual abuse and self‐protection strategies	The ESPACE program consists of a 60–75 min workshop, and uses roleplaying, guided discussions, behavior modeling, and rehearsal. Children are taught self‐assertion skills, a self‐defense yell, and are encouraged to ask friends for help and to tell a trusted adult if an incident of abuse occurs	
Hillenbrand‐Gunn (2012)	QED	USA	Sexual abuse	Secondary/high school	Youth from grades 10–12 (16–18 years old)	Delivered by a sexual violence prevention expert	Education and life skills	Prevention: The program aims to teach high school students about rape‐supportive behaviour and promote positive changes in their behaviours and attitudes	The Men As Allies intervention consists of the following: (1) participants read and discuss acts of courage challenging sexist, coercive, and abusive behavior or attitudes; (2) music video sung by a male rap artist about men's role in preventing sexual violence, which focuses on men who stand up against violence against girls and women; (3) music video sung by a male rap artist about men's role in preventing sexual violence, which focuses on men who stand up against violence against girls and wome; (4) emphasizing the key role of males in helping females who have been raped, including how affirming support from a male friend following a rape positively influences the rape survivors' recovery process; (5) incorporating a male role model, a respected coach, or a crisis counselor who introduces the presenter prior to the first session	
Jin et al. (2017)	RCT	China	Sexual abuse	Primary/elementary school	Children in primary/elementary school (6–12 years old)	Delivered either by parents or teachers	Education and life skills	Prevention: To increase children's knowledge of child sexual abuse and self‐protection strategies	The curriculum consists of the following: (i) the concept of private parts; (ii) body safety rules; (iii) recognition of appropriate/inappropriate touch; (iv) strategies for saying “no” in life situations, and (v) self‐protection skills. For a teacher education group, the curriculum was administered to children by trained teachers and finished in three 30‐min sessions. For a parent education group, the exact same curriculum contents as taught in the teacher education group were compiled into a handbook, so that parents could read and tutor their children	
Kenny et al. (2012)	QED	USA	Sexual abuse	Preschool; daycare	Children in preschool or daycare (3–5 years old)	Not reported	Education and life skills	Prevention: To teach preschoolers and their parents knowledge of child sexual abuse and self‐protection strategies to prevent child sexual abuse	Kids Learning about Safety (KLAS) program consists of 10 h of psychoeducation focused on teaching preschoolers and their parents safety skills. The program encompassed the Body Safety Training workbook and materials from Talking About Touching curriculum. Both are personal safety curriculums that teach children simple rules	
Kolko (1987)	QED	USA	Sexual abuse	Primary/elementary school	Children in grades 3 and 4 (7–11 years old)	Adult staff volunteers recruited from the community and teachers from each classroom were exposed to an extensive in‐service training program conducted by caseworkers from Children and Youth Services which involv‐ed didactic instruction, roleplaying, and group discussion	Education and life skills	Prevention: To increase children's knowledge of child sexual abuse and self‐protection strategies, including strategies for disclosure	Red Flag/Green Flag: The main content of the curriculum is outlined in RF/GF People Coloring Book. The book includes common situations depicting the various types of physical and non‐physical methods with which children and adults communicate. Its content serves as a vehicle for eliciting child reports of problematic or traumatic experiences, sensitizing children to the need to report potentially abusive situations to an adult, and teaching preventive skills. Specific behavioral strategies highlighted in the book include (1) how to say no to an adult, (2) how to get away from a perpetrator, and (3) how to tell someone about the experience of an actual abusive incident. A film reiterates these concepts	
Kolko (1989)	QED	USA	Sexual abuse	Primary/elementary school	Children primary/elementary school (7–10 years old)	Fourteen staff volunteers were recruited from the community or school to discuss the program materials in each classroom and received a formal in‐service training program	Education and life skills	Prevention: To increase children's knowledge of child sexual abuse and self‐protection strategies, including strategies for disclosure	Red Flag/Green Flag: The main content of the curriculum is outlined in RF/GF People Coloring Book. The book includes common situations depicting the various types of physical and non‐physical methods with which children and adults communicate. Its content serves as a vehicle for eliciting child reports of problematic or traumatic experiences, sensitizing children to the need to report potentially abusive situations to an adult, and teaching preventive skills. Specific behavioral strategies highlighted in the book include (1) how to say no to an adult, (2) how to get away from a perpetrator, and (3) how to tell someone about the experience of an actual abusive incident. A film reiterates these concepts	
Krahe (2009)	RCT	Germany	Sexual abuse	Primary/elementary school	Children in grades 1 and 2 (6–9 years old)	The intervention package includes a 3‐h training session for teachers to prepare them for their task of guiding the children through the performance (the intervention) and a 3‐h information evening for parents designed to provide facts and raise awareness about sexual abuse	Education and life skills	Prevention: To increase children's knowledge of child sexual abuse and self‐protection strategies	Children watched a live performance of the play “(No) Child's Play”, or watched a DVD version of the live performance of the play. (No) Child's Play (Klein Kinderspiel), was developed by a team of experts (psychologists, teachers, theatre education professionals and police officers) for first and second grade primary school children. Lasting for about 60 min, it was designed to promote children's skills in handling interactions with adults in which they feel uncomfortable, such as being asked to keep a secret about which they feel uneasy, and in promoting confidence in their ability to seek help	
Krazier (1991)	QED	USA	Sexual abuse; physical abuse; emotional abuse	Preschool; Primary/elementary school	Children from preschool to grade 3 (age not reported)	Trained teachers	Education and life skills	Prevention: To increase children's knowledge of child sexual abuse and self‐protection strategies	The Safe Child Program runs for 5–10 by trained teachers. Material is presented through video tapes and activities. The video teaches the children the concept around sexual abuse and self‐protection, including key skills and words which help protect them. Role‐playing is used by the classroom teacher to turn the concepts into skills for each child. As the children practice the techniques, they develop mastery	
MacIntyre (1999a)	QED	UK	Sexual abuse	Primary/elementary school	Children in grades 2 and 5 (7 and 10 years old)	Teachers	Education and life skills	Prevention: To increase children's knowledge of child sexual abuse and self‐protection strategies	The Stay Safe program utilises a multimedia format and focuses on cognitive, affective and behavioural dimensions of learning by teaching strategies for dealing with bullying and child sexual abuse. The child's training was conducted over 12 sessions for 7 year olds and 10 sessions for 10 year olds. Sessions were of 30–40 min duration and included structured lesson plans and written, video, and audio teaching materials	
MacIntyre (1999b)	QED	UK	Sexual abuse	Primary/elementary school	Children in primary/elementary schoo (disclosure data reported here is after the referral of children for suspected child sexual abuse; mean age = 8 years old at the time of this study)	Teachers	Education and life skills	Prevention: To increase children's knowledge of bullying and child abuse, as well as self‐protection strategies	Stay Safe Program: Is delivered across five modules that cover feeling safe/unsafe, touches, bullying, strangers, and secrets and telling. Through classroom discussion, roleplay and repetition, children learn simple safety strategies for dealing with problems. Children learn that they should always tell an adult who can help. The overall message is that children will learn to: Say “No”, get away and tell	
McElearney et al. (2018)	RCT (ongoing)	UK	Neglect; sexual abuse	Primary/elementary school	Children in primary/elementary school (4–11 years old)	Teachers and school staff	Education and life skills	Prevention: To teach children how to keep safe from from all forms of maltreatment, including: neglect, sexual abuse carried out online or using digital technology, abuse perpetrated by other children, and bullying	Keeping Safe: A multicomponent “whole school” approach, premised on three core themes, which are healthy relationships, my body, and being safe. The program uses classroom‐based materials including 63 lesson plans (nine for each grade per year). School leaders will deliver a prepared assembly, one of 12 available, to introduce the theme for the term. Each teacher will then deliver three age appropriate lessons to their class and ask the children to complete the accompanying homework with their parents or carers. Parents will be engaged in directed homework activities with their children and are encouraged to attend a structured information session and expert workshops. Training and support for teachers and whole school staff provided in a blended package of training aimed at building the capacity of school leaders, teaching and non‐teaching staff to teach and embed the program	
Neherta et al. (2017)	QED	Indonesia	Sexual abuse	Primary/elementary school	Children in primary/elementary school (6–12 years old)	Teachers	Education and life skills	Prevention: To increase children's knowledge of child sexual abuse and self‐protection strategies	Interventions given to children were knowledge increasing programs for the prevention of sexual abuse using VAK learning modalities (Visual Auditory Kinesthetic). The intervention used a variety of learning media, such as movies, presentation, role play, discussion, a pictorial sketch story, local language song, and leaflets	
Nkuba et al. (2018)	RCT	Tanzania	Physical abuse	Secondary/high school	Children in secondary/high school (13–17 years old)	Teachers. To implement the ICC‐T intervention in the selected schools, one Tanzanian psychologist conducted the ICC‐T training workshop with the help of three assistant facilitators	Education and life skills	Prevention: Aims at preventing violent discipline and improving teacher–student relationships by introducing essential interaction competencies into the daily work of teachers	Interaction Competencies with Children for Teachers program. Intervention components included sessions on: teacher‐student interaction; child maltreatment; effective discipline strategies; identifying and supporting burdened students, and the implementation of ICC‐T components in everyday school life	
Oldfield et al. (1996)	RCT	USA	Sexual Abuse	Primary/elementary school	Children from grades 1–6 (age not reported)	High school students perform a play for primary school students, followed by a question‐response session	Education and life skills	Prevention: To increase children's knowledge of child sexual abuse and self‐protection strategies	Project TRUST: trained high school students performed a play for primary school aged‐students, lasting 30 min followed by a 15 min question‐response period. Topics included were touch continuum, right to question and refuse touch, how to say no and perpetrators can be either people you know or strangers	
Pulido (2015)	RCT	USA	Sexual abuse	Primary/elementary school	Children in grades 2 and 3 (7–9 years old)	Two master's‐level clinical social workers or mental health counselors facilitated each workshop	Education and life skills	Prevention: To increase children's knowledge of child sexual abuse and self‐protection strategies	Safe Touches: classroom‐based 50‐min interactive workshop in which racially ambiguous puppets are used to role‐play scenarios that help children learn and practice safety concepts. Children are also given an age appropriate activity book on body safety to complete at home with caregivers. Facilitators guide the children in making a list of what to do if they experience a not‐safe touch and whom to tell, as well as in practicing the assertive language skills needed to express discomfort and to talk with a trusted adult about a not‐safe touch	
Ratto (1990)	RCT	USA	Sexual abuse	Daycare	Children in daycare (37–62 months old)	Not reported	Education and life skills	Prevention: To increase children's knowledge of child sexual abuse and self‐protection strategies	The programme consists of three components: teacher training, a parent education meeting, and a 5‐day children's curriculum for the classroom. The information is taught through the use of a picture book, a puppet show, discussion, activities, and roleplay. The parent education meeting informs parents about sexual abuse and provides them with strategies to use with their children to protect them from sexual abuse. In the classroom, children learn to distinguish appropriate from inappropriate touch, to assert their rights to say no to touches that are uncomfortable or inappropriate, and to tell someone if they are uncomfortable about the touch	
Rheingold et al. (2014)	RCT	USA	Sexual abuse	Multiple youth service organisations (e.g., daycare centeres, churches, schools)	All children (0–18 years old)	Child advocacy center	Education and life skills	Prevention and response: To train adults on how to revent (primary prevention), recognise, and respond to child sexual abuse (secondary prevention)	Stewards of Children: A single 2.5 h workshop to train adults in preventing (primary prevention), recognizing, and responding to CSA (secondary prevention). Stewards exists in two formats: (1) in‐person with a facilitator presenting the curriculum and leading discussions and (2) an interactive web‐based training	
Saslawsky (1985)	RCT	USA	Sexual Abuse	Primary/elementary school	Children in Kindergarten to grade 1 (mean age = 6 years old), and grades 5–6 (mean age = 11 years old)	Graduate student led the discussion	Education and life skills	Prevention: To increase children's knowledge of child sexual abuse and self‐protection strategies	A 35 min film, “Touch” was shown to students portraying various abusive incidents and four different skills to prevent sexual abuse, including: say No; yell for help; get away, and tell someone. A discussion follows the film to talk about children's feelings, knowledge gained and reiteration of actions shown in the film	
Snyder (1986)	QED	USA	Sexual abuse	Primary/elementary school	Children in grade 4 (9–10 years old)	Sexual assault counselor	Education and life skills	Prevention: To increase children's knowledge of child sexual assault and self‐protection strategies	A presentation called “Good Secrets, Bad Secrets” was delivered by a sexual assault counsellor. The presentation included sexual assault and safety concepts including general safety skills, distinguishing between appropriate touching from sexual touching, understanding that sexual touches can come from strangers or even someone they know, how to seek help and recognizing an appropriate course of action when in a dangerous situation occurs	
Ssenyonga et al. (2018)	RCT (ongoing)	Uganda	Physical abuse	Primary/elementary school; secondary/high school; before and after school care	Students in grades 8 and 9 (12–17 years old)	Not reported	Education and life skills	Prevention: Aims at improving teacher–student relationships, changing teachers' attitudes and behaviors concerning the use of violent disciplinary measures, and preventing harsh and violent discipline in the school setting	Interaction Competencies with Children for Teachers (ICC‐T) program: Composes a training workshop for teachersrunning for 5.5 days, with 8 h spent in training on each full day. Includes sessions on teacher‐student interactions, maltreatment prevention, effective discipline strategies, identifying and supporting burdened students, and practical implementation. Strategies include presentations, discussions, question and answer sessions, and supervised practical sessions	
Sullivan (1992)	QED	USA	Sexual abuse	Residential school	Children with a hearing impairment (12–16 years old)	The psychotherapy was undertaken by three clinical psychologists, and a supervising psychiatrist with specific training and expertise in the psychology of deafness and fluency in sign language	Response and support services	Treatment: Treating goals included: the alleviation of guilt; treatment of depression; learning to express anger in appropriate and productive ways; providing basic information about normal human sexuality; dealing with sexual preference and homosexual issues; dealing with maltreatment issues; self‐protection techniques; vocabulary to label emotions and feelings; attainment of emotional independence; establishment of a meaningful and stable identity; development of a personal value system, and athe development of a capacity for lasting relationships	Psychotherapy: Each child in the treatment group received 2 hr of individual therapy per week for 36 weeks	
Taal and Edelaar (1997)	QED	Amsterdam	Sexual abuse	Primary/elementary school	Children from grades 6–8 (8–12 years old)	Lessons were delivered by actors and teachers	Norms and values; Education and life skills	Prevention: To increase children's knowledge of child sexual abuse and self‐protection strategies. The main goal of this intervention is that children must be able to identify when they are in an unsafe situation	Right to Security program encompasses major themes including an awareness of “yes” and “no” feelings, an awareness of the right to refuse unwanted sexual interactions with strangers and with trusted others, and seeking help after sexual abuse has occurred. The program consists of 8 lessons including roleplay and discussion sessions	
Taylor (2010)	RCT	USA	Sexual abuse	Middle school	Children in grades 6 and 7 (11–13 years old)	One senior female staff member from a local sexual assault center taught nearly all the classroom sessions. In two of the seven participating school buildings, the regular classroom teacher implemented the curriculum instead of the rape crisis center educator	Norms and values; Education and life skills	Prevention: To improve awareness of abusive behaviors, attitudes toward gender violence, sexual harassment and personal space, and knowledge	Lessons were delivered in five classroom periods, designed to last 40 min each, once per week. The interaction‐based curriculum focused on setting and communicating boundaries in relationships; the formation of deliberate relationships or friendships, and the continuum between friendship and intimacy; the determination of wanted/unwanted behaviors; and the role of the bystander as intervener	
Telljohann et al. (1997)	RCT	USA	Sexual abuse	Primary/elementary school	Children in grade 3 (8–11 years old)	Trained volunteers, who attended a 30‐h training session, and staff members from a social service agency	Norms and values; Education and life skills	Prevention: To increase children's knowledge of child sexual abuse and self‐protection strategies	Sexual Abuse Prevention Program, Third Grade Curriculum: Roleplays, videos, demonstrations, and discussions were used during the knowledge and behavioral skills training. The 2‐h curriculum is covered over a 2‐week period. Key objectives included: listing and describing problems children may encounter; identifying people in family and community support systems; identifying three types of touches (safe, unsafe, secret); identifying the personal safety rules; recognizing that sexual abuse is never a child's fault; recognizing that sexual abuse should not be kept a secret; demonstrating what to do when someone tries to sexually abuse them, and empathizing with youth who have been abused sexually	
Tutty (1997)	RCT	Canada	Sexual abuse	Primary/elementary school	Children from kindergarten to grade 6 (5–11 years old)	Two trainers from the Calgary Communities Against Sexual Assault	Education and life skills	Prevention: To increase children's knowledge of child sexual abuse and self‐protection strategies	Who Do You Tell program: Includes a parent information evening, and a teacher in‐service workshop	
									Two trainers offer the program in small groups (15–20). The programme is delivered to children in two sessions of 45–60 min each, presented on consecutive days. Age‐appropriate materials and videos are matched to the developmental level of the child. The emphasis is on giving information and permission to say no to unwanted touch, the issue of whether this means that children should be suspicious of all touches or adults is also addressed
Van Lieshout et al. (2019)	RCT	The Netherlands	Sexual abuse	Residential care	Boys in residential care (12–18 years old)	Freelance trainers working for Rutgers—Center for Sexual and Reproductive Health and Rights	Norms and values; Education and life skills	Prevention: To increase children's knowledge of child sexual abuse and self‐protection strategies	Make a Move program: Consists of eight weekly meetings in a group setting of six to eight boys of 90 min each. The themes covered by the eight meetings are: men, image, girls, sex, flirting, dating, pleasurable sex, and the future. Each meeting includes several exercises such as roleplay, discussion, and watching short movie clips	
Warden (1997)	QED	UK (Scotland)	Sexual abuse	Primary/elementary school	Children in grade 2 and 6 (6 and 10 years old)	Program is taught by teachers in a classroom setting	Norms and values; Education and life skills	Prevention: To increase children's ability to deal with being bullied, being approached by a stranger, being subject to inappropriate intimacy from a known adult, and any pressure to keep the intimacy secret	Kidscape Safety Training program teaches children general personal safety rules, how to cope with bullies, not to talk to strangers, how and when to say no when presented in an uncomfortable situation. The program is taught using stories, drawing, painting, role plays and discussion. It can be taught in one block or broken down into separate sections to fit the school curriculum	
Weatherley et al. (2012)	QED	Malaysia	Sexual abuse	Primary/elementary school	Children in primary/elementary school (9 years old)	Facilitated by Protect and Save the Children	Education and life skills	Prevention: To increase children's knowledge of child sexual abuse, personal safety and self‐protection strategies	Keeping Me Safe: Six school‐based units that use role playing and games. The purpose was to provide children information about their body, safe and unsafe situations, building a support system, and to impart safety strategies and skills	
White (2018)	RCT	Australia	Sexual abuse	Primary/elementary school	Children in grade 1 (5–7 years old)	Trained facilitators from the non‐profit organization, Act for Kids	Education and life skills	Prevention: This program aims to improve children's interpersonal safety skills in situations ranging from peer bullying to child sexual assault	The Learn to be safe with Emmy and friends™ program: Is held for five, 1‐h weekly sessions. Sessions were on school grounds with trained facilitators, independent to the research team. The content of the five sessions included: (i) Emotion recognition and early warning signs; (ii) Identification of safe/unsafe situations; (iii) Personal space and private body areas; (iv) Safe/unsafe secrets; and (vi) Identification of safe adults and safety networks for disclosure	
Wolfe et al. (1986)	RCT	USA	Physical abuse; sexual abuse	Primary/elementary school	Children in grade 4 and 5 (9–12 years old)	Trained medical students	Education and life skills	Prevention: To increase children's knowledge of and attitudes toward physical and sexual abuse	The program consisted of two, 5‐min skits showing children in uncomfortable situations and how they handled it. Following the skit was a discussion about the nature and prevention of physical and sexual abuse. Purpose is to educate children in child abuse awareness especially in areas including: (1) someone you love and trust can also be abusive; (2) this can cause emotions such as anger, worry, fear and embarrassment; (3) you should tell someone; (4) it is not your fault; and (5) get help immediately	
Wurtele (1986)	RCT	USA	Sexual abuse	Kindergarten; primary/elementary school	Children from Kindergarten to grade 1 (mean age = 6 years old) and children from grade 5 and 6 (mean age = 11 years old)	Female graduate students	Education and life skills	Prevention: To increase children's knowledge of child sexual abuse and self‐protection strategies	Two intervention approaches were compared. (1) A 35 min film called “Touch” presented various abusive incidents, demonstrating four skills to prevent abuse including: (a) say “No”; (b) yelling for help; (c) getting away; and d) telling someone. A 15 min discussion of the film followed, focussing on knowledge that was gained and reiterating the four skills shown in the video. (2) The Behavioral Skills Training Program (BST) consisted of a 50‐min presentation in which the children were taught specific self‐protective skills. These skills included (a) being able to identify the location of one's “private parts,” (b) knowing when it is “okay” or “not okay” to have their private parts touched, and (c) developing the verbal (e.g., saying “No!” in a big voice) and motoric responses (e.g., getting away, telling someone) should an older person wrongfully attempt to violate their bodies. These skills were taught via instruction, modeling, rehearsal, social reinforcement, shaping, and feedback	
Wurtele, Gillispie, et al. (1992)	RCT	USA	Sexual abuse	Preschool; home settings	Children in preschool taking part in the HeadStart program (mean age = 55 months old)	Teachers and parents	Education and life skills	Prevention: To increase children's knowledge of child sexual abuse and self‐protection strategies	The Behavioural Skills Training (BST) is a 4‐day programme delivered at school. children personal safety skills from a behavioral perspective. Working with small groups (ranging in size from 4 to 10), teachers instructed children on various topics related to personal safety. Children practiced discriminating between appropriate and inappropriate touch requests, and were taught (via modeling, rehearsal, praise, and feedback) the appropriate verbal (e.g., say “No”) and motoric responses (e.g., get away, tell someone) to make in the inappropriate situations. An enhanced parent version included a script and accompanying pictures, as well as a packet of stickers and crayons for children to use to color the pictures	
Wurtele, Kast, et al. (1992)	RCT	USA	Sexual abuse	Preschool; home settings	Children in preschool taking part in the HeadStart program (mean age = 57 months old)	Teachers and parents	Education and life skills	Prevention: To increase children's knowledge of child sexual abuse and self‐protection strategies	The Behavioural Skills Training (BST) is a 4‐day programme delivered at school. children personal safety skills from a behavioral perspective. Working with small groups (ranging in size from 4 to 10), teachers instructed children on various topics related to personal safety. Children practiced discriminating between appropriate and inappropriate touch requests, and were taught (via modeling, rehearsal, praise, and feedback) the appropriate verbal (e.g., say “No”) and motoric responses (e.g., get away, tell someone) to make in the inappropriate situations. An enhanced parent version included a script and accompanying pictures, as well as a packet of stickers and crayons for children to use to color the pictures	
Zhang (2014)	RCT	China	Sexual abuse	Preschool	Children in preschool (3–5 years old)	Not described	Education and life skills	Prevention: To increase children's knowledge of child sexual abuse and self‐protection strategies	The Body Safe Training (BST) program was used to teach children personal safety skills. The BST program consists of several stories (each story has an accompanying picture/pictures) about children in innocuous and potentially dangerous situations with various persons. Children practiced differentiating between appropriate and inappropriate requests to touch or look at their private parts, and were taught self‐protection skills including verbal (e.g., say “No!”) and behavioral responses (e.g., leave the situations, tell the trusted persons) in the abusive situations. The self‐protection skills were instructed by using teaching method of instruction, modeling, behavioral rehearsal, social reinforcement, and feedback	
Bucharest Early Intervention Project: Bick et al. (2015); Humphreys et al. (2015); Johnson et al. (2010); Smyke et al. (2010); Troller‐Renfree et al. (2015); Wade et al. (2018)	RCT	Romania	Neglect	Out‐of‐home care	Children in Romanian orphanages (randomised between ages 6–31 months)	N/A	Response and support services	Treatment: To assess foster care as an intervention for children abandoned at or around the time of birth, and placed in one of six institutions for young children in Bucharest, Romania	Family‐based high quality foster care. The families who fostered the children were recruited and screened by the study staff, and throughout the intervention (until each child reached the age of 54 months), and these families were provided significant psychological and economic support for caring for these previously institutionalized children	
Good School Toolkit: Devries, 2015, 2017, 2018; Merril 2018; Knight 2018	RCT	Uganda	Physical abuse, sexual abuse, emotional abuse	Primary/elementary school	Children in primary/elementary school (11–14 years old)	The school‐led activities are coordinated by two lead teacher “protagonists” and two student representatives in each school	Education and life skills; Norms and values	Prevention: To reduce emotional violence, severe physical violence, sexual violence and injuries from school staff to students, as well as emotional, physical and sexual violence between peers, in Ugandan primary schools	The Good School Toolkit: The intervention involves head teachers, administration, teachers, and students. Targets multiple levels within the schools with multilayered training, processes, and school‐led activities. The Toolkit materials consist of books, booklets, posters, and facilitation guides for about 60 different activities. The Toolkit itself has six steps, which are designed to be implemented in sequence. These relate to creating a better learning environment, respecting each other, creating opportunities for students to participate in decision‐making processes, understanding power relationships, using non‐violent discipline, improving classroom management techniques, and promoting responsive school governance. The protagonists and head teachers receive training at programme initiation. The schools receive one‐on‐one support visits and phone calls from Raising Voices staff throughout implementation	
Children Need to Know: Personal Safety Training Program: Krazier 1988; Fryer et al. (1987)	RCT	USA	Sexual abuse	Primary/elementary school	Children from Kindergarten to grade 2 (5–8 years old)	Not reported	Education and life skills	Prevention: The program aimed to improve children's awareness of their own personal safety, and teach self‐protective behaviours	Children Need To Know: Personal Safety Training Program is a scripted primary prevention programme delivered in a group classroom setting. It is an 8‐day program, with 20 min presentations each day, addressing misconceptions about personal safety. Taught through example, discussion, and extensive roleplay allowing children to practise a set of rules	

### Characteristics of systematic reviews

**Table:**

Author, publication year	Review aim	AMSTAR 2	Study designs	Maltreatment type/s	Settings	Child population of interest and age	Intervention type	Interventions of interest included
Heidotting (1996)	The purpose of this review was to investigate the effectiveness of school‐based sexual abuse and personal safety prevention programmes for children by objectively and empirically synthesizing primary research evidence using the techniques of metaanalysis	Low	Treatment and nonequivalent control group; repeated measures study designs	Sexual abuse	Early childhood education settings; primary/elementary school	Children attending pre‐school and primary/elementary school (3–11 years)	Prevention	School‐based sexual abuse and personal safety prevention programmes for children
Hermenau et al. (2017)	This systematic review investigated the effects of structural interventions and caregiver trainings on child development in institutional environments	Low	Multiple study designs	Physical abuse; emotional abuse; neglect	Residential care/orphanages	Children aged 0–17 years living full time in child care institutions in any part of the world	Response	Interventions implemented within institutions aiming to change the context of the institutions as well as the ways in which caregivers interacted with the children; and interventions that aimed to improve the children's development and living conditions by employing one or more intervention components (i.e., caregiver training or supervision, structural changes, or additional stimulation)
McKibbin (2017)	To conduct a scoping exercise of the evidence about preventing harmful sexual behavior and child sexual Exploitation problems with the intent of summarising and disseminating knowledge to policy‐makers, practitioners and researchers	Low	Multiple study designs (including: peer‐reviewed journal articles; government reports; presentation transcripts; literature reviews; qualitative report; government inquiry report; submission to government inquiry; consultation paper for government inquiry; guideline; and educational resource)	Sexual abuse	Residential care	Children and young people living in residential care	Prevention Treatment	Prevention programmes (education); upskilling workers; targets grooming and problematic sexual behaviour (perpetration and/or victimisation); early intervention/recognition; holistic response; treatment, safety planning and placement management
Pitts (2015)	The aim of this evidence review is to determine what is known from the existing literature about the efficacy of pre‐school child sexual abuse prevention programmes	Low	Not reported	Sexual abuse	Centre‐based early childhood education and care settings	Pre‐school children (age not specifically reported)	Prevention	Child sexual abuse prevention programmes for pre‐schoolers
Radford et al. (2017)	The Rapid Evidence Assessment was to identify what is known in countries other than England and Wales about best practice and “what works” to prevent, identify and respond to child sexual abuse with an institutional dimension	Low	Systematic reviews; qualitative; qualitative study designs	Sexual abuse	Multiple settings	Not specified other than “children”	Prevention Disclosure Response Treatment	Types of institutional response to child sexual abuse: Those aimed at primary prevention, stopping child sexual abuse and/or sexual exploitation happening in the first place. Those aimed at improving child protection through better identification, disclosure, reporting and responses, enabling children to disclose abuse, improving recognition among those in contact with children. Those aimed at better control and management of offenders, especially ensuring they do not reoffend. Those aimed at providing better support for victims and survivors, aiding recovery and undoing the harm and injustices caused to victims and survivors and their families
Quadara et al. (2015)	Overall aims of this project were to consider the specific dynamics of child sexual abuse and their implications for prevention and early intervention. Specific aims: map current prevention, early intervention and therapeutic responses against this analysis; and assess key points of prevention and intervention in light of identified risk factors and facilitators of child sexual abuse and apparent gaps in prevention	Low	Unclear/not reported	Sexual abuse	Multiple settings	Not specified other than “children”	Prevention Disclosure Response	Prevention programmes: Primary prevention, preventing recidivism, integrated treatment, current Australian programmes
Ricardo et al. (2011)	To investigate the effectiveness of interventions for preventing boys' and young men's use of sexual violence, including: increasing gender‐equitable attitudes, bystander intentions, and other attitudes and behaviors	Low	Randomised conrolled trials; quasi‐experimental designs	Sexual abuse; physical abuse; emotional abuse	Middle school; secondary/high school	Adolescent boys and young men aged 12–19 years	Response Prevention	Interventions included are those designed to prevent boys and young men's use of rape and other forms of sexual violence, or to change those attitudes about gender, violence, and/or intimate relationships with women that are correlated with boys' and young men's use of rape and other forms of sexual violence. Interventions designed to increase boys' and young men's positive bystander attitudes and behaviors are also included
Sherr et al. (2017)	Part of a systematic review series addressing violence and abuse experiences in institutionalised care, this review explores interventions to reduce such violence or abuse	Low	Included a comparison group not exposed to institutional care or other comparison group; repeated measures study designs	Physical abuse; neglect; emotional abuse	Residential care; institutional care	Children within institutional care	Response Prevention	Studies that reported on the use of an intervention to reduce maltreatment within institutional care
South et al. (2015)	The aim of this scoping review was to map evaluations of out‐of‐home care (OOHC) practice elements that aim to prevent child sexual abuse in OOHC.	Low	No restriction on study design	Sexual abuse	Out‐of‐home care settings; foster care	Any child in overnight care between the ages of 0–17 years, where the state or territory makes a financial payment, or where a financial payment has been offered but has been declined by the carer	Prevention Response	Any type of program, service or practice element that contributes to decreasing the occurrence or preventing child sexual abuse in OOHC
Topping and Barron (2009)	To systematically and critically review evidence from 1990 onward for the effectiveness of programs based in schools for primary prevention of child sexual abuse	Low	Randomised controlled trials; pre and/or post test, with and without a control group	Sexual abuse	Kindergarten; primary/elementary school; high school	Children in kindergarten through year 10 (aged 5–16 years) in primary/elementary or secondary/high school	Prevention	Programs that were delivered from a school base, focused on working with children (not parents or teachers), focused on child sexual abuse primary prevention, did not focus on physical, emotional, or ritualistic abuse or neglect, and did not focus only on children and young people with learning difficulties
Walsh et al. (2015); Zwi et al. (2007)	To systematically assess evidence of the effectiveness of school‐based education programmes for the prevention of child sexual abuse	High	Randomised controlled trials; cluster‐randomised controlled trials; quasi‐experimental designs	Sexual abuse	Primary/elementary school; Secondary/high school	Children (aged 5–12 years) and adolescents (aged 13–18 years) attending primary/elementary or secondary/high schools	Prevention	Included interventions were school‐based education programmes focusing on knowldge of sexual abuse and sexual abuse prevention concepts, or skill acquisition in protective behaviours, or both

### Risk of bias assessments for included RCTs

**Table:**

Study (Author and year)	Overall risk of bias	Domain 1. Randomisation process	Domain 2. Deviations from intended interventions	Domain 3. Missing outcome data	Domain 4. Measurement of the outcome	Domain 5. Selection of the reported result
Barron and Topping (2013)	Some concerns	Some concerns	Low risk	Low risk	Low risk	Some concerns
Bick et al. (2015)	Some concerns	Low risk	Some concerns	Low risk	Low risk	Some concerns
Blumberg (1991)	High risk	Some concerns	High risk	High risk	High risk	Some concerns
Bustamante (2019)	High risk	Low risk	High risk	High risk	Some concerns	Some concerns
Cecen‐Erogul and Hasirci (2013)	Some concerns	Some concerns	Some concerns	Low risk	Low risk	Some concerns
Chen et al. (2012)	Some concerns	Some concerns	Some concerns	Low risk	Some concerns	Some concerns
Citak Tunc et al. (2018)	Some concerns	Some concerns	Low risk	Low risk	Low risk	Some concerns
Conte (1985)	Some concerns	Low risk	High risk	Low risk	Low risk	Some concerns
Crowley (1989)	Some concerns	Some concerns	Low risk	Some concerns	Some concerns	Some concerns
Daigneault et al. (2012)	Some concerns	Low risk	Some concerns	Low risk	Low risk	Some concerns
Daigneault et al. (2015)	High risk	Some concerns	Some concerns	High risk	High risk	Some concerns
Dake et al. (2003)	Some concerns	Some concerns	Some concerns	Some concerns	Some concerns	Some concerns
del Campo Sánchez and Sánchez (2006)	Some concerns	Some concerns	Some concerns	Low risk	Low risk	Some concerns
Devries et al. (2015)	High risk	Low risk	Some concerns	Low risk	High risk	Some concerns
Devries et al. (2017)	High risk	Low risk	Some concerns	Low risk	High risk	Some concerns
Devries et al. (2018)	High risk	Low risk	Some concerns	High risk	High risk	Some concerns
Edwards et al. (2019)	High risk	Some concerns	Some concerns	High risk	High risk	Some concerns
Feldmann et al. (2018)	High risk	Some concerns	Low risk	Low risk	High risk	Some concerns
Fryer et al. (1987)	Some concerns	Low risk	Some concerns	Low risk	Some concerns	Some concerns
Grendel (1991)	Some concerns	Low risk	Some concerns	Low risk	Low risk	Some concerns
Gushwa et al. (2018)	High risk	Some concerns	Low risk	High risk	Some concerns	Some concerns
Harvey et al. (1988)	Some concerns	Some concerns	Some concerns	Low risk	Low risk	Some concerns
Hazzard et al. (1991)	Some concerns	Some concerns	Some concerns	Low risk	Some concerns	Some concerns
Humphreys et al. (2015)	Some concerns	Low risk	Some concerns	Low risk	Some concerns	Some concerns
Jin et al. (2017)	Some concerns	Some concerns	Some concerns	Low risk	Some concerns	Some concerns
Johnson et al. (2010)	Some concerns	Low risk	Some concerns	Low risk	Low risk	Some concerns
Knight et al. (2018)	High risk	Low risk	Some concerns	Low risk	High risk	Some concerns
Krahe (2009)	High risk	Low risk	Some concerns	Low risk	High risk	Some concerns
Kraizer et al., 1988	High risk	Some concerns	High risk	High risk	High risk	Some concerns
Merril (2018)	High risk	Low risk	Some concerns	Some concerns	High risk of bias	Some concerns
Nkuba et al. (2018)	High risk	Some concerns	High risk	High risk	Some concerns	Some concerns
Oldfield et al. (1996)	Some concerns	Some concerns	Some concerns	Low risk	Low risk	Some concerns
Pulido et al. (2015)	Some concerns	Some concerns	Low risk	Low risk	Low risk	Some concerns
Ratto and Bogat (1990)	High risk	Some concerns	High risk	Some concerns	High risk	Some concerns
Rheingold et al. (2014)	Some concerns	Low risk	Some concerns	Low risk	Some concerns	Some concerns
Saslawsky (1976)	Some concerns	Low risk	Some concerns	Low risk	Low risk	Some concerns
Smyke et al. (2010)	Some concerns	Low risk	Some concerns	Some concerns	Low risk	Some concerns
Taylor et al. (2010)	High risk	Low risk	Some concerns	High risk	High risk	Some concerns
Telljohann et al. (1997)	Some concerns	Some concerns	Some concerns	Some concerns	Low risk	Some concerns
Troller‐Renfree et al. (2015)	Some concerns	Low risk	Some concerns	Some concerns	Low risk	Some concerns
Tutty, 1997	High risk	Some concerns	Low risk	Low risk	High risk	Some concerns
Van Lieshout et al. (2019)	High risk	Some concerns	High risk	High risk	Some concerns	Some concerns
Wade et al. (2018)	Some concerns	Low risk	Some concerns	Some concerns	Low risk of bias	Some concerns
White et al. (2018)	Some concerns	Low risk	Low risk	Low risk	Low risk	Some concerns
Wolfe et al. (1986)	Some concerns	Some concerns	Some Concerns	Low risk	Low risk	Some concerns
Wurtele et al. (1986)	Some concerns	Low risk	Some concerns	Low risk	Low risk	Some concerns
Wurtele, Gillispie, et al. (1992)	Some concerns	Low risk	Some concerns	Low risk	Low risk	Some concerns
Wurtele, Kast, et al. (1992)	High risk	Some concerns	High Risk	High risk	Low risk	Some concerns
**Zhang** (2014)	Some concerns	Some concerns	Some concerns	Low risk	Some Concerns	Some concerns
